# Supplementary figures and images for: Protein visualization and manipulation in Drosophila through the use of epitope tags recognized by nanobodies
Source: eLife. 2022 Jan 25;11:e74326. doi: 10.7554/eLife.74326 (PMC8853664; doi:10.7554/eLife.74326)

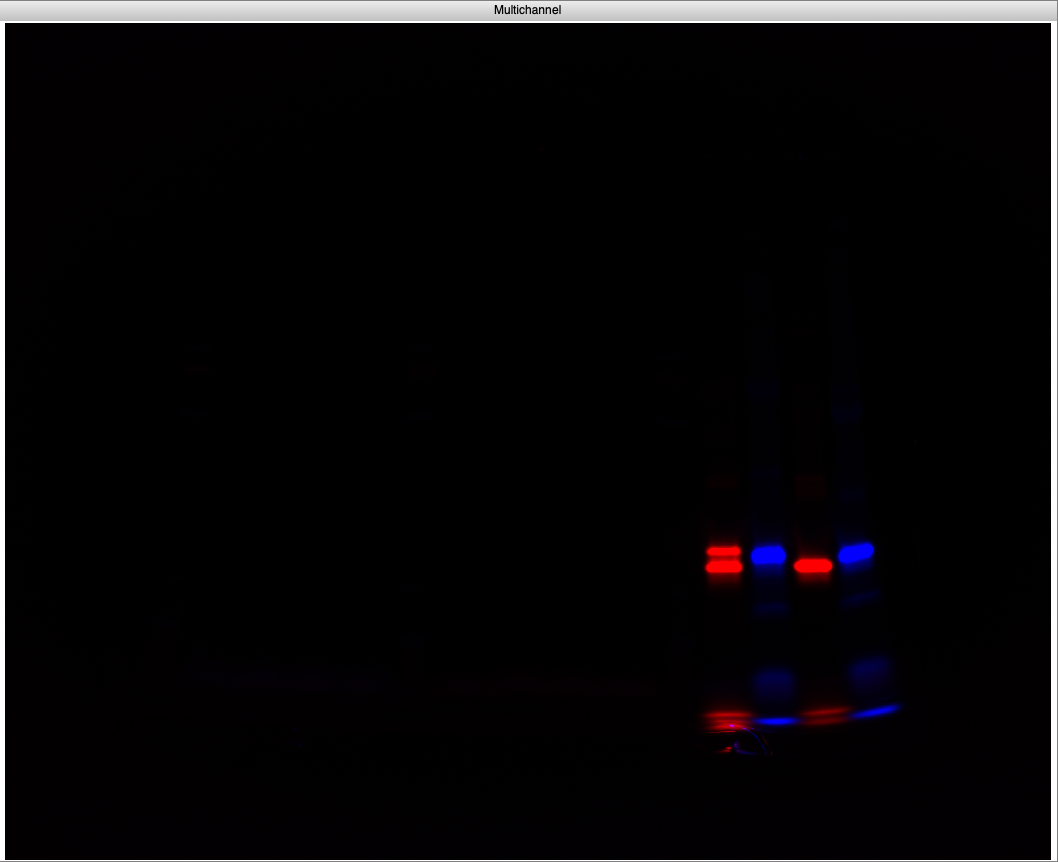

Supplement: Figure 2—figure supplement 1—source data 1. [file elife-74326-fig2-figsupp1-data1.zip › Figure 2í¬figure supplement 1-source data/Figure 2í¬figure supplement 1-A1.tif]

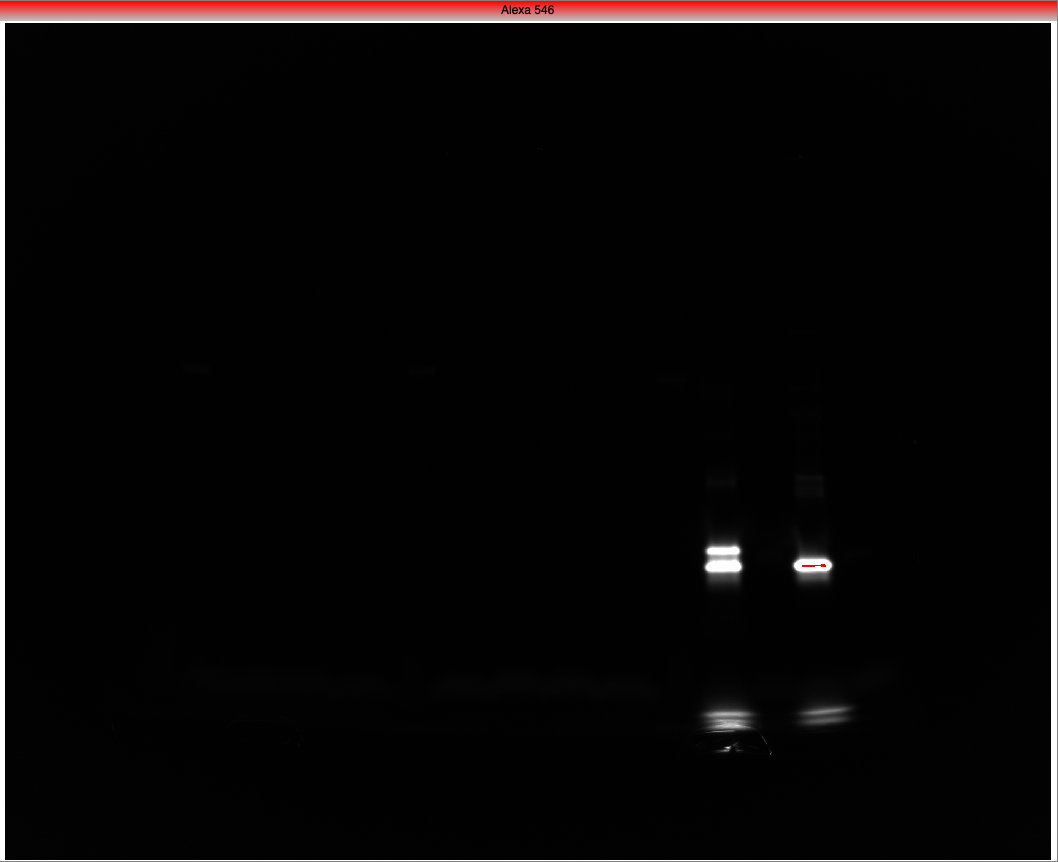

Supplement: Figure 2—figure supplement 1—source data 1. [file elife-74326-fig2-figsupp1-data1.zip › Figure 2í¬figure supplement 1-source data/Figure 2í¬figure supplement 1-A2.tif]

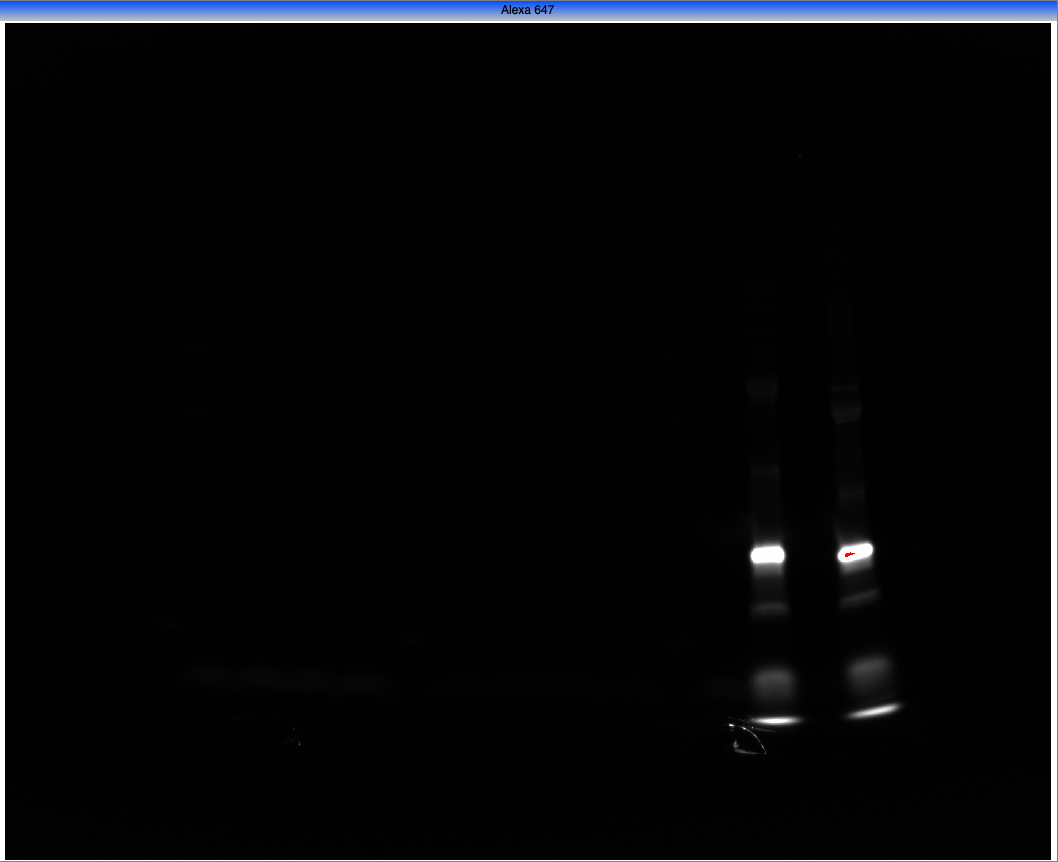

Supplement: Figure 2—figure supplement 1—source data 1. [file elife-74326-fig2-figsupp1-data1.zip › Figure 2í¬figure supplement 1-source data/Figure 2í¬figure supplement 1-A3.tif]

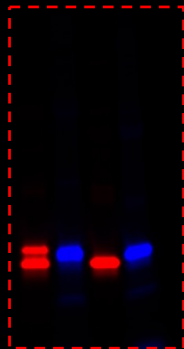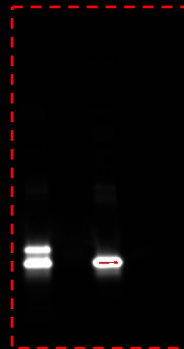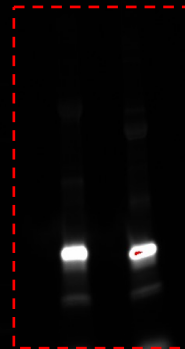

Supplement: Figure 2—figure supplement 1—source data 1. [file elife-74326-fig2-figsupp1-data1.zip › Figure 2í¬figure supplement 1-source data/Figure 2í¬figure supplement 1-A_crop.pdf]

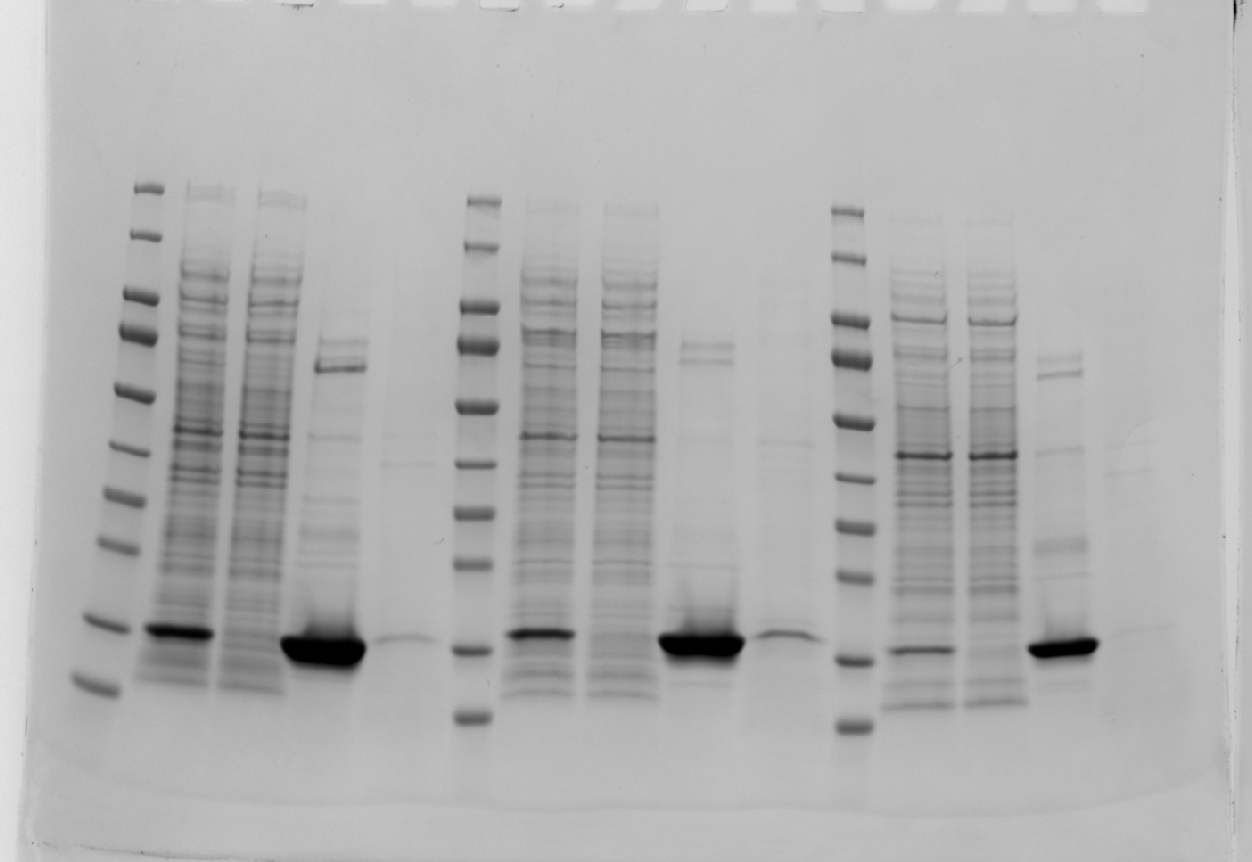

Supplement: Figure 2—figure supplement 1—source data 1. [file elife-74326-fig2-figsupp1-data1.zip › Figure 2í¬figure supplement 1-source data/Figure 2í¬figure supplement 1-B.tif]

NbVHH05-ALFA-His

Nb127D01-ALFA-His

Input  
FT  
Elution

Input  
FT  
Elution

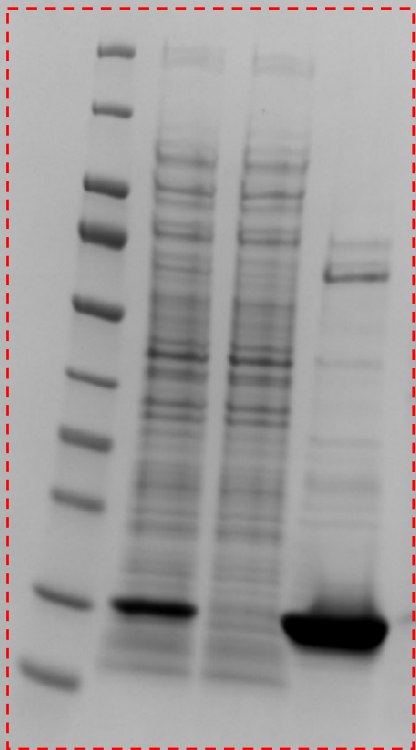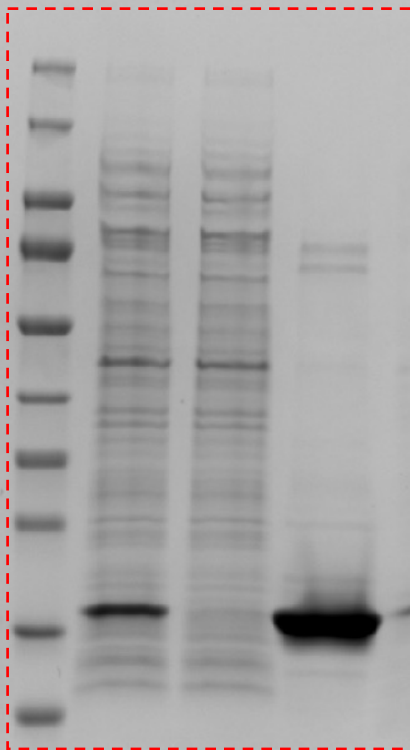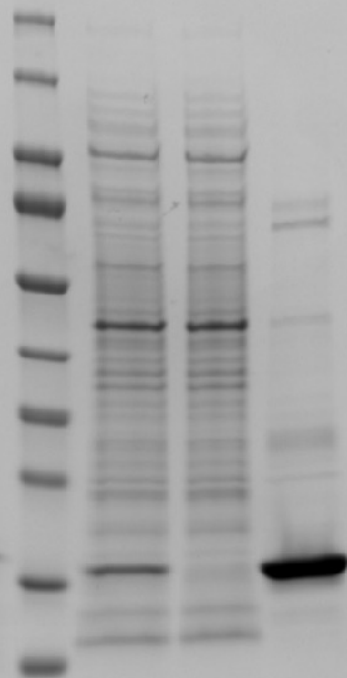

Supplement: Figure 2—figure supplement 1—source data 1. [file elife-74326-fig2-figsupp1-data1.zip › Figure 2í¬figure supplement 1-source data/Figure 2í¬figure supplement 1-B_crop.pdf]

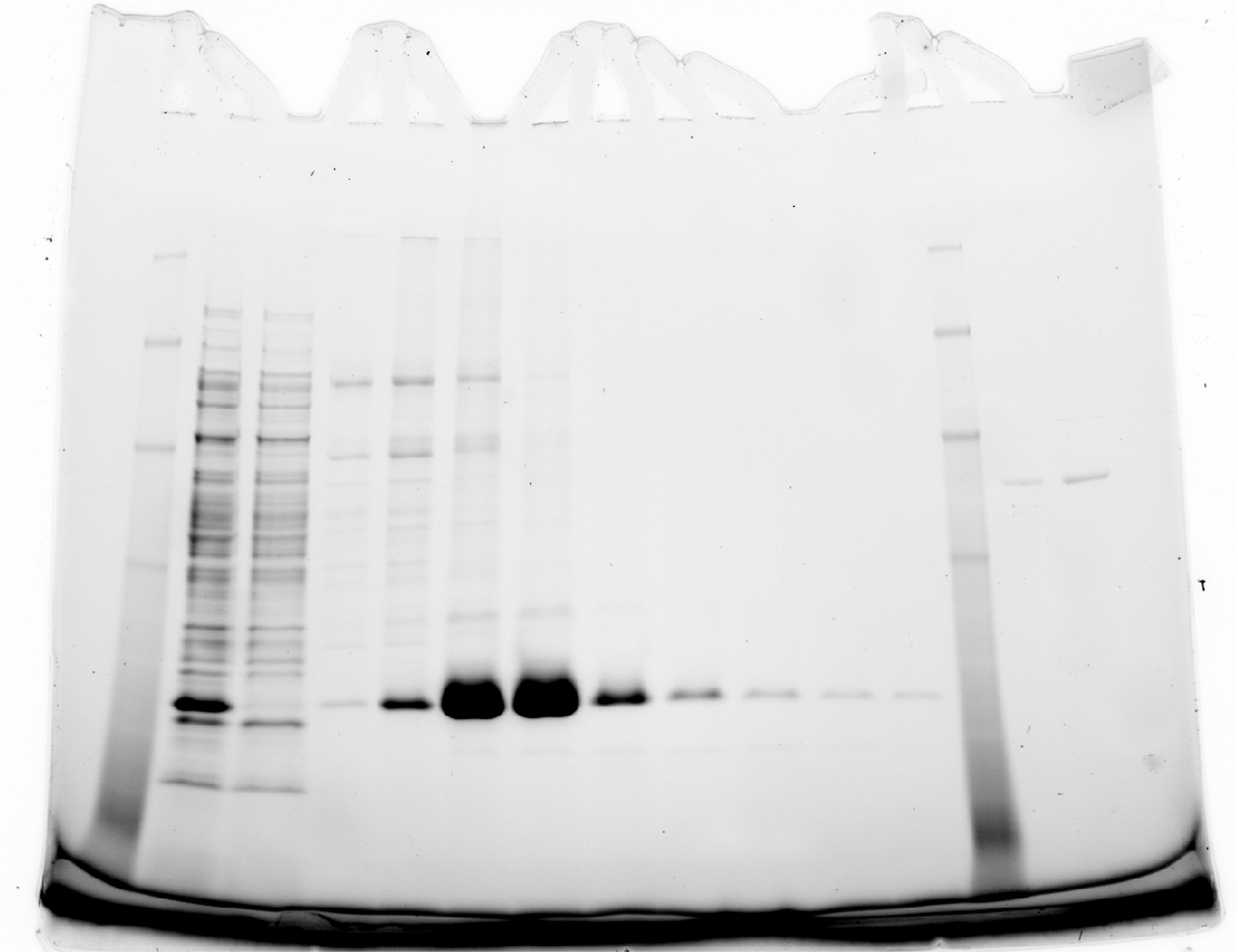

Supplement: Figure 2—figure supplement 1—source data 1. [file elife-74326-fig2-figsupp1-data1.zip › Figure 2í¬figure supplement 1-source data/Figure 2í¬figure supplement 1-C1.jpg]

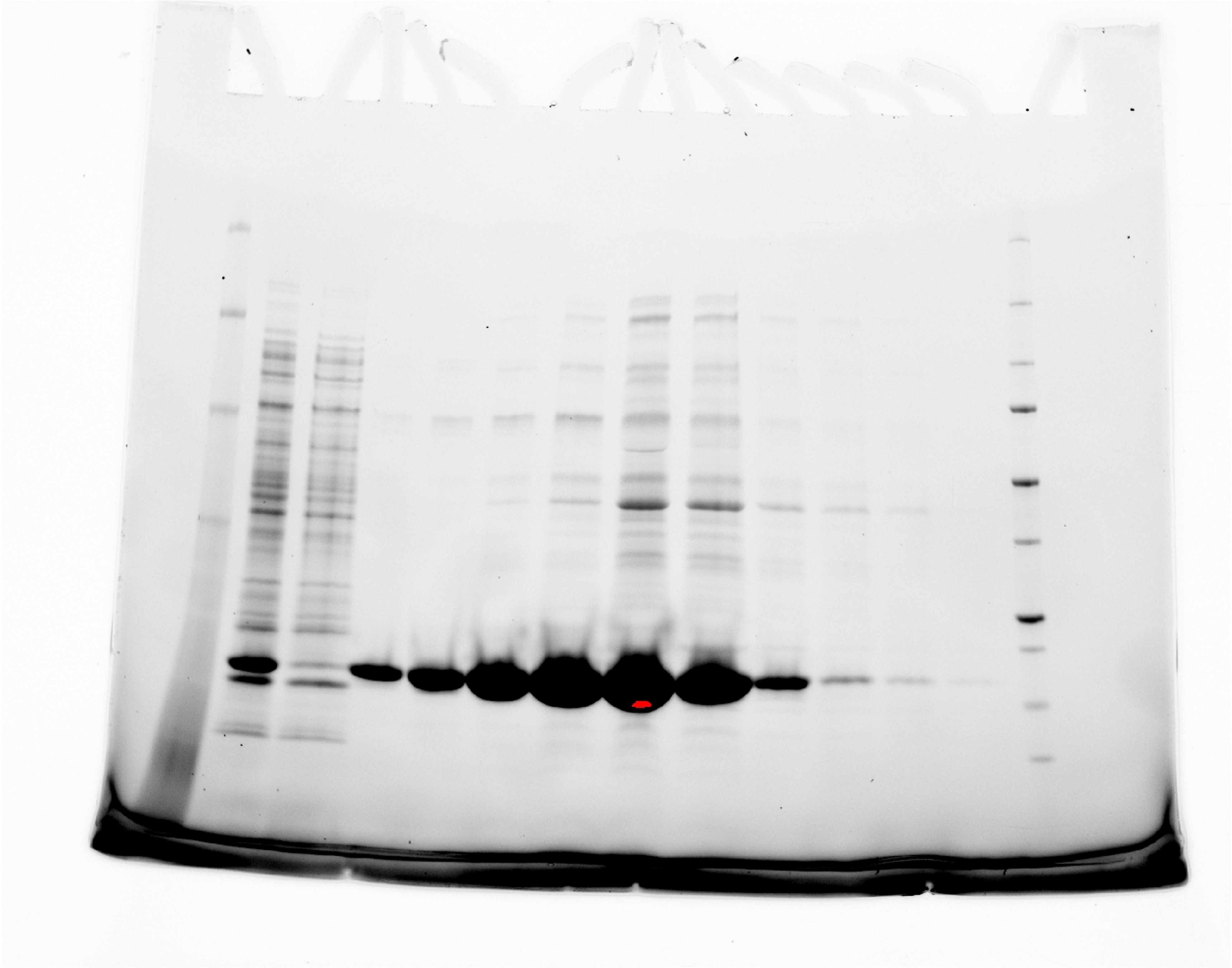

Supplement: Figure 2—figure supplement 1—source data 1. [file elife-74326-fig2-figsupp1-data1.zip › Figure 2í¬figure supplement 1-source data/Figure 2í¬figure supplement 1-C2.jpg]

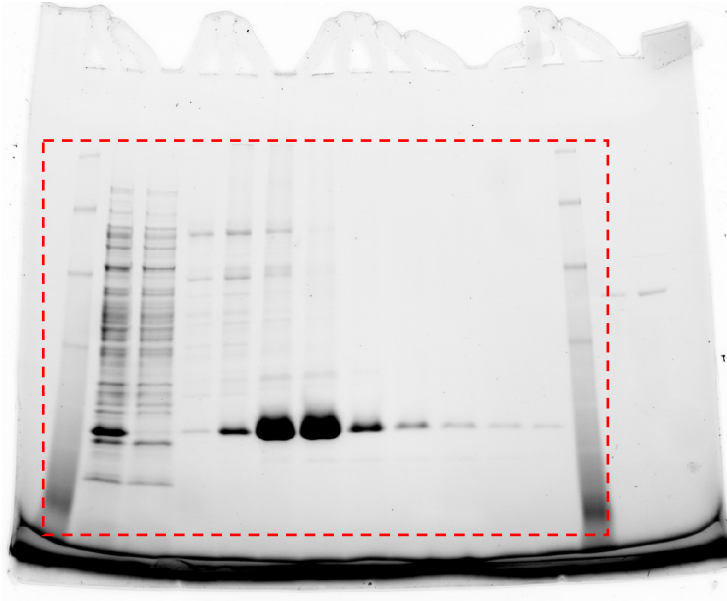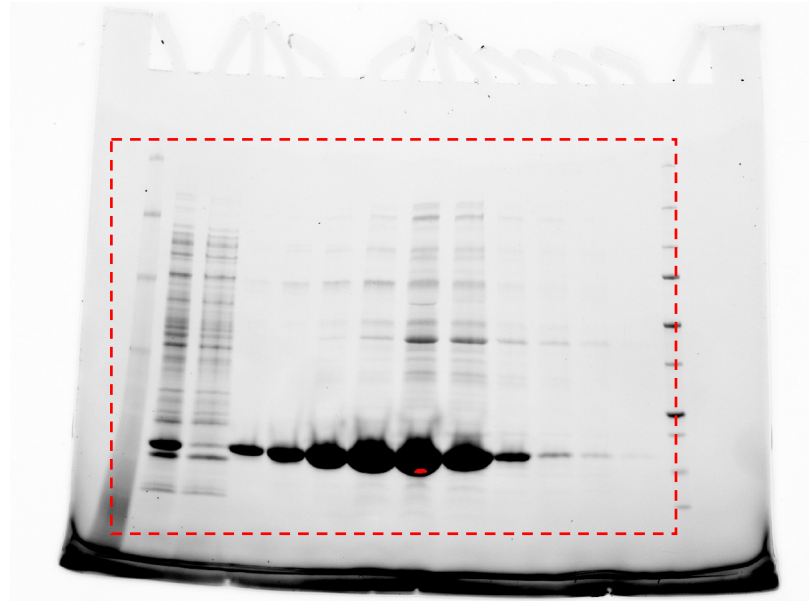

Supplement: Figure 2—figure supplement 1—source data 1. [file elife-74326-fig2-figsupp1-data1.zip › Figure 2í¬figure supplement 1-source data/Figure 2í¬figure supplement 1-C_crop.pdf]

2020.6.2 - ①

NbVHH05

Nb127D01

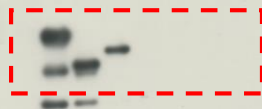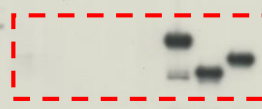

2020.6.2

anti-tubulin

20S

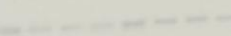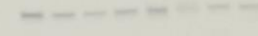

60S

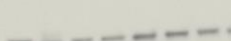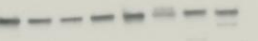

120S

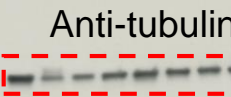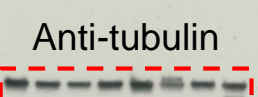

Supplement: Figure 2—figure supplement 3—source data 1. [file elife-74326-fig2-figsupp3-data1.zip › Figure 2í¬figure supplement 3-source data/Figure 2í¬figure supplement 1-B.pdf]

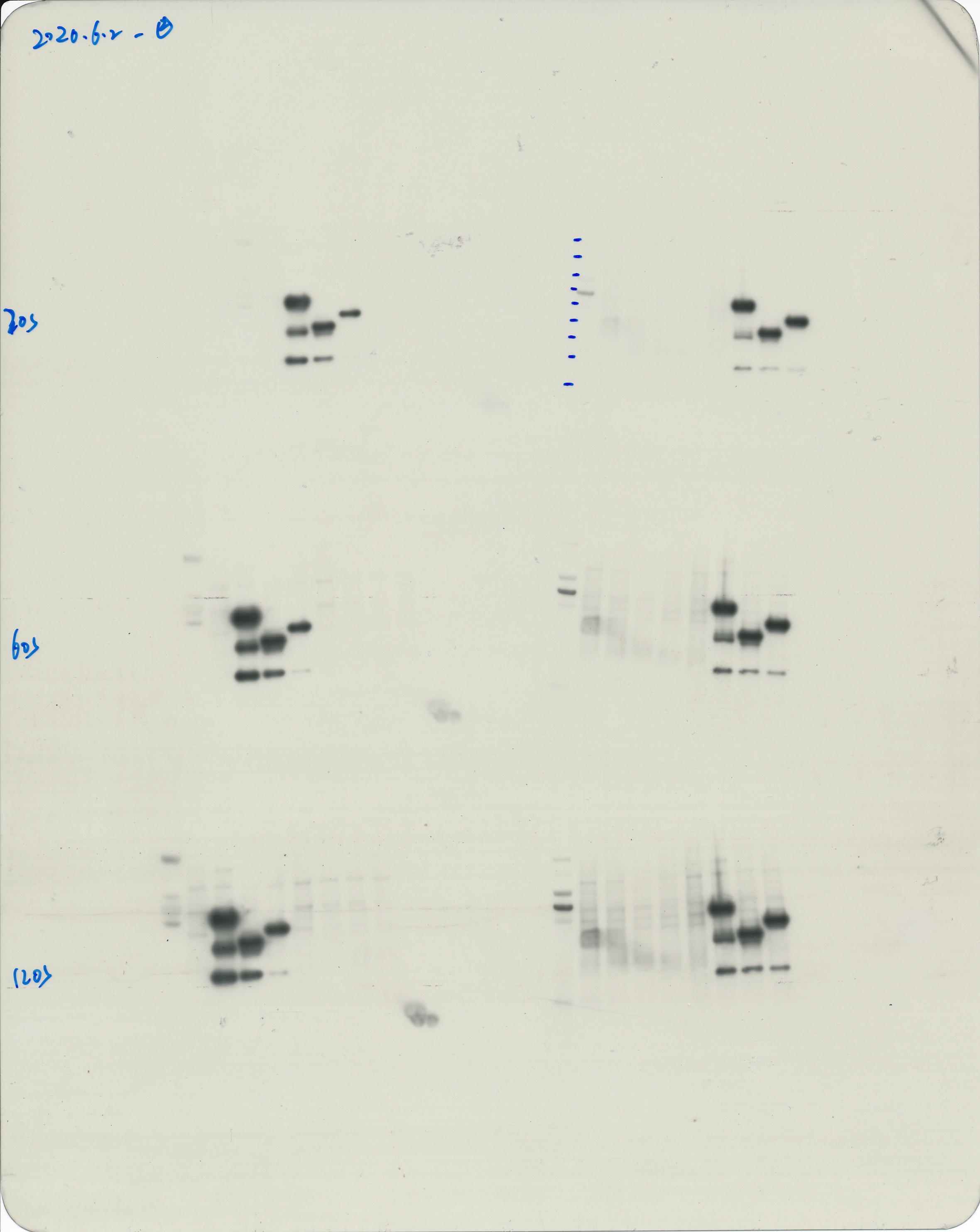

Supplement: Figure 2—figure supplement 3—source data 1. [file elife-74326-fig2-figsupp3-data1.zip › Figure 2í¬figure supplement 3-source data/Figure 2í¬figure supplement 1-raw.B1.jpg]

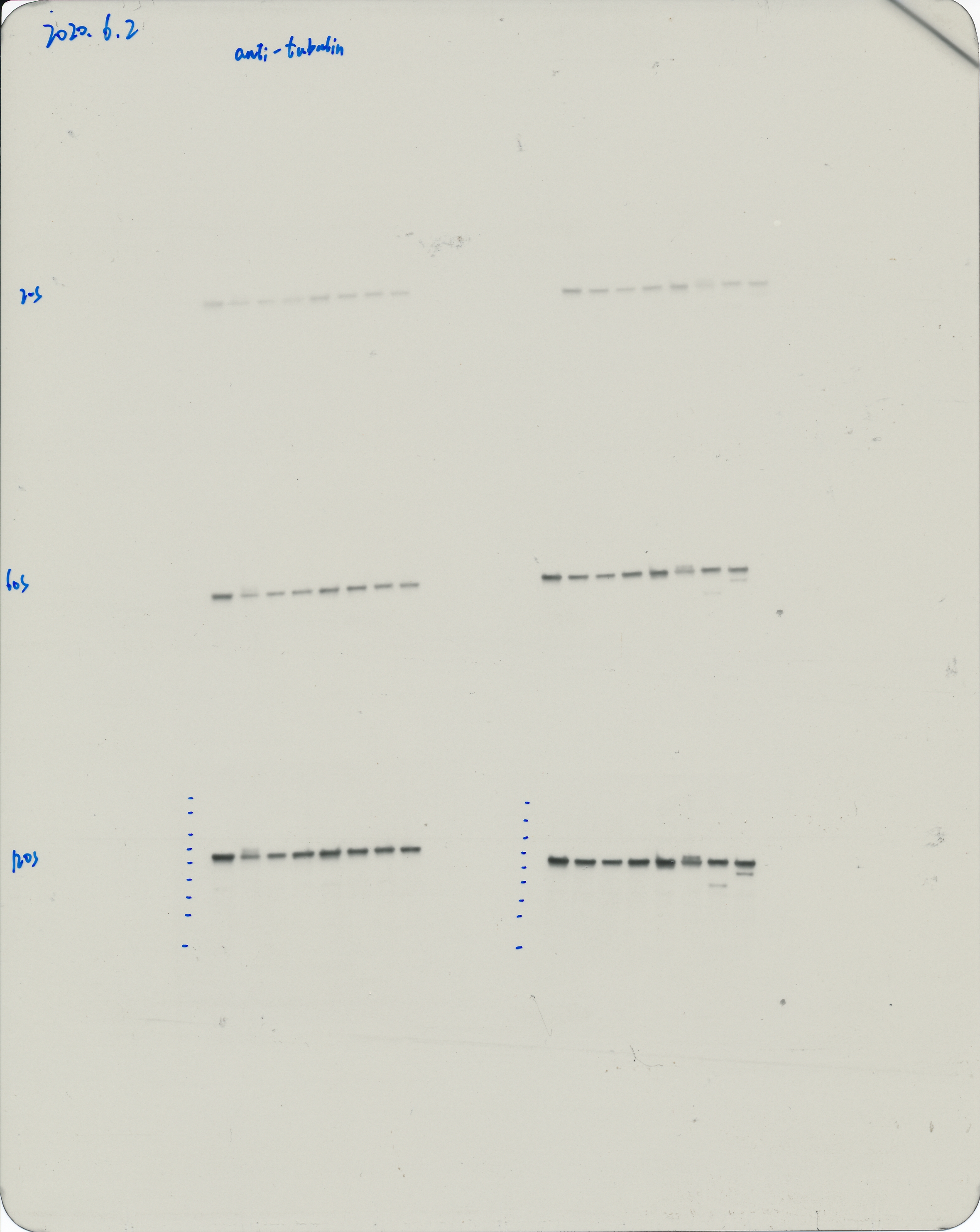

Supplement: Figure 2—figure supplement 3—source data 1. [file elife-74326-fig2-figsupp3-data1.zip › Figure 2í¬figure supplement 3-source data/Figure 2í¬figure supplement 1-raw.B2.jpg]

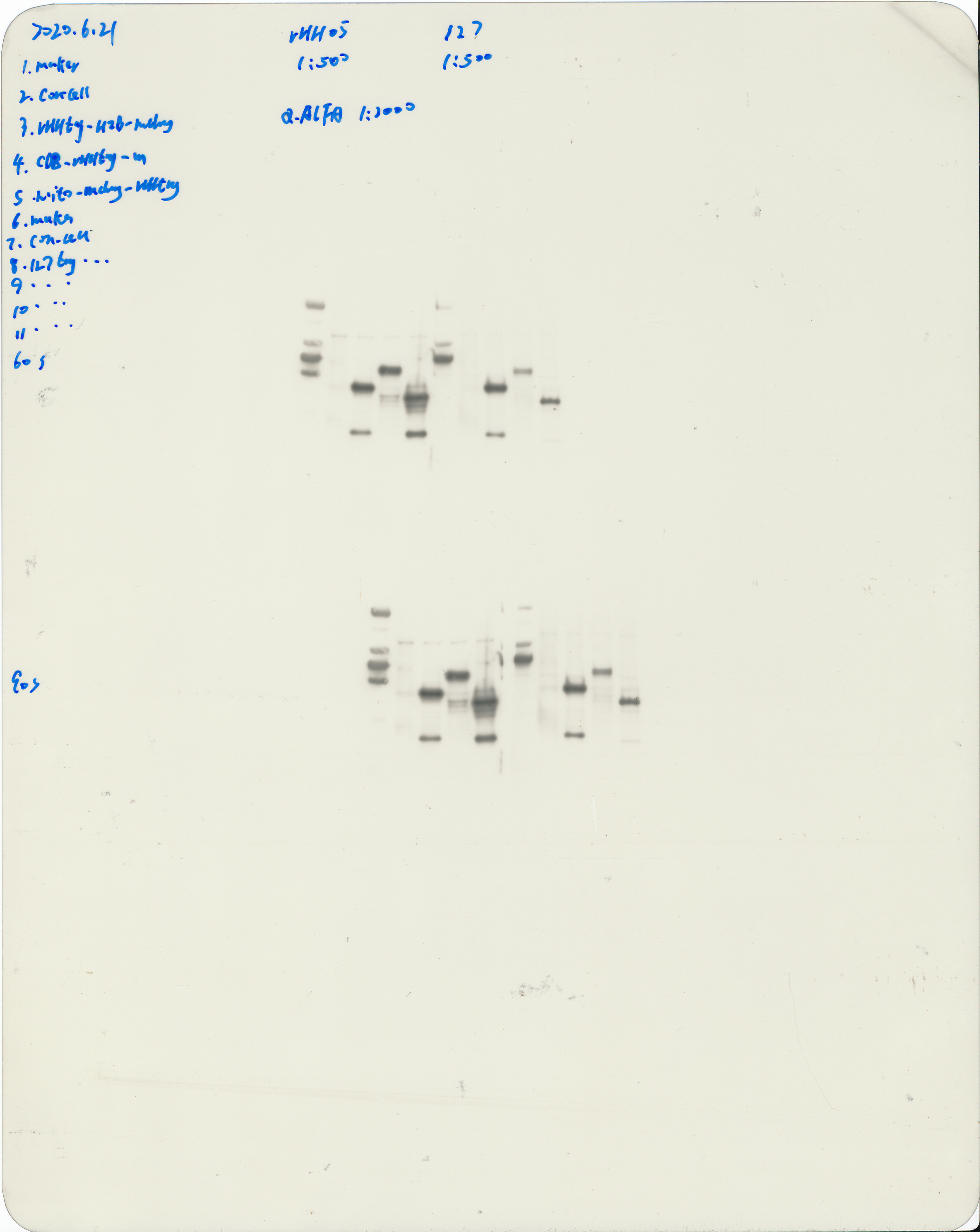

Supplement: Figure 3—source data 1. [file elife-74326-fig3-data1.zip › Figure 3í¬source data/Figure 3A_raw_1.tif]

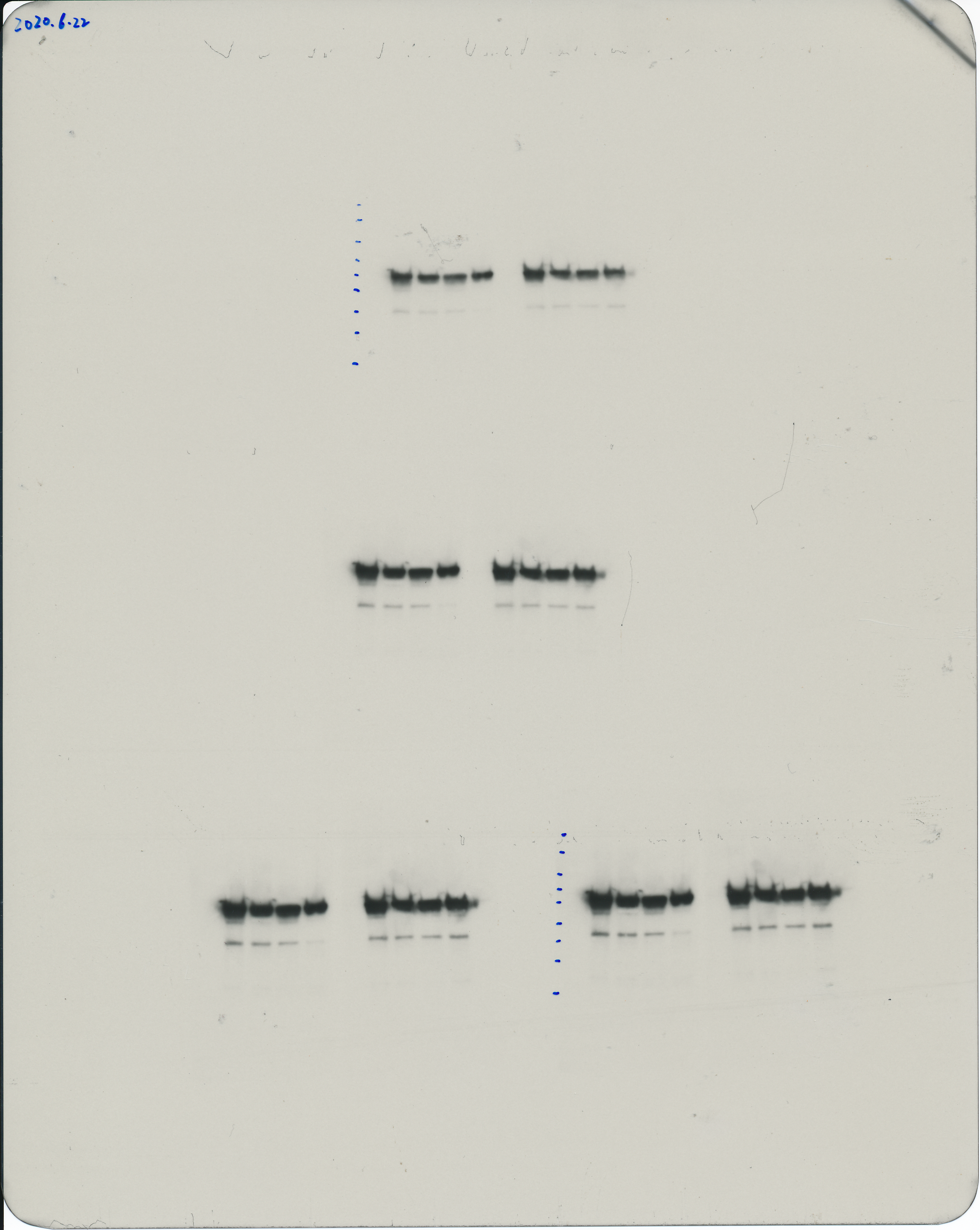

Supplement: Figure 3—source data 1. [file elife-74326-fig3-data1.zip › Figure 3í¬source data/Figure 3A_raw_2.tif]

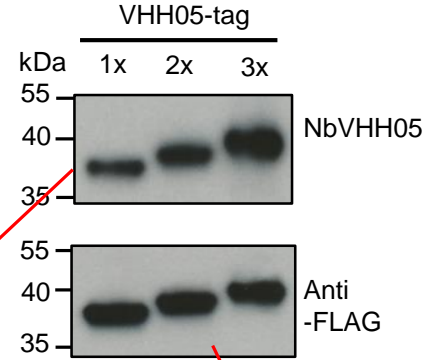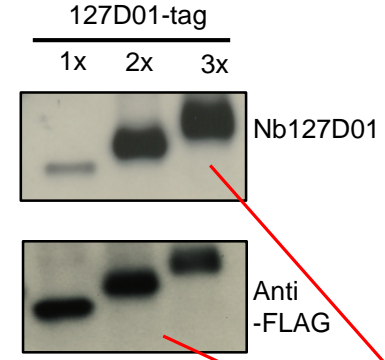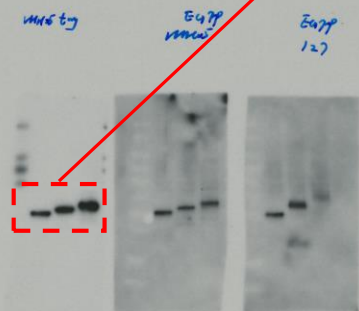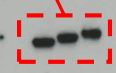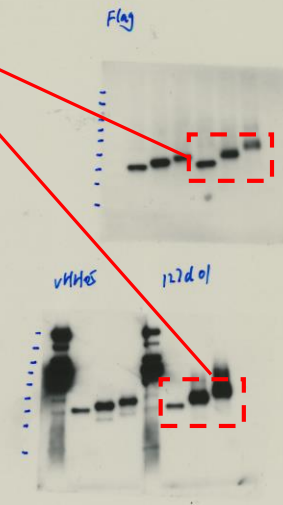

Supplement: Figure 3—source data 1. [file elife-74326-fig3-data1.zip › Figure 3í¬source data/Figure 3B_Crop.pdf]

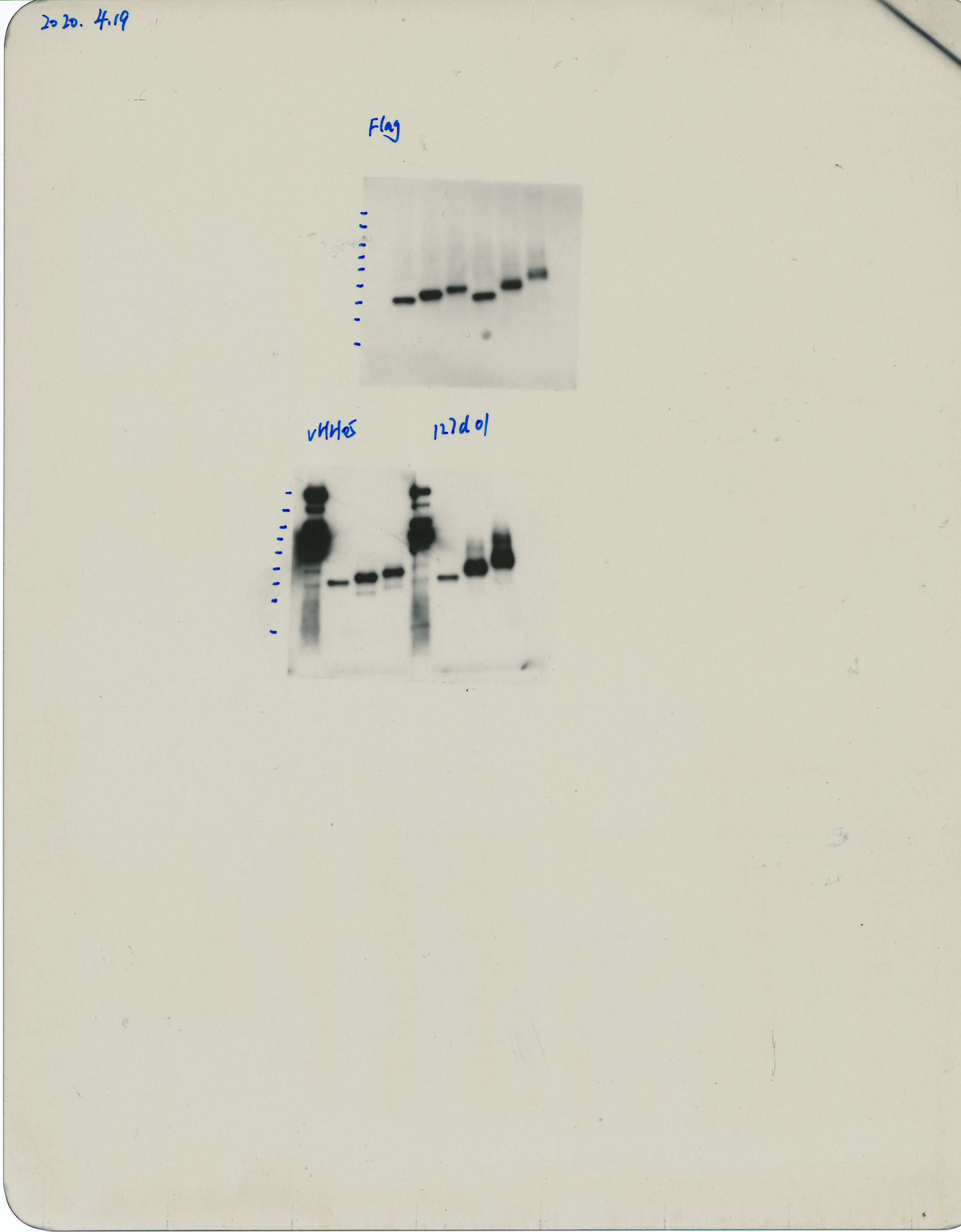

Supplement: Figure 3—source data 1. [file elife-74326-fig3-data1.zip › Figure 3í¬source data/Figure 3B_raw_1.tif]

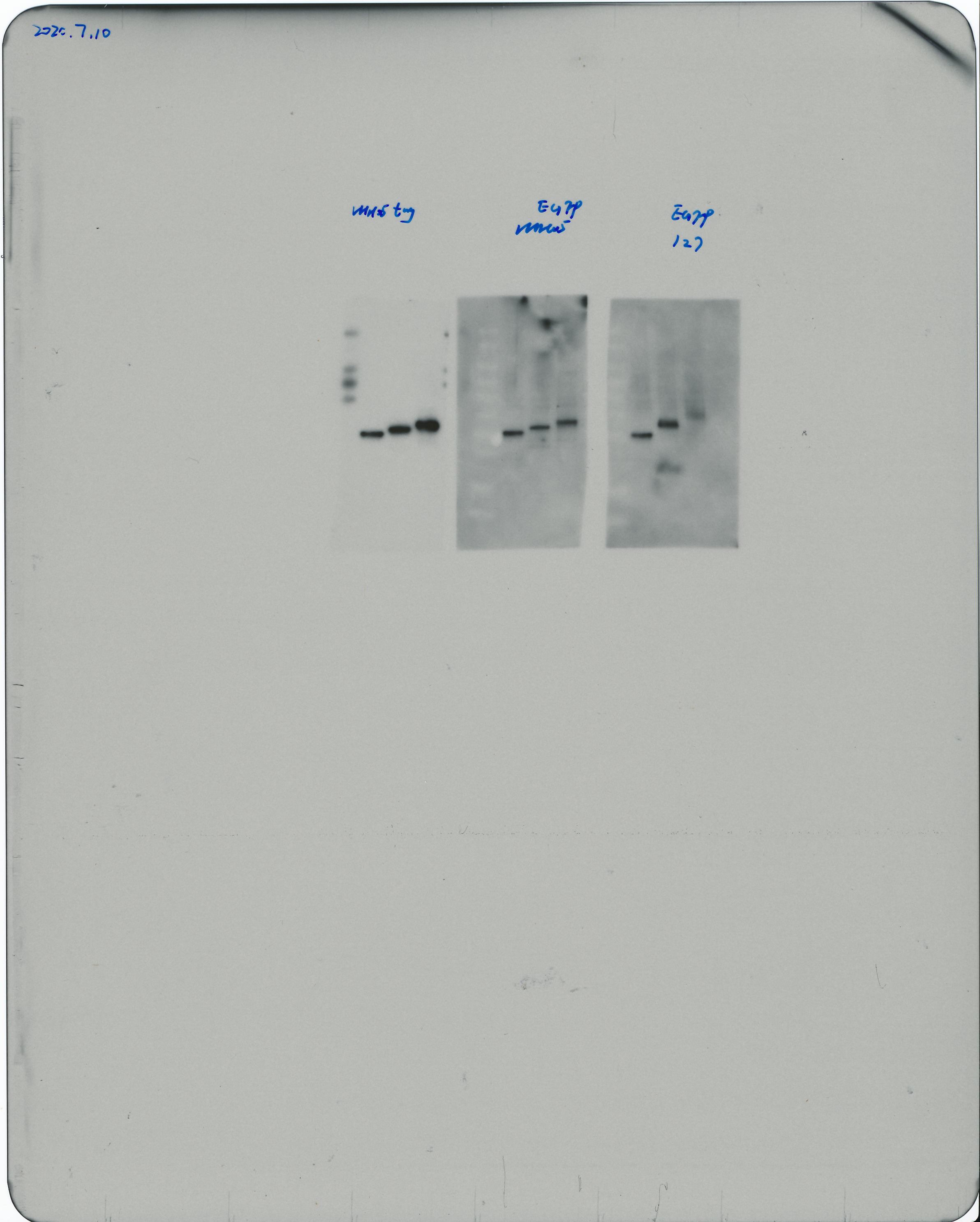

Supplement: Figure 3—source data 1. [file elife-74326-fig3-data1.zip › Figure 3í¬source data/Figure 3B_raw_2.tif]

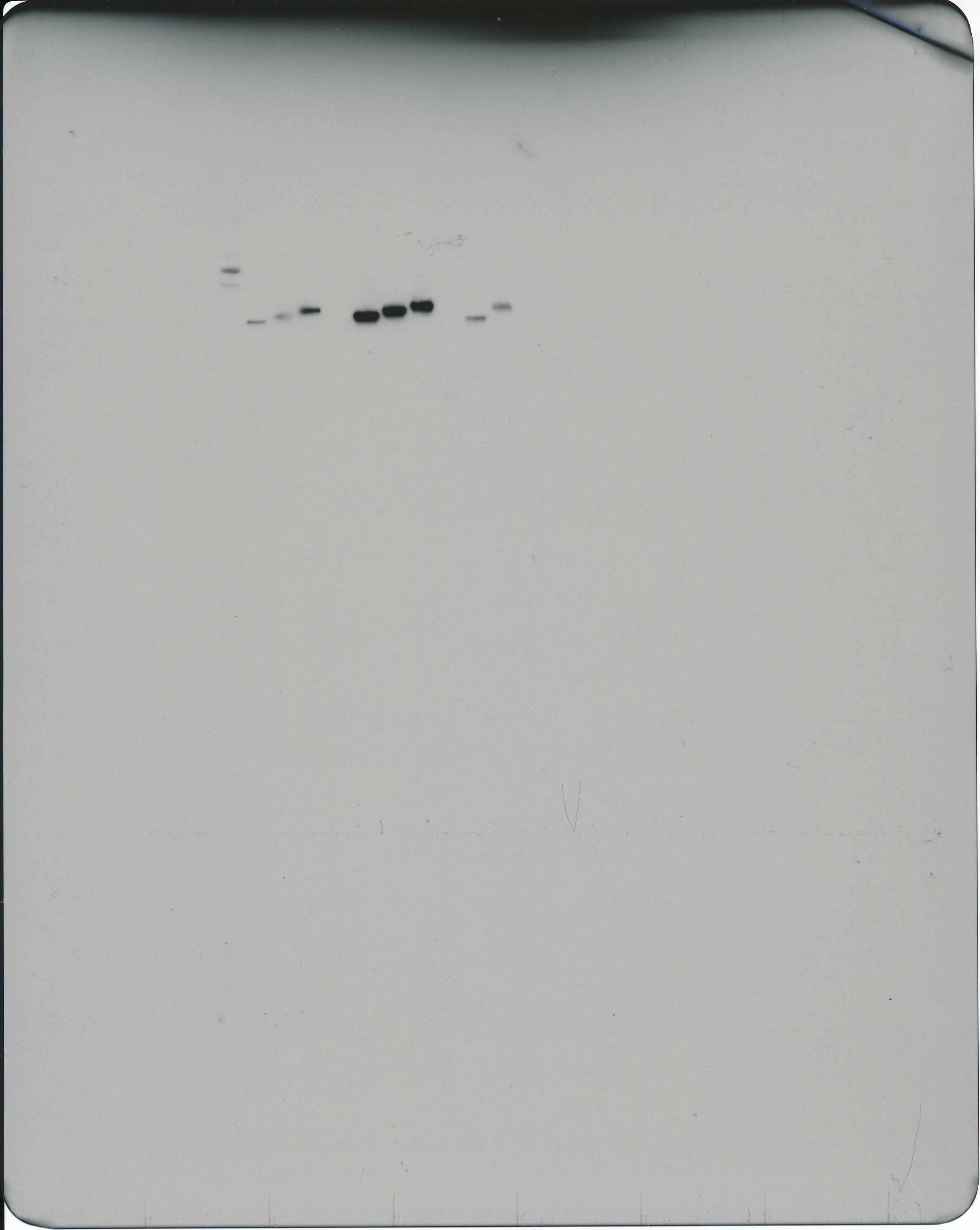

Supplement: Figure 3—source data 1. [file elife-74326-fig3-data1.zip › Figure 3í¬source data/Figure 3B_raw_3.tif]

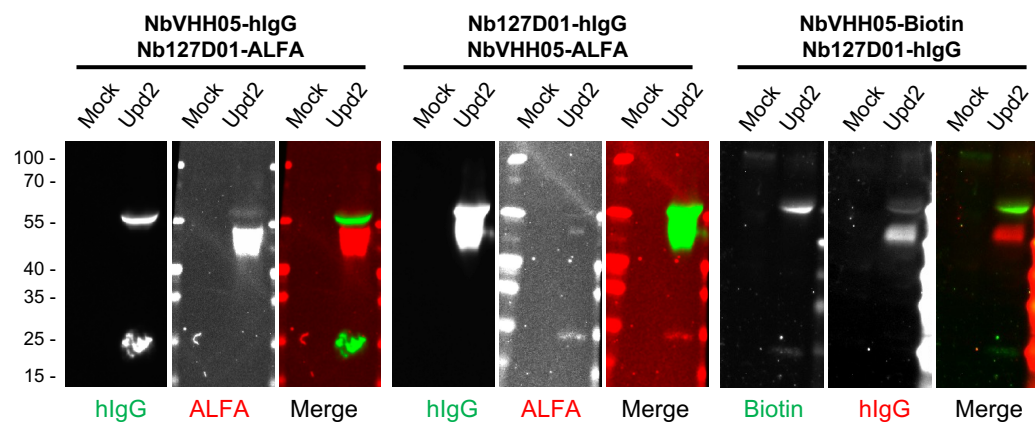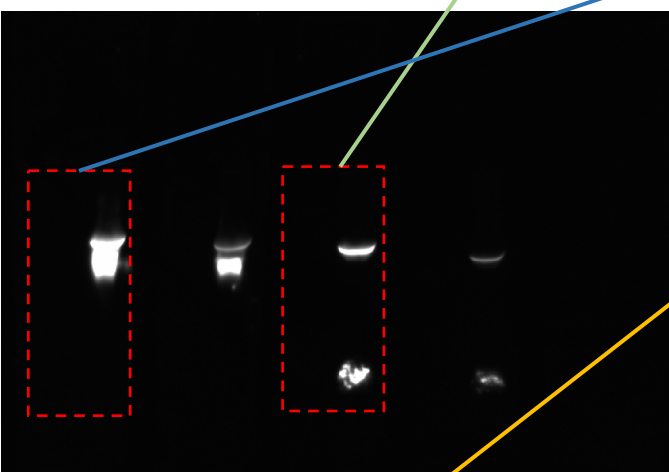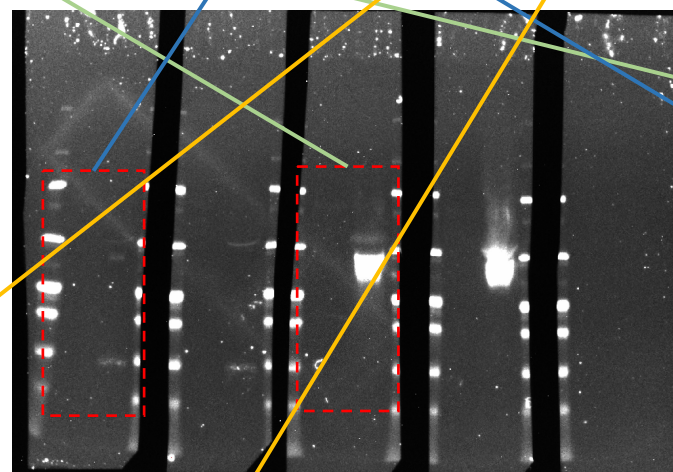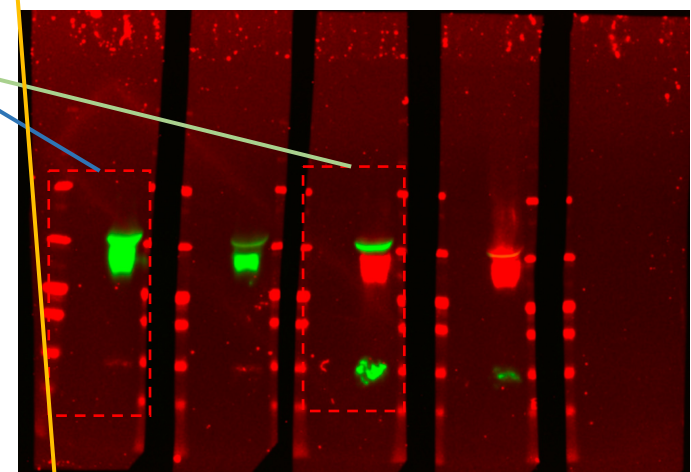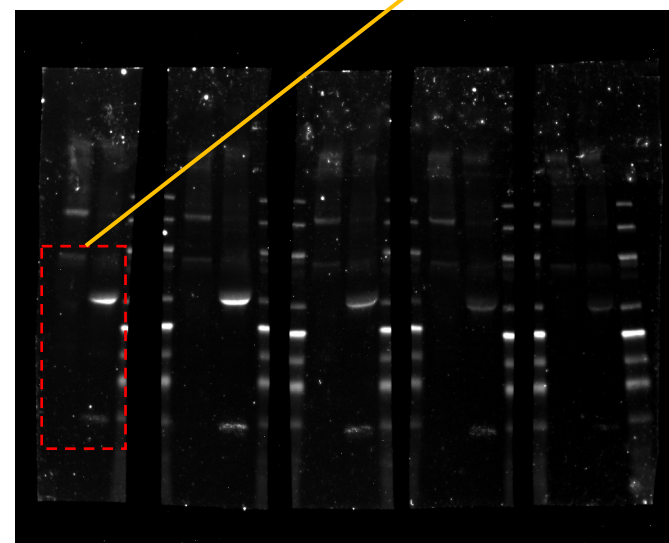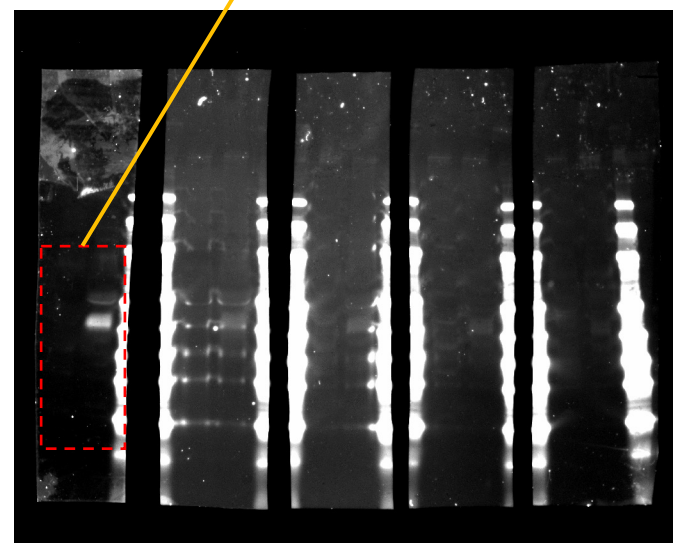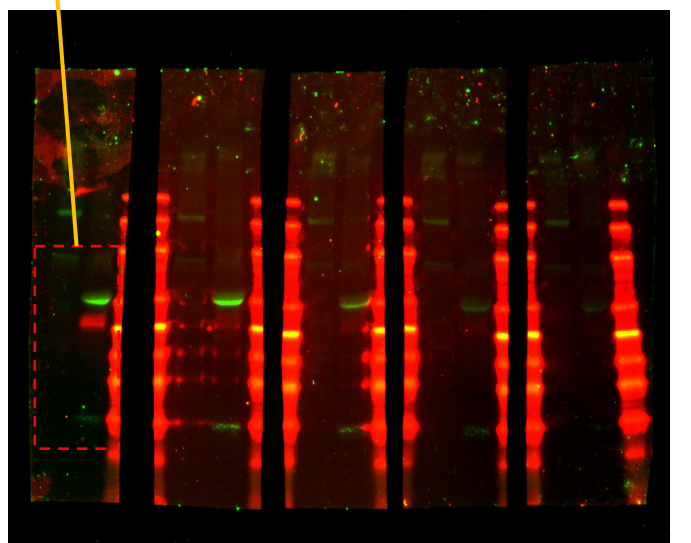

Supplement: Figure 3—source data 1. [file elife-74326-fig3-data1.zip › Figure 3í¬source data/Figure 3C_crop.pdf]

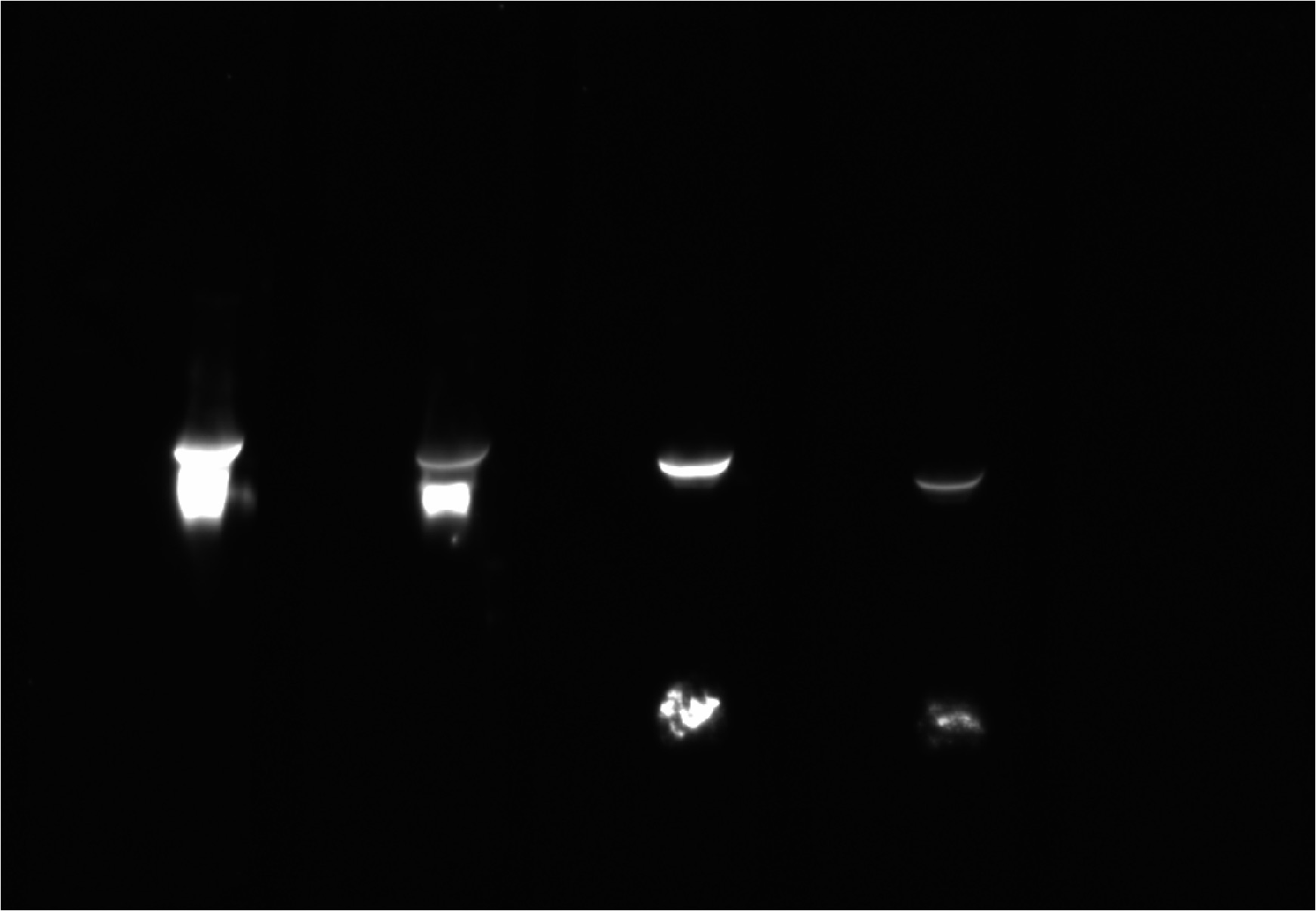

Supplement: Figure 3—source data 1. [file elife-74326-fig3-data1.zip › Figure 3í¬source data/Figure 3C_raw_1.jpg]

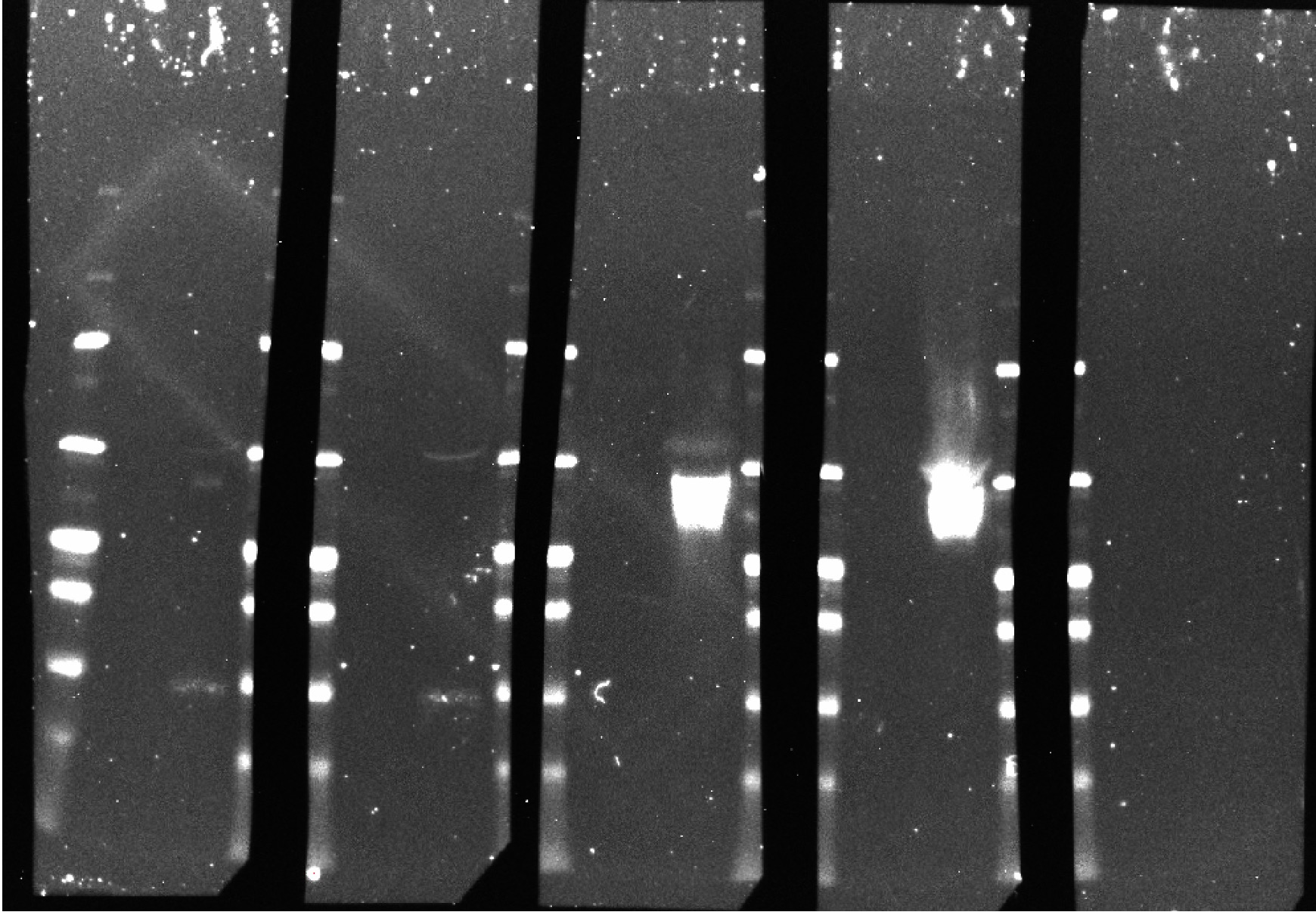

Supplement: Figure 3—source data 1. [file elife-74326-fig3-data1.zip › Figure 3í¬source data/Figure 3C_raw_2.jpg]

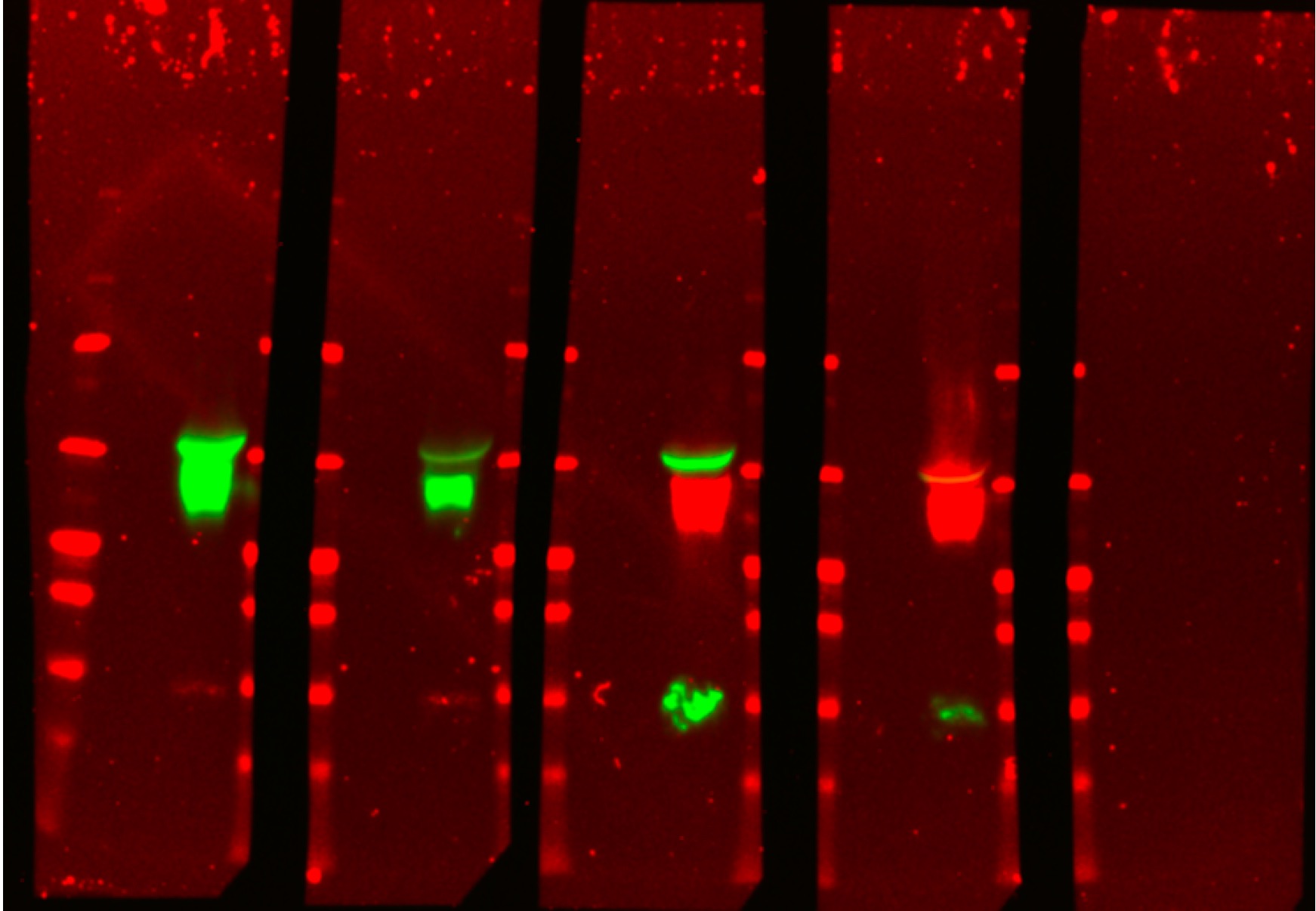

Supplement: Figure 3—source data 1. [file elife-74326-fig3-data1.zip › Figure 3í¬source data/Figure 3C_raw_3 (merged).jpg]

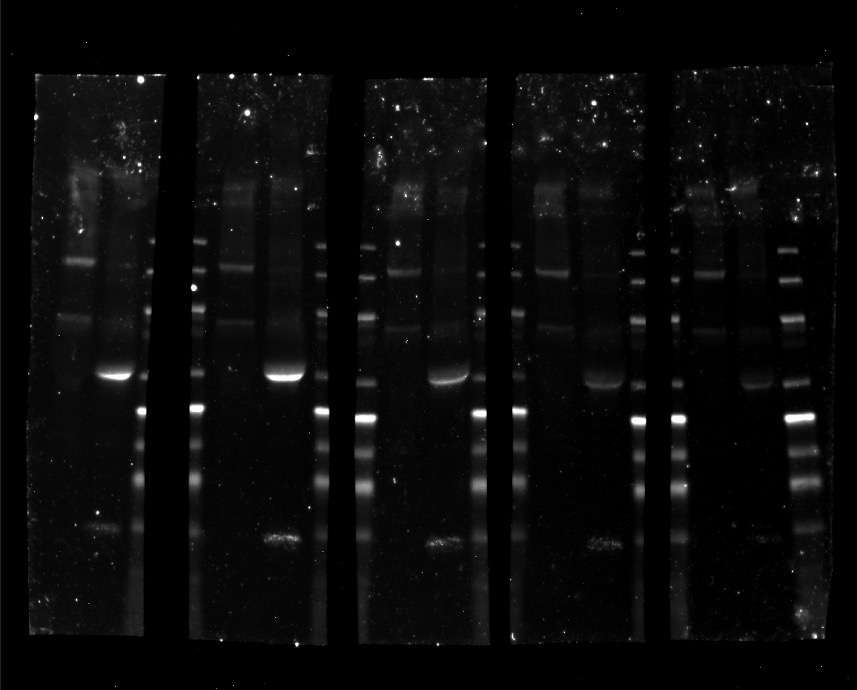

Supplement: Figure 3—source data 1. [file elife-74326-fig3-data1.zip › Figure 3í¬source data/Figure 3C_raw_4.jpg]

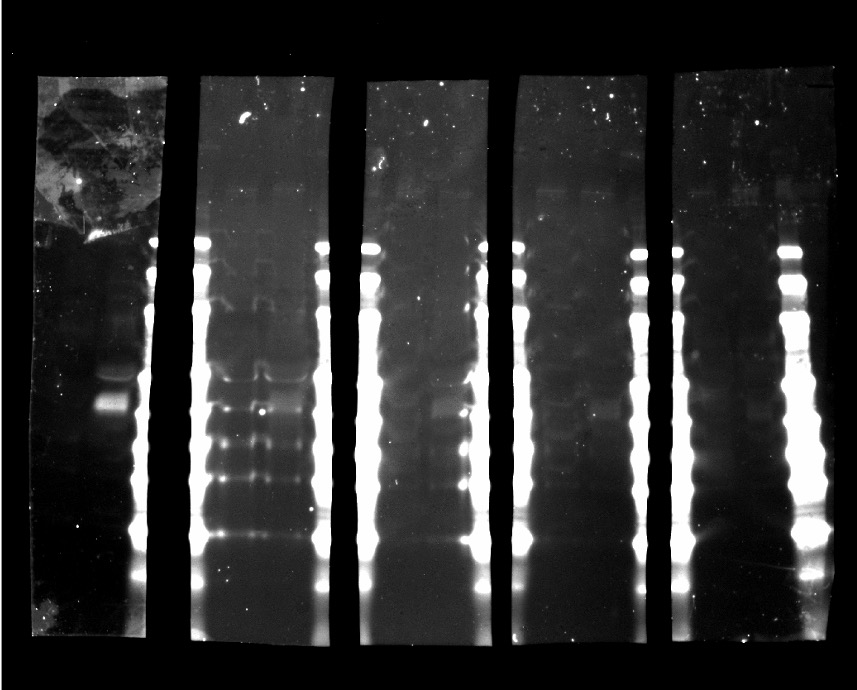

Supplement: Figure 3—source data 1. [file elife-74326-fig3-data1.zip › Figure 3í¬source data/Figure 3C_raw_5.jpg]

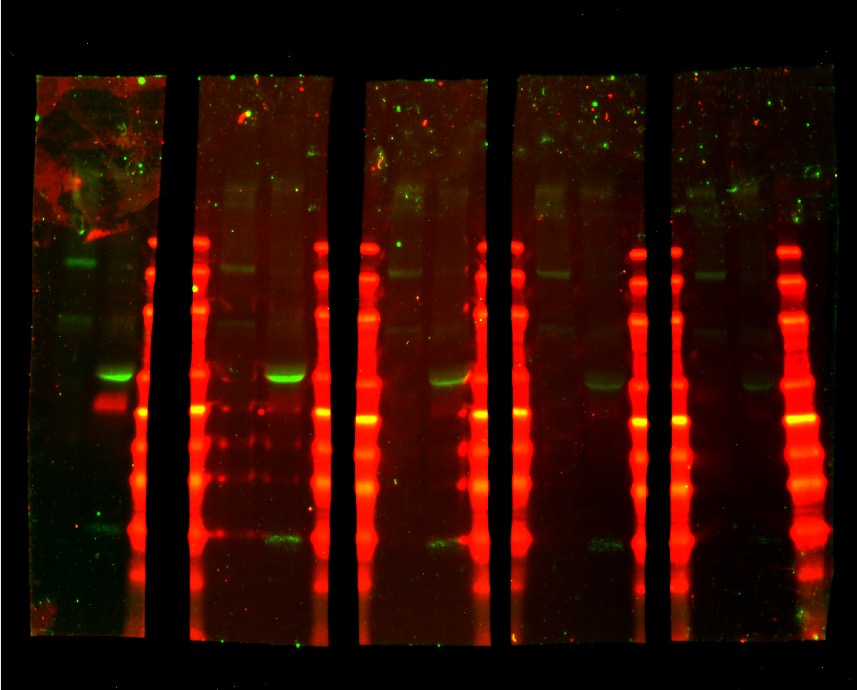

Supplement: Figure 3—source data 1. [file elife-74326-fig3-data1.zip › Figure 3í¬source data/Figure 3C_raw_6 (merged).jpg]

ALFA resin  
IP: ctrl Nb

---

ALFA resin  
IP: NbVHH05

---

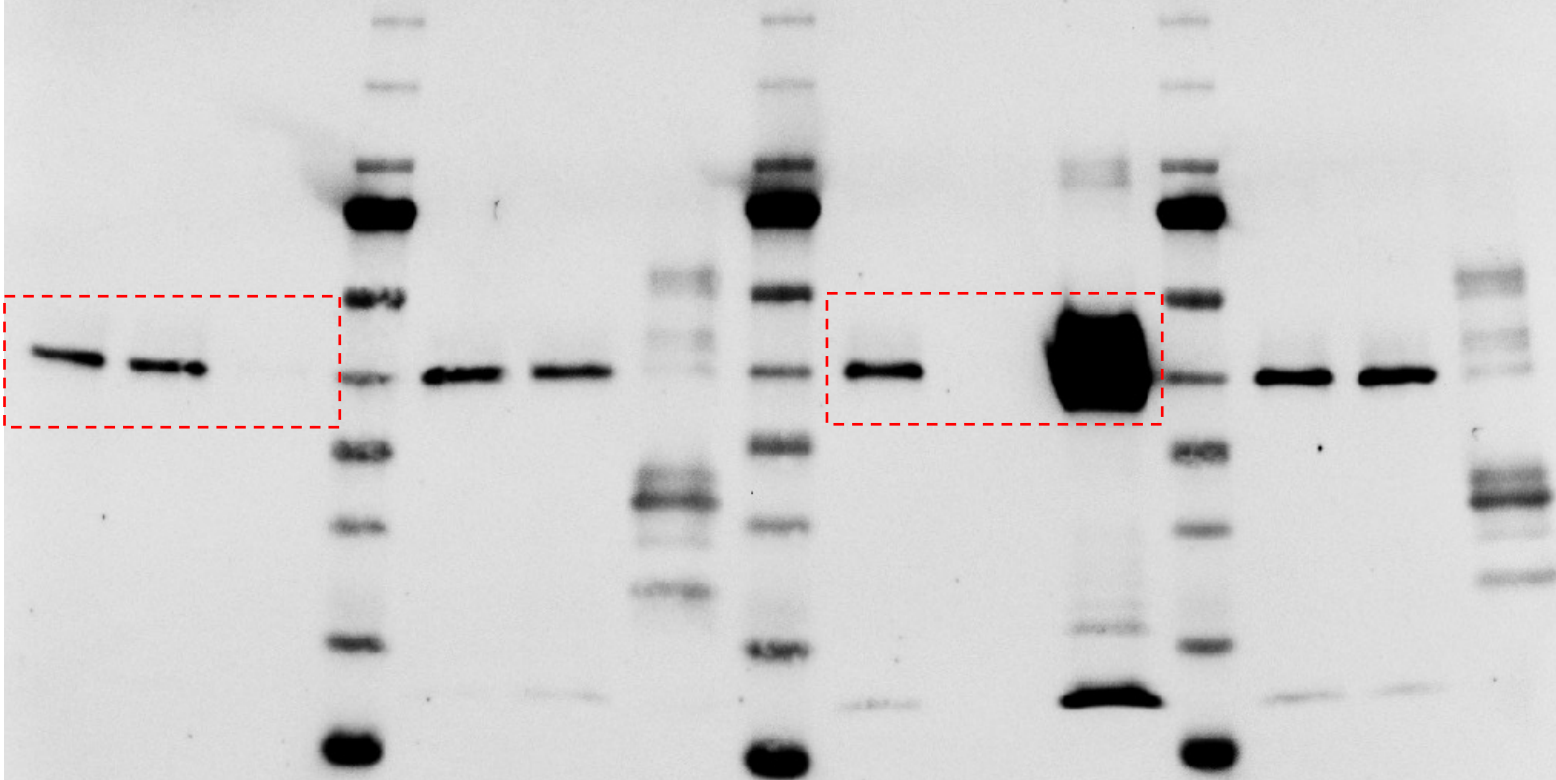

Supplement: Figure 3—source data 1. [file elife-74326-fig3-data1.zip › Figure 3í¬source data/Figure 3D_crop.pdf]

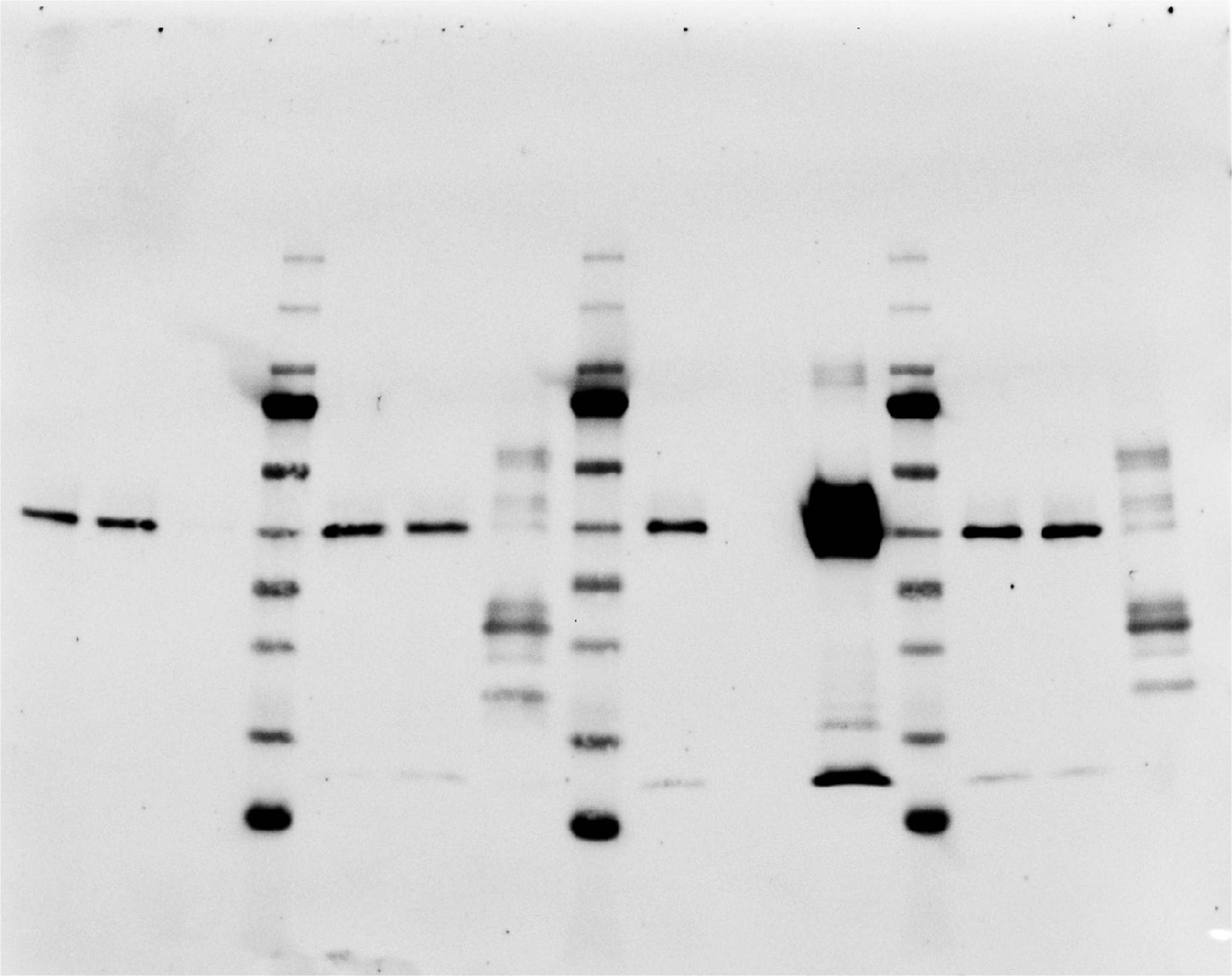

Supplement: Figure 3—source data 1. [file elife-74326-fig3-data1.zip › Figure 3í¬source data/Figure 3D_raw.jpg]

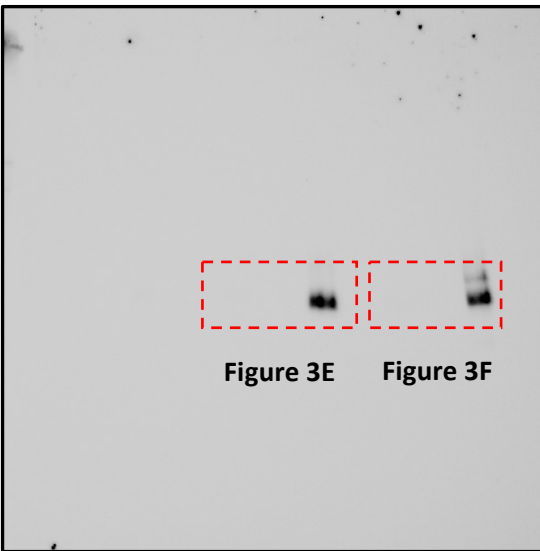

FLAG-GFP-3x127D01-tag  
WB: FLAG

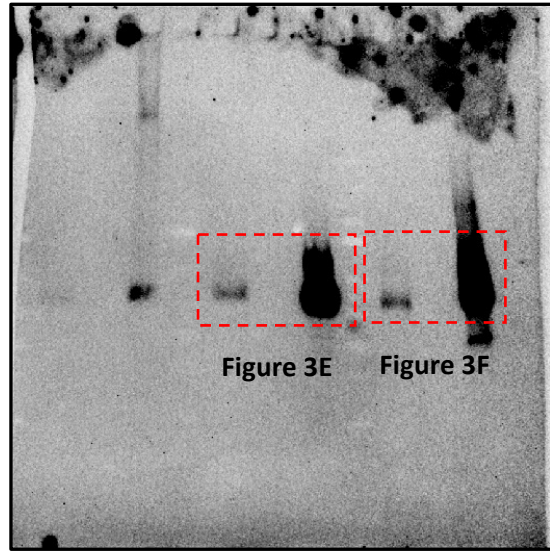

FLAG-GFP-3x127D01-tag  
(long exposure)  
WB: FLAG

Supplement: Figure 3—source data 1. [file elife-74326-fig3-data1.zip › Figure 3í¬source data/Figure 3E&F_crop.pdf]

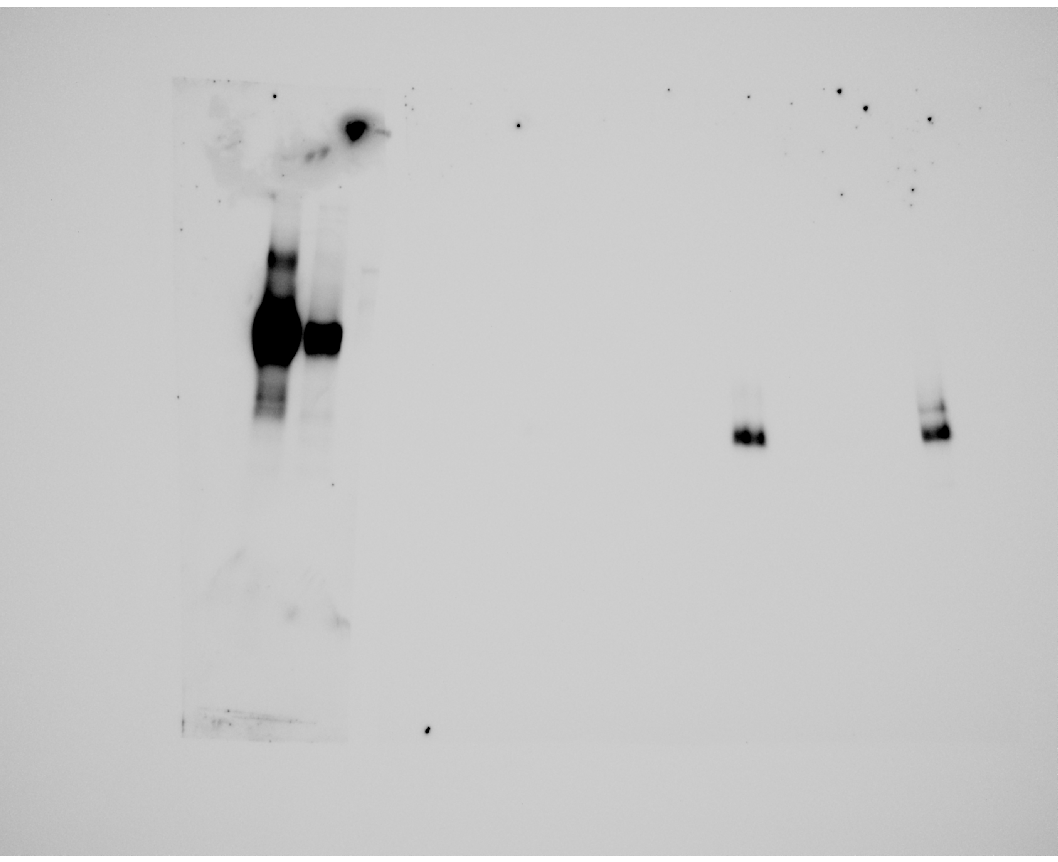

Supplement: Figure 3—source data 1. [file elife-74326-fig3-data1.zip › Figure 3í¬source data/Figure 3E&F_raw.tif]

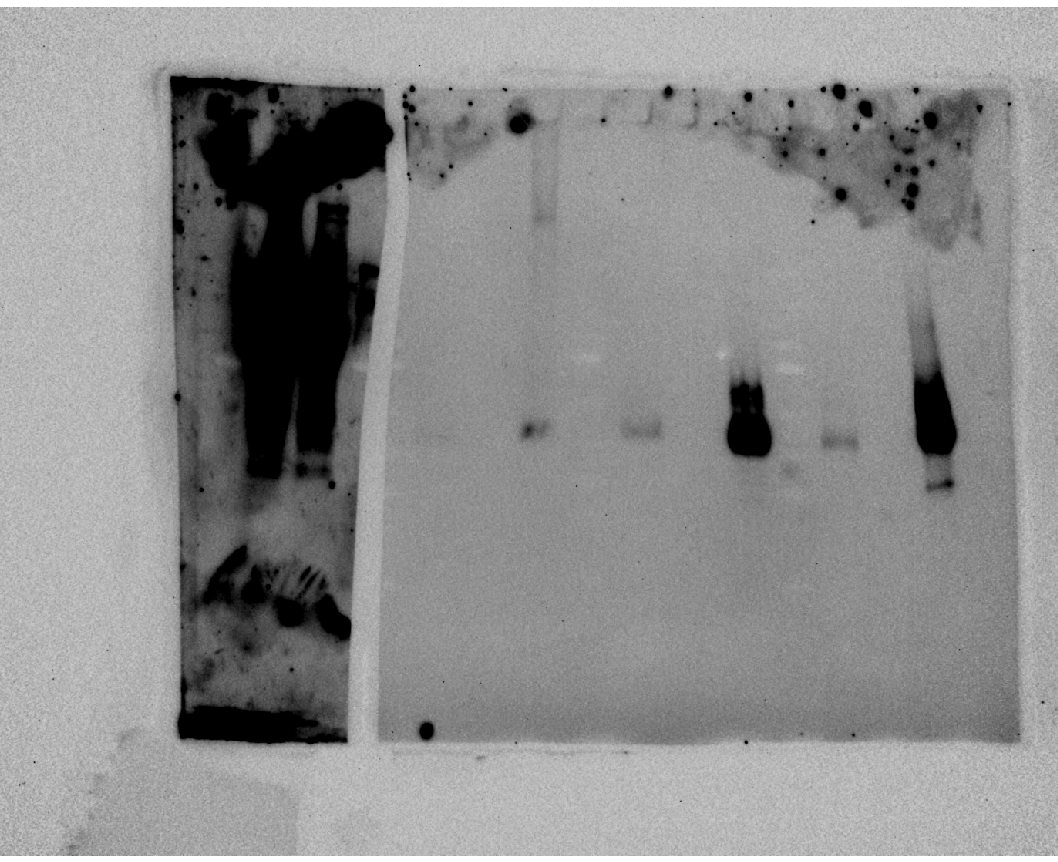

Supplement: Figure 3—source data 1. [file elife-74326-fig3-data1.zip › Figure 3í¬source data/Figure 3E&F_raw_overexposed.tif]

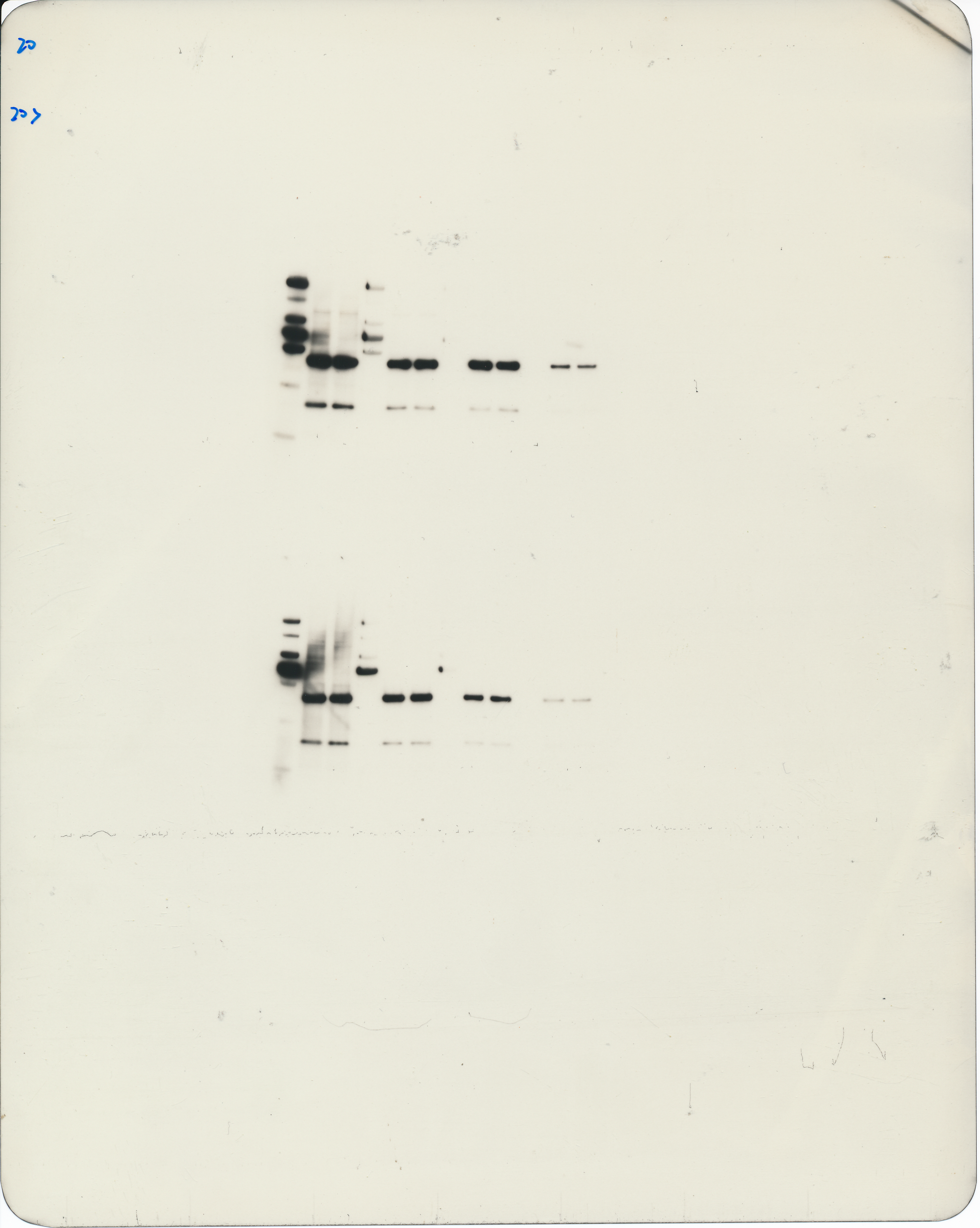

Supplement: Figure 3—figure supplement 1—source data 1. [file elife-74326-fig3-figsupp1-data1.zip › Figure 3í¬figure supplement 1-source data/Figure 3í¬figure supplement 1-raw1.tif]

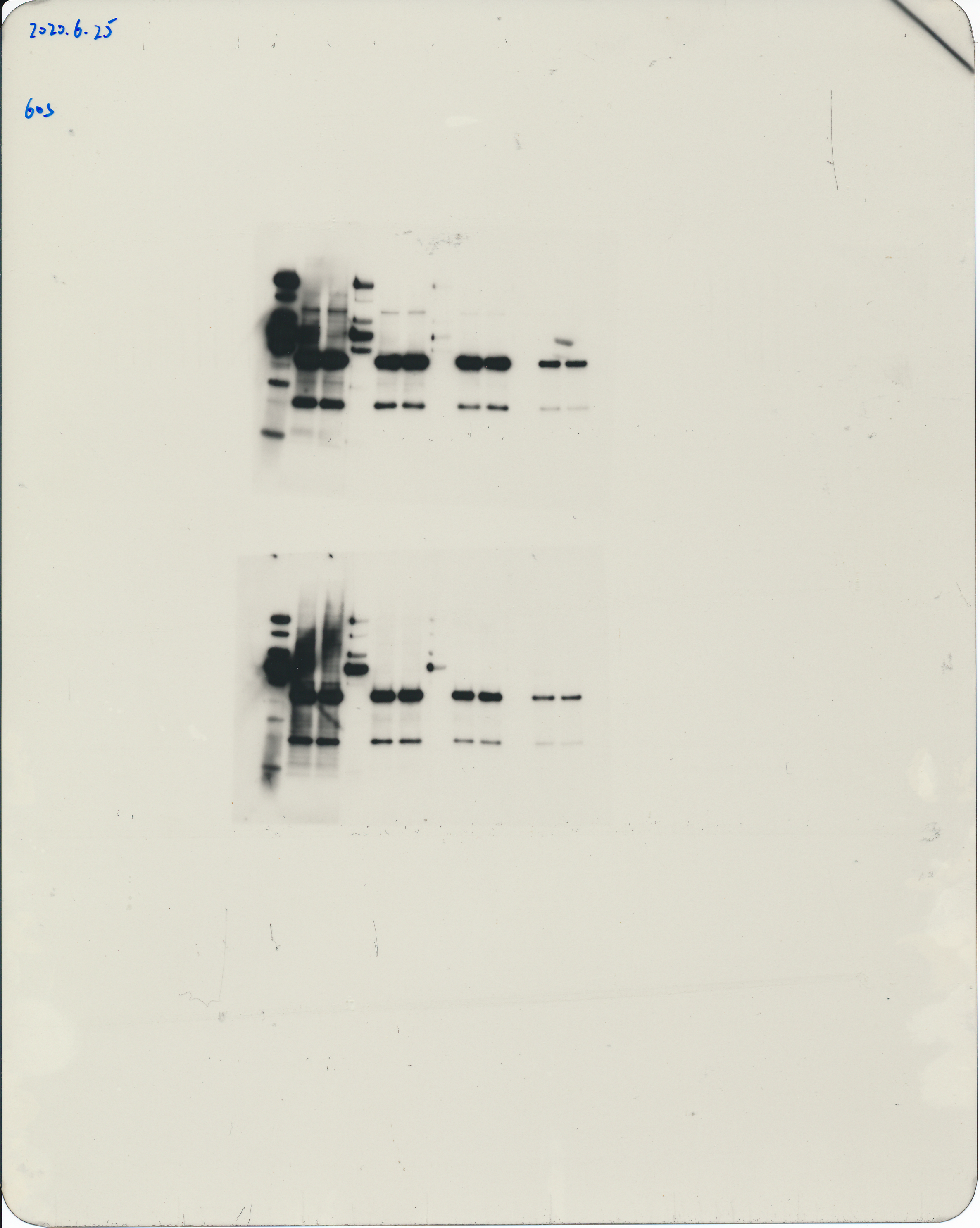

Supplement: Figure 3—figure supplement 1—source data 1. [file elife-74326-fig3-figsupp1-data1.zip › Figure 3í¬figure supplement 1-source data/Figure 3í¬figure supplement 1-raw2.tif]

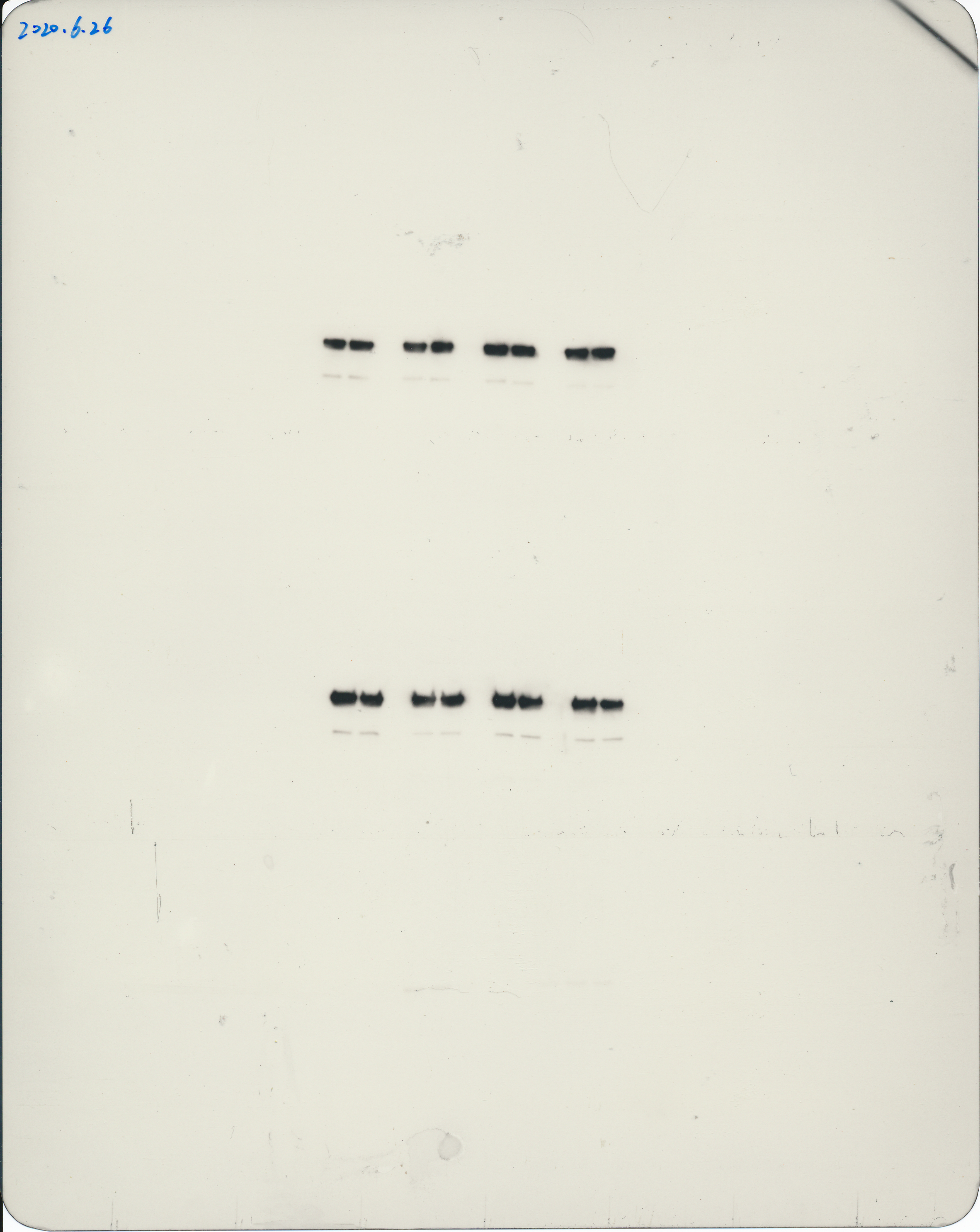

Supplement: Figure 3—figure supplement 1—source data 1. [file elife-74326-fig3-figsupp1-data1.zip › Figure 3í¬figure supplement 1-source data/Figure 3í¬figure supplement 1-raw3.tif]

20

20

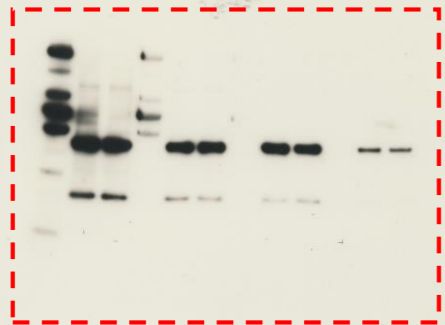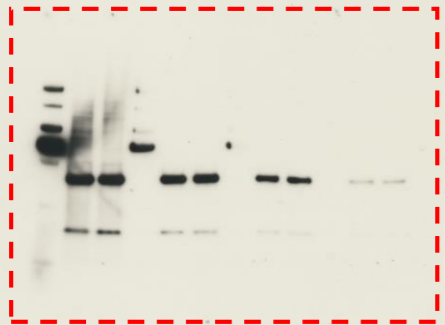

2020.6.25

605

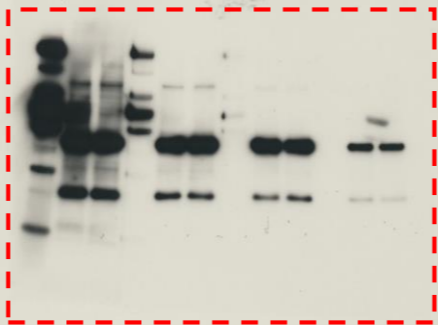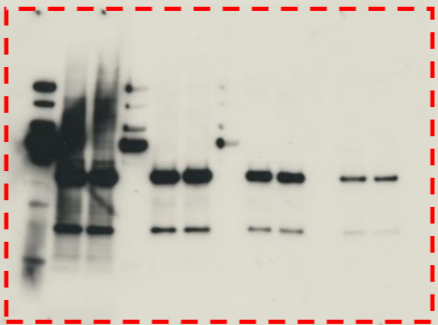

2020.6.26

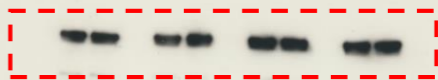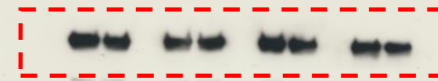

Supplement: Figure 3—figure supplement 1—source data 1. [file elife-74326-fig3-figsupp1-data1.zip › Figure 3í¬figure supplement 1-source data/Figure 3í¬figure supplement 1_Crop.pdf]

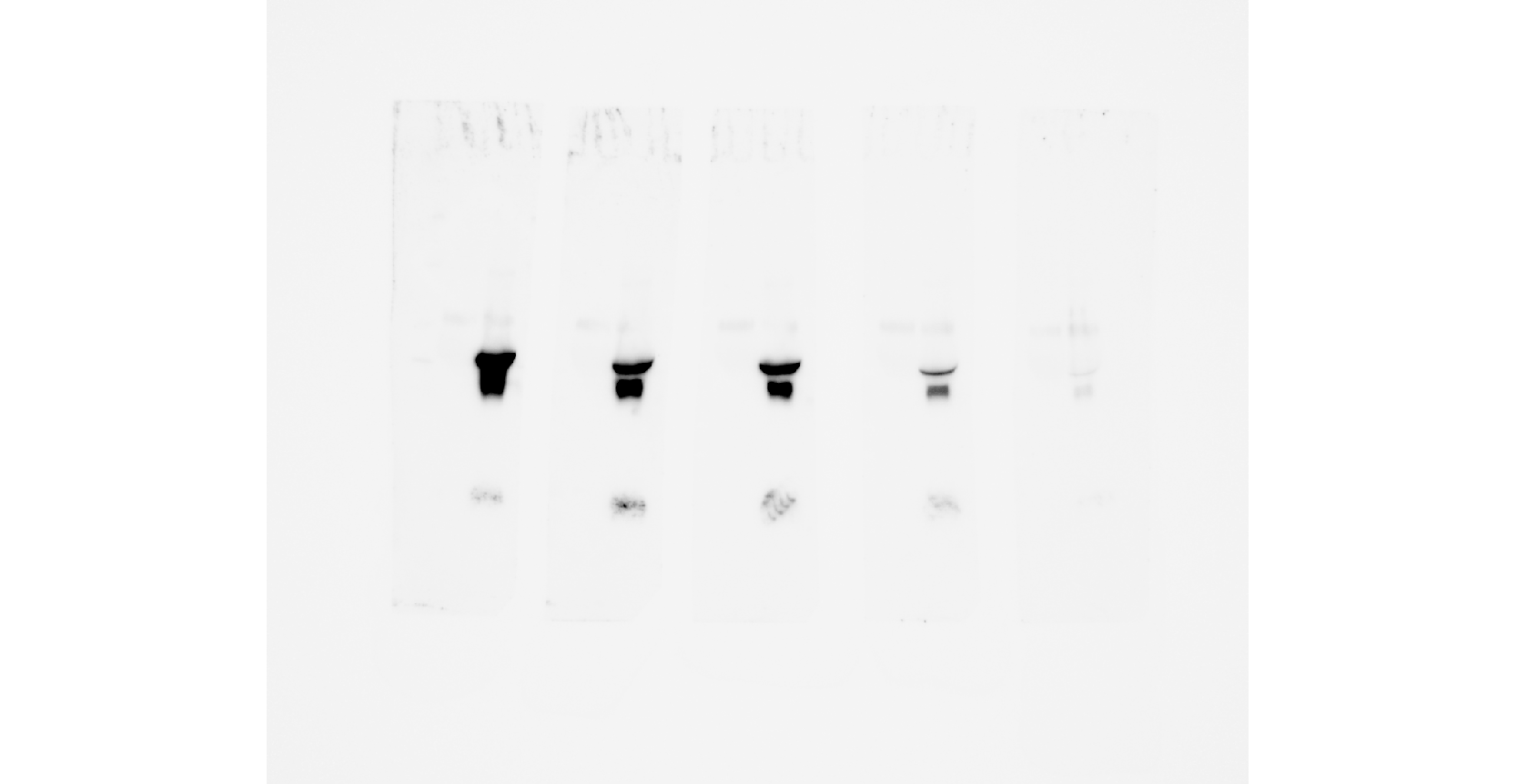

Supplement: Figure 3—figure supplement 2—source data 1. [file elife-74326-fig3-figsupp2-data1.zip › Figure 3í¬figure supplement 2B and 2C-source data/Figure 3í¬figure supplement 2-B_16s.tif]

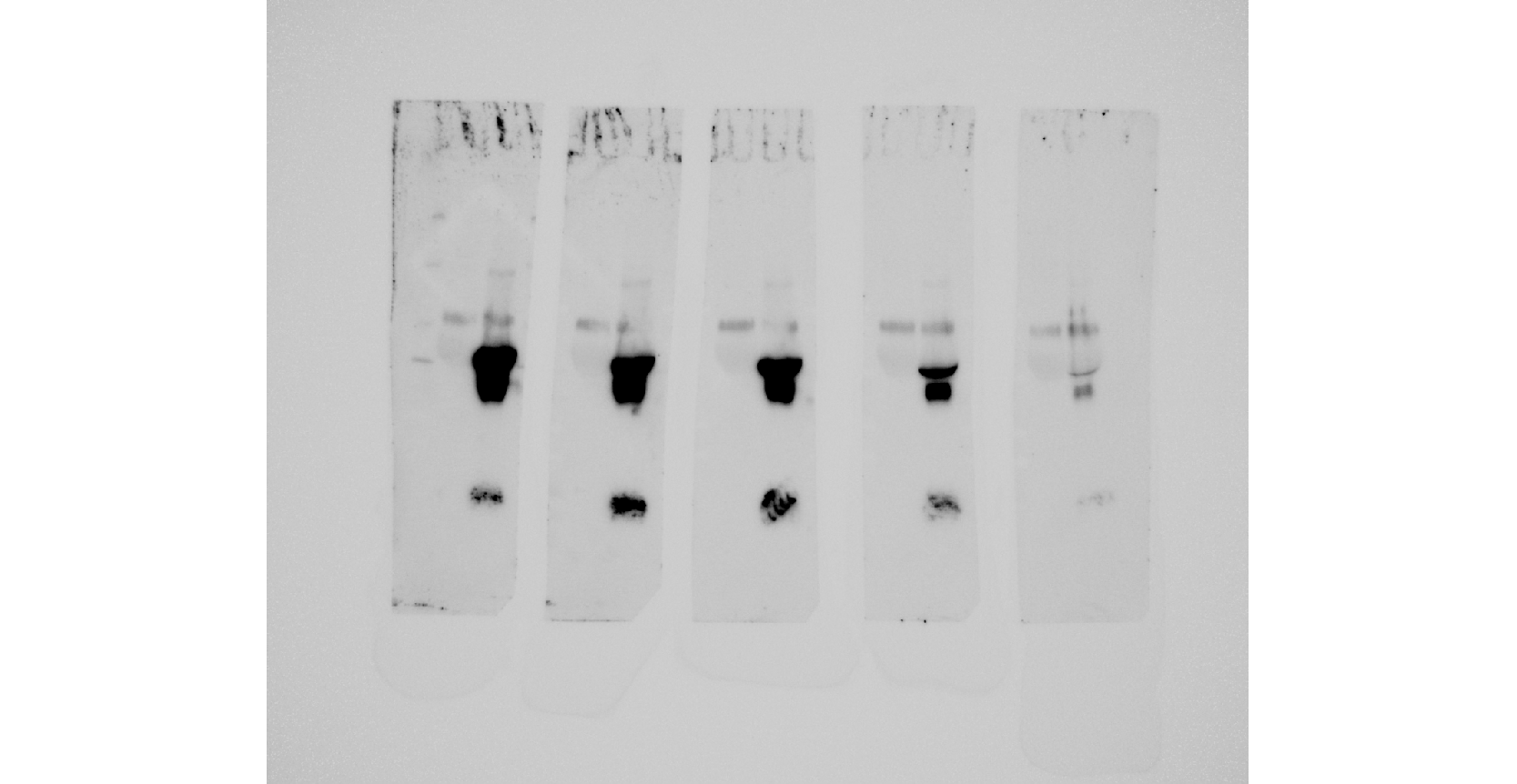

Supplement: Figure 3—figure supplement 2—source data 1. [file elife-74326-fig3-figsupp2-data1.zip › Figure 3í¬figure supplement 2B and 2C-source data/Figure 3í¬figure supplement 2-B_26s.tif]

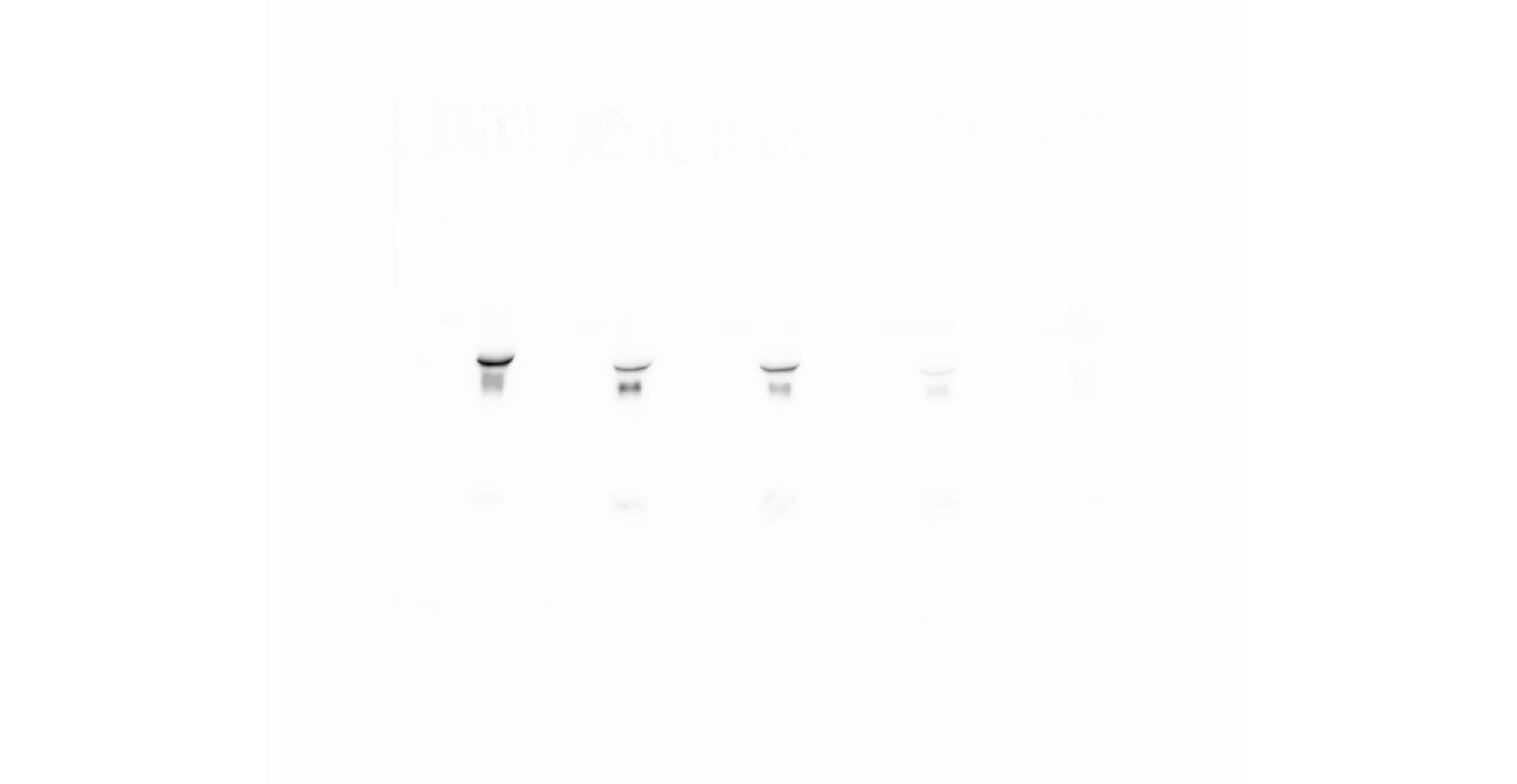

Supplement: Figure 3—figure supplement 2—source data 1. [file elife-74326-fig3-figsupp2-data1.zip › Figure 3í¬figure supplement 2B and 2C-source data/Figure 3í¬figure supplement 2-B_6s.tif]

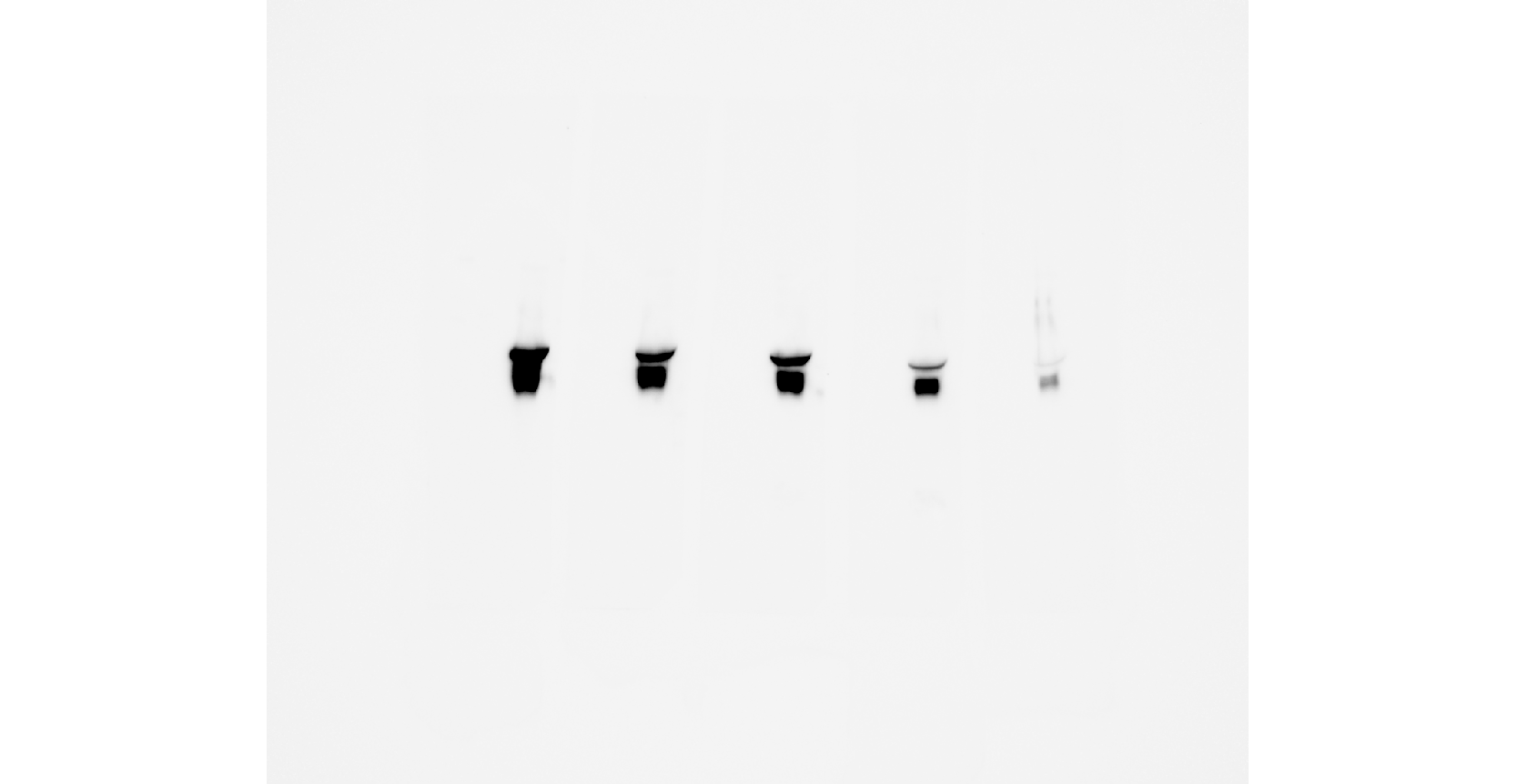

Supplement: Figure 3—figure supplement 2—source data 1. [file elife-74326-fig3-figsupp2-data1.zip › Figure 3í¬figure supplement 2B and 2C-source data/Figure 3í¬figure supplement 2-C_16s.tif]

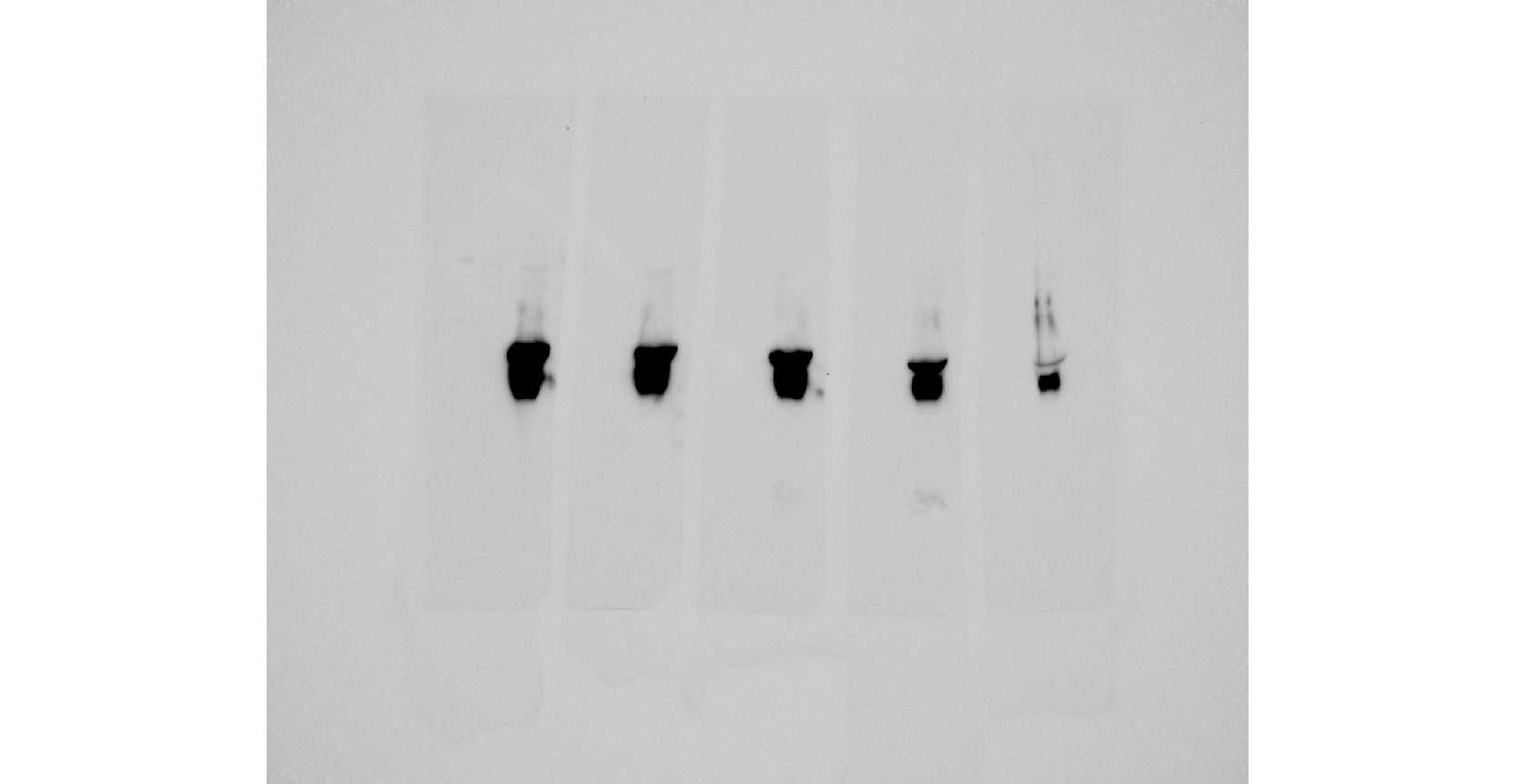

Supplement: Figure 3—figure supplement 2—source data 1. [file elife-74326-fig3-figsupp2-data1.zip › Figure 3í¬figure supplement 2B and 2C-source data/Figure 3í¬figure supplement 2-C_26s.tif]

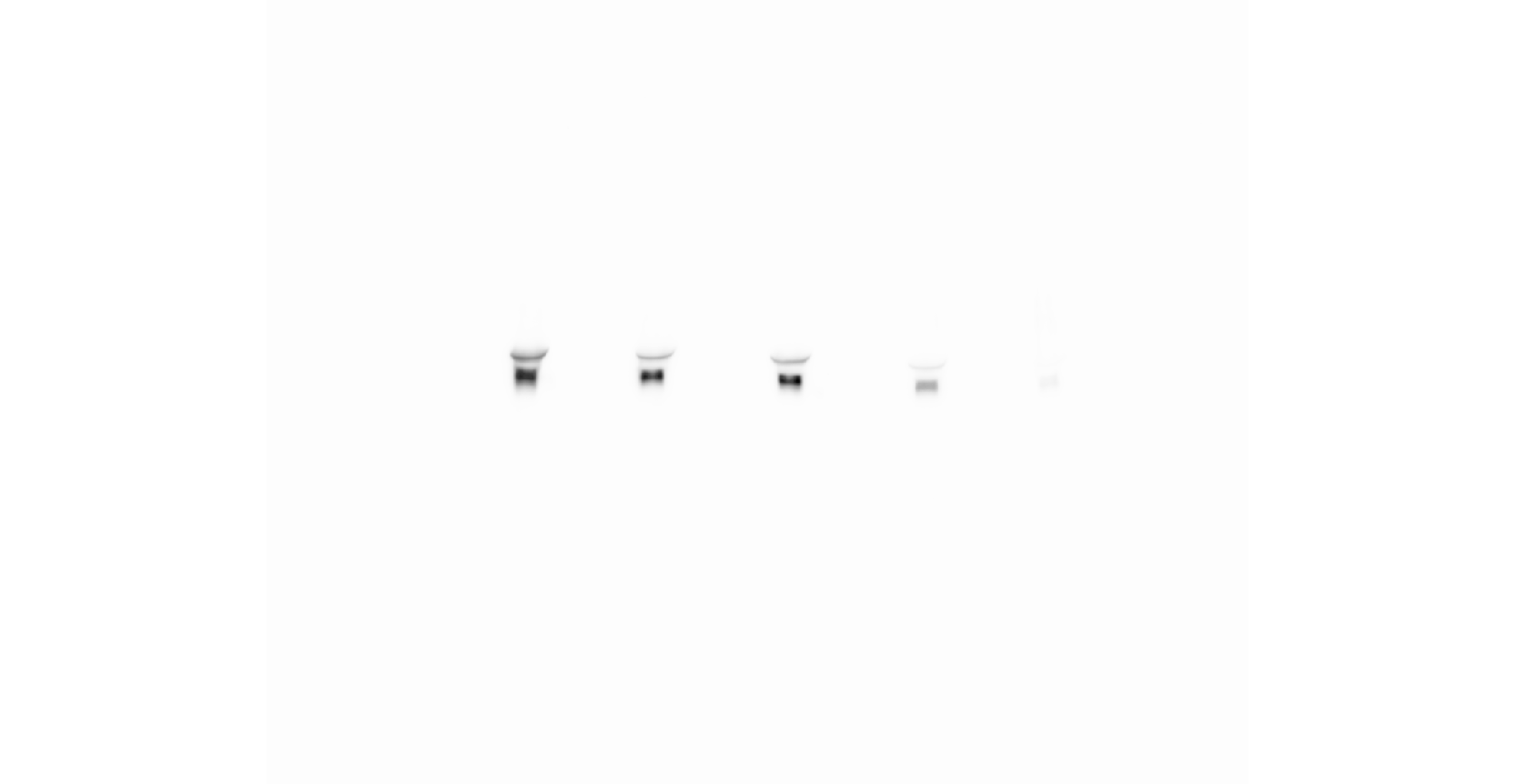

Supplement: Figure 3—figure supplement 2—source data 1. [file elife-74326-fig3-figsupp2-data1.zip › Figure 3í¬figure supplement 2B and 2C-source data/Figure 3í¬figure supplement 2-C_6s.tif]

2020.7.29 Anti-GFP  
Western blot  
1. Bip-GFP-WH1  
2. Bip-GFP-WH1  
Bip-WH1-WH1  
3. Bip-GFP-WH1  
4. Bip-GFP-WH1  
Bip-WH1-WH1

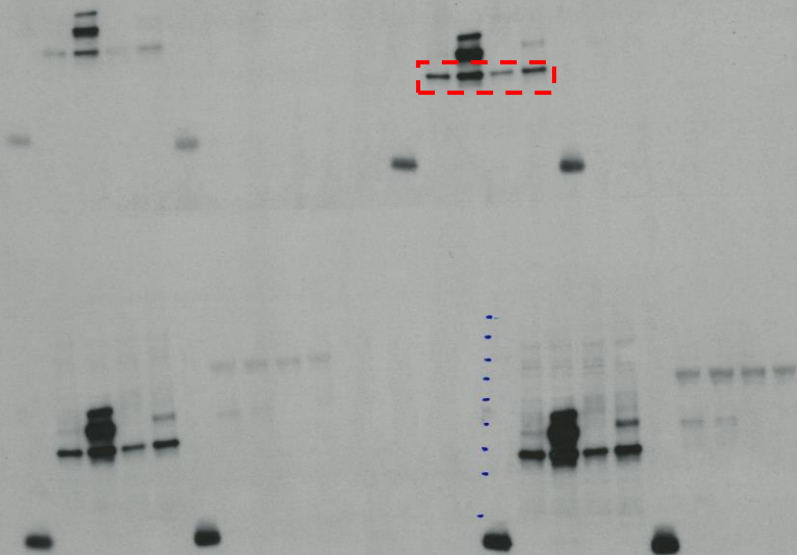

2020.7.29  
Anti-tubulin

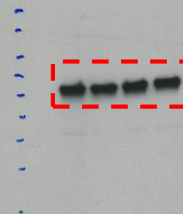

Supplement: Figure 4—source data 1. [file elife-74326-fig4-data1.zip › Figure 4Dí¬source data/Figure 4D_Crop.pdf]

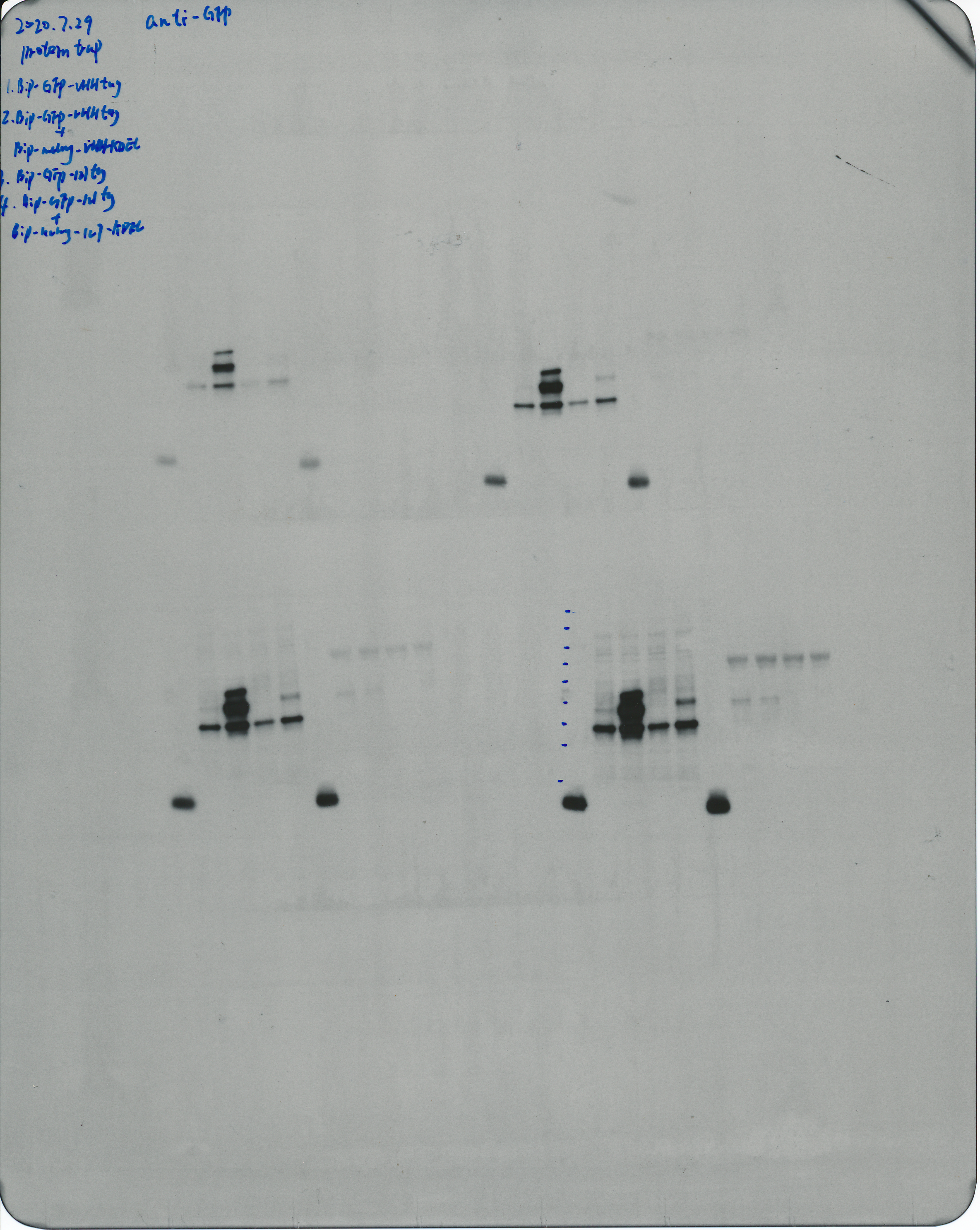

Supplement: Figure 4—source data 1. [file elife-74326-fig4-data1.zip › Figure 4Dí¬source data/Figure 4D_raw_1.tif]

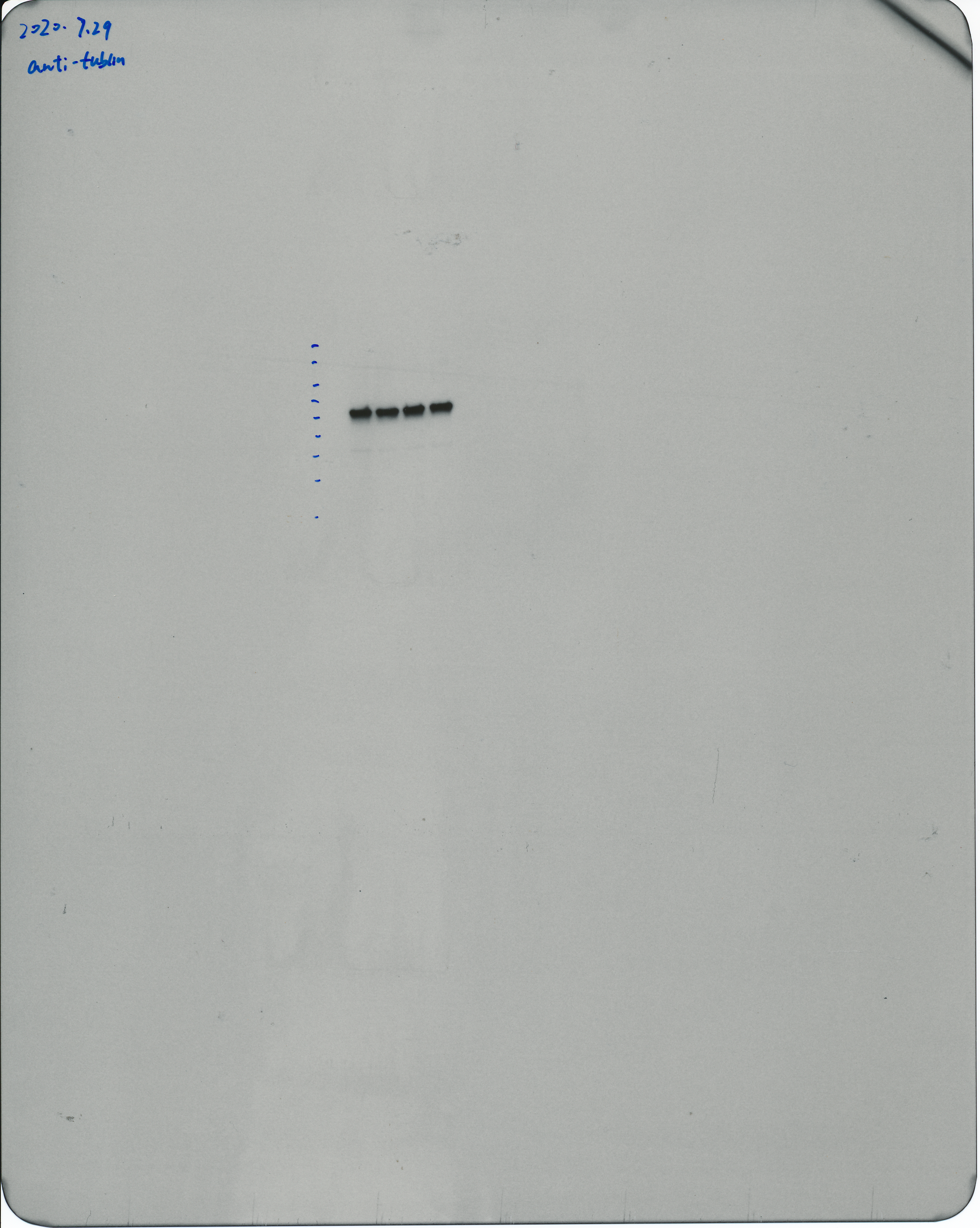

Supplement: Figure 4—source data 1. [file elife-74326-fig4-data1.zip › Figure 4Dí¬source data/Figure 4D_raw_2.tif]

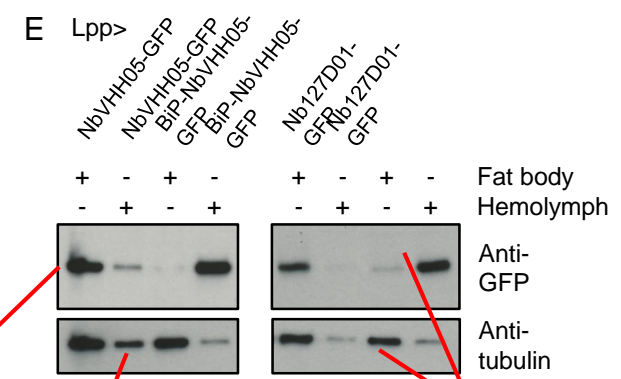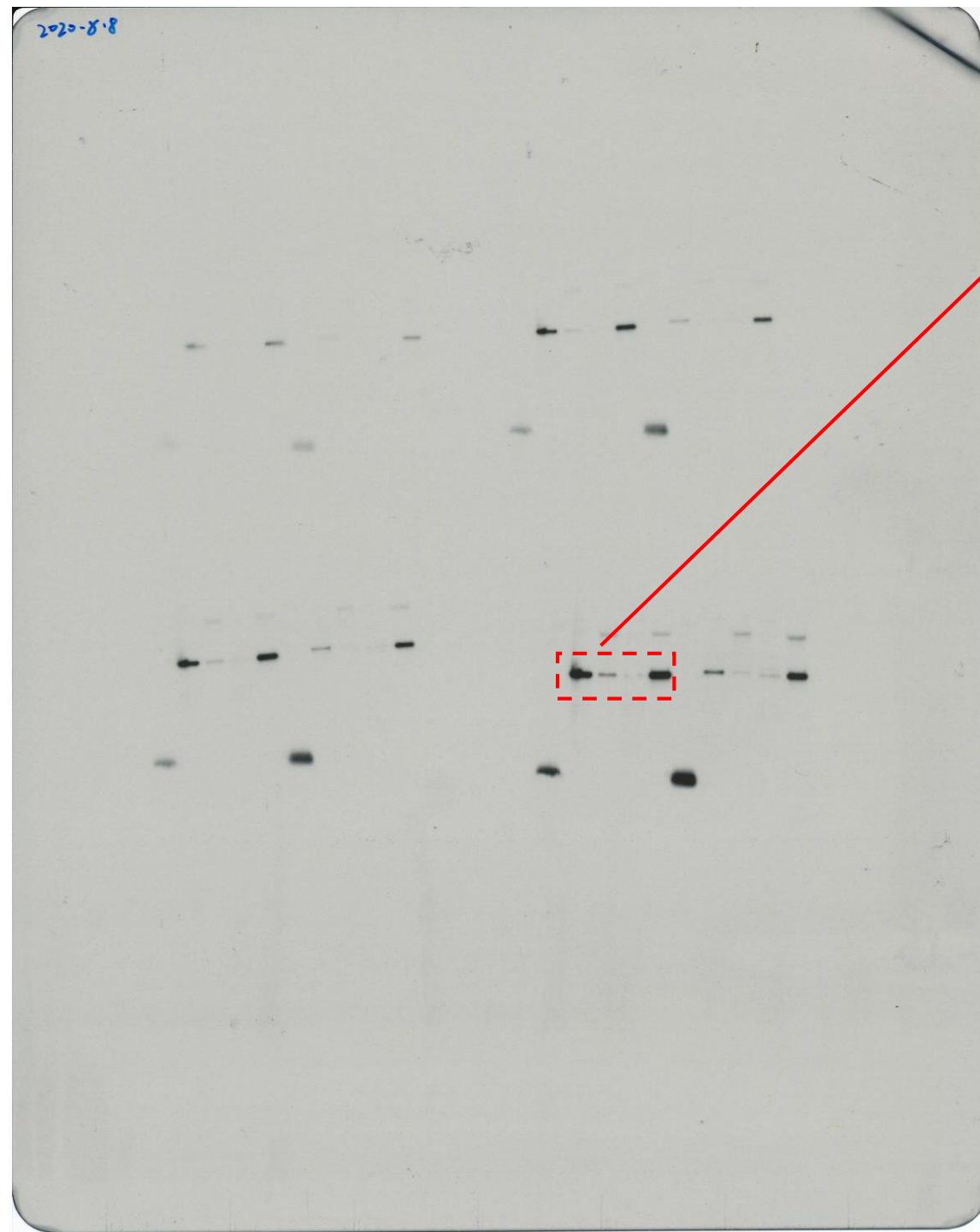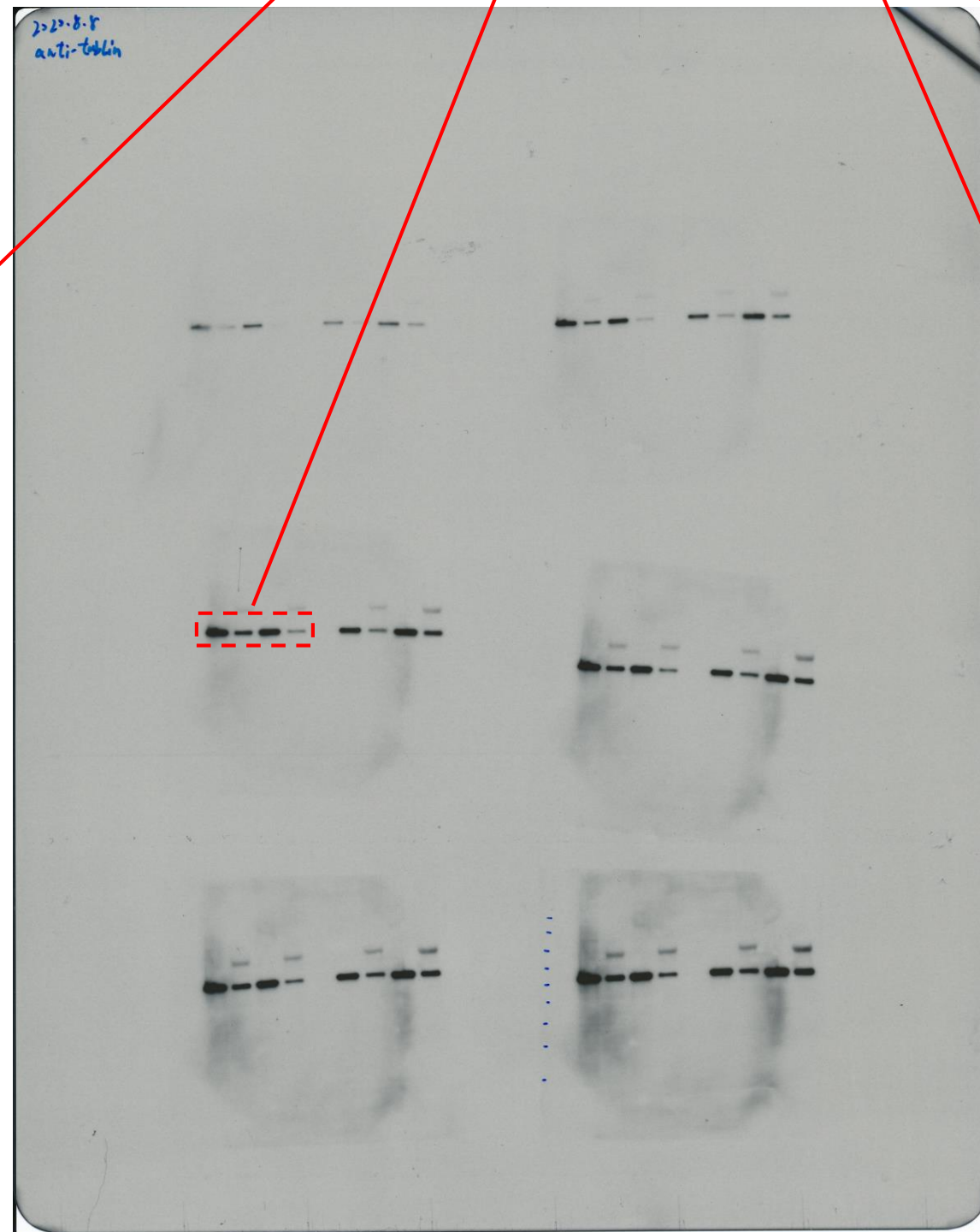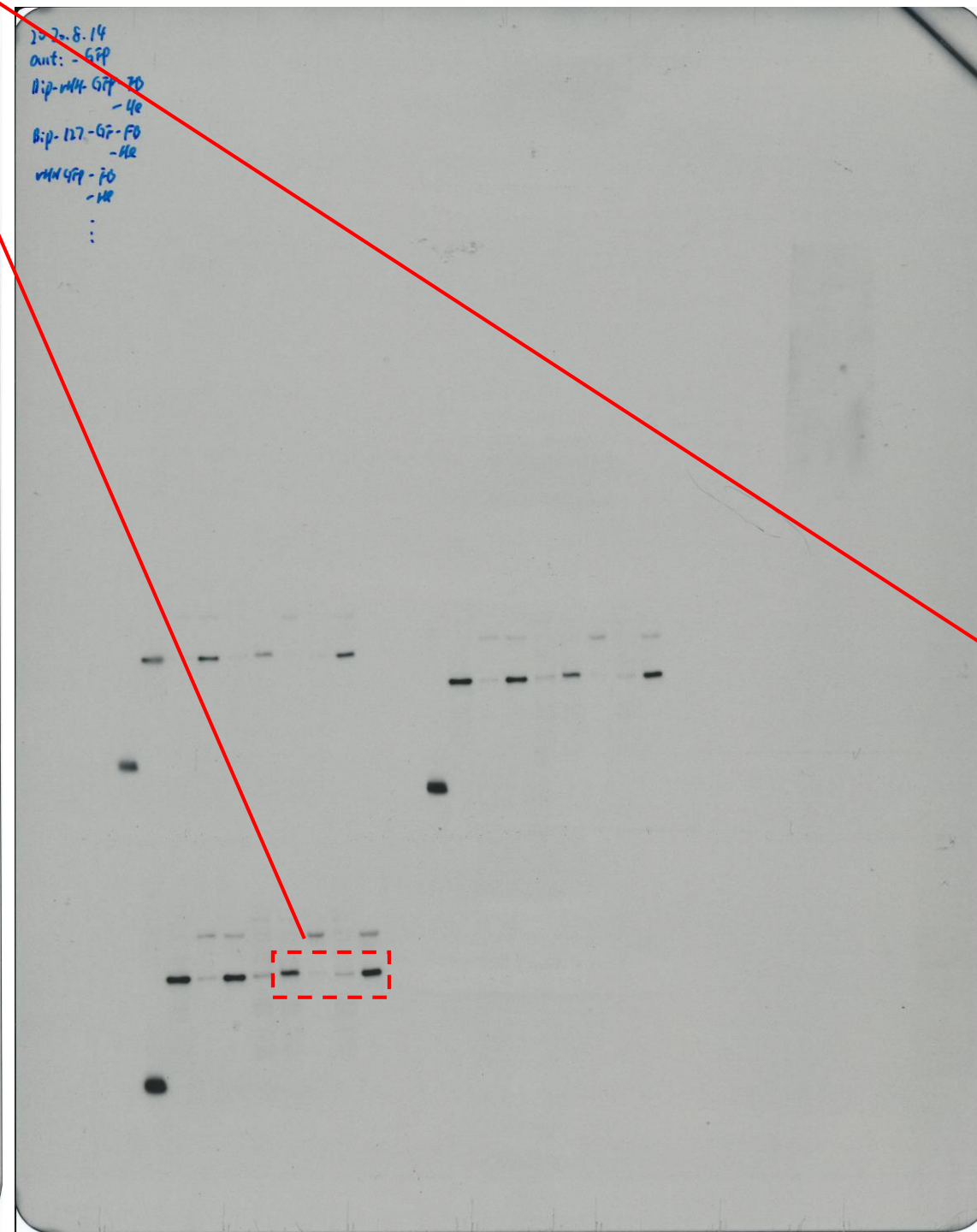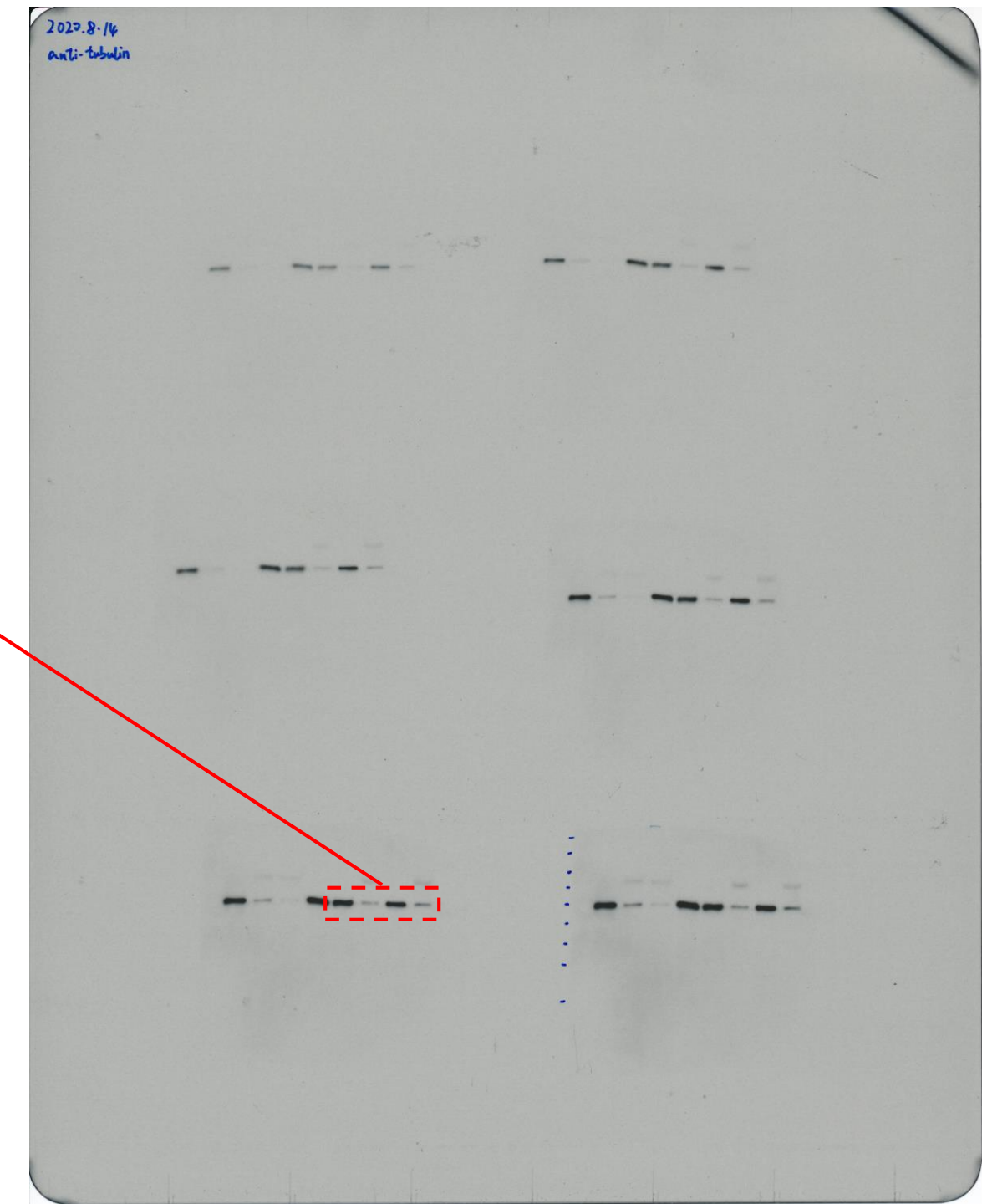

Supplement: Figure 5—source data 1. [file elife-74326-fig5-data1.zip › Figure 5E and 5Fí¬source data/Figure 5E_Crop.pdf]

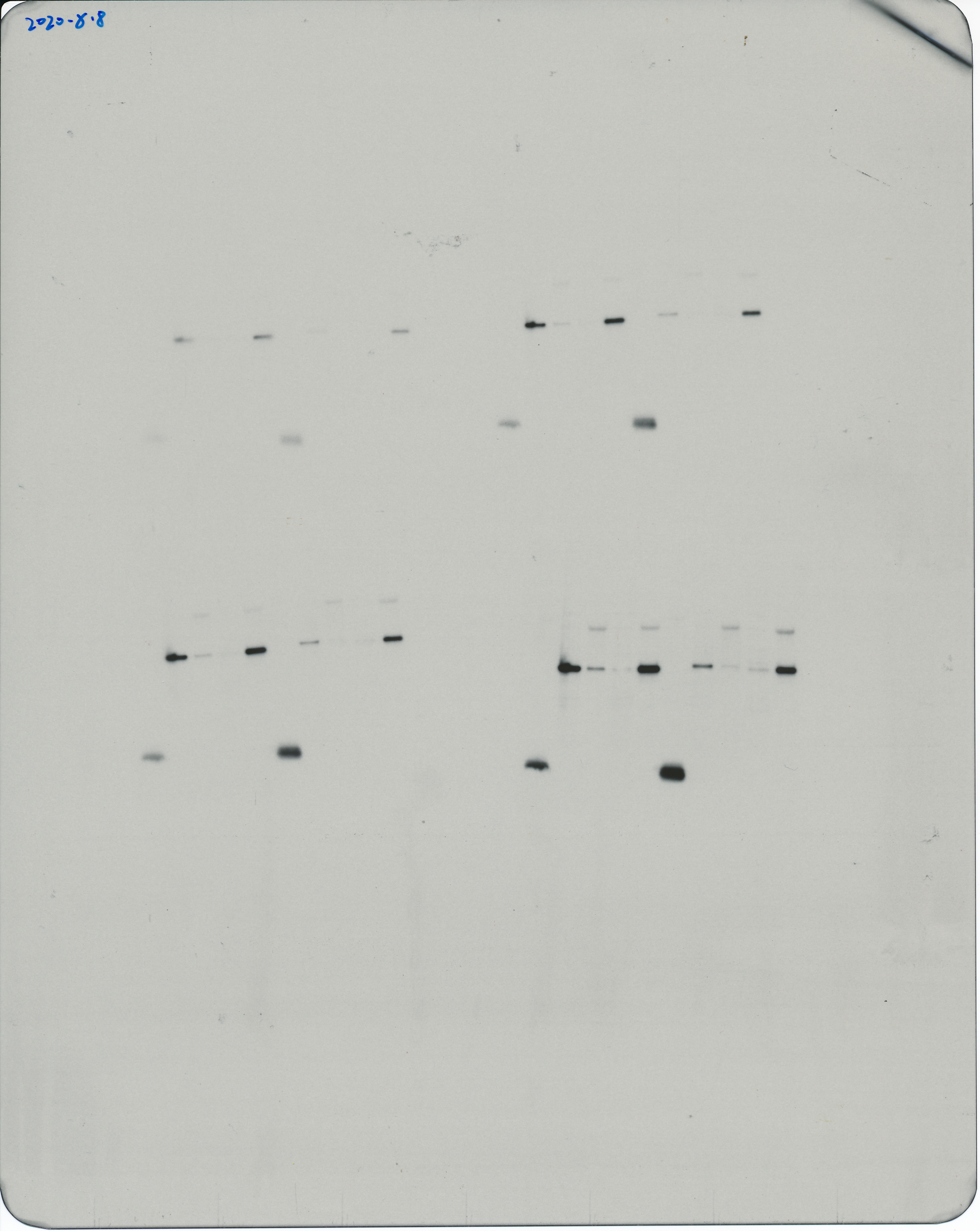

Supplement: Figure 5—source data 1. [file elife-74326-fig5-data1.zip › Figure 5E and 5Fí¬source data/Figure 5E_raw_1.tif]

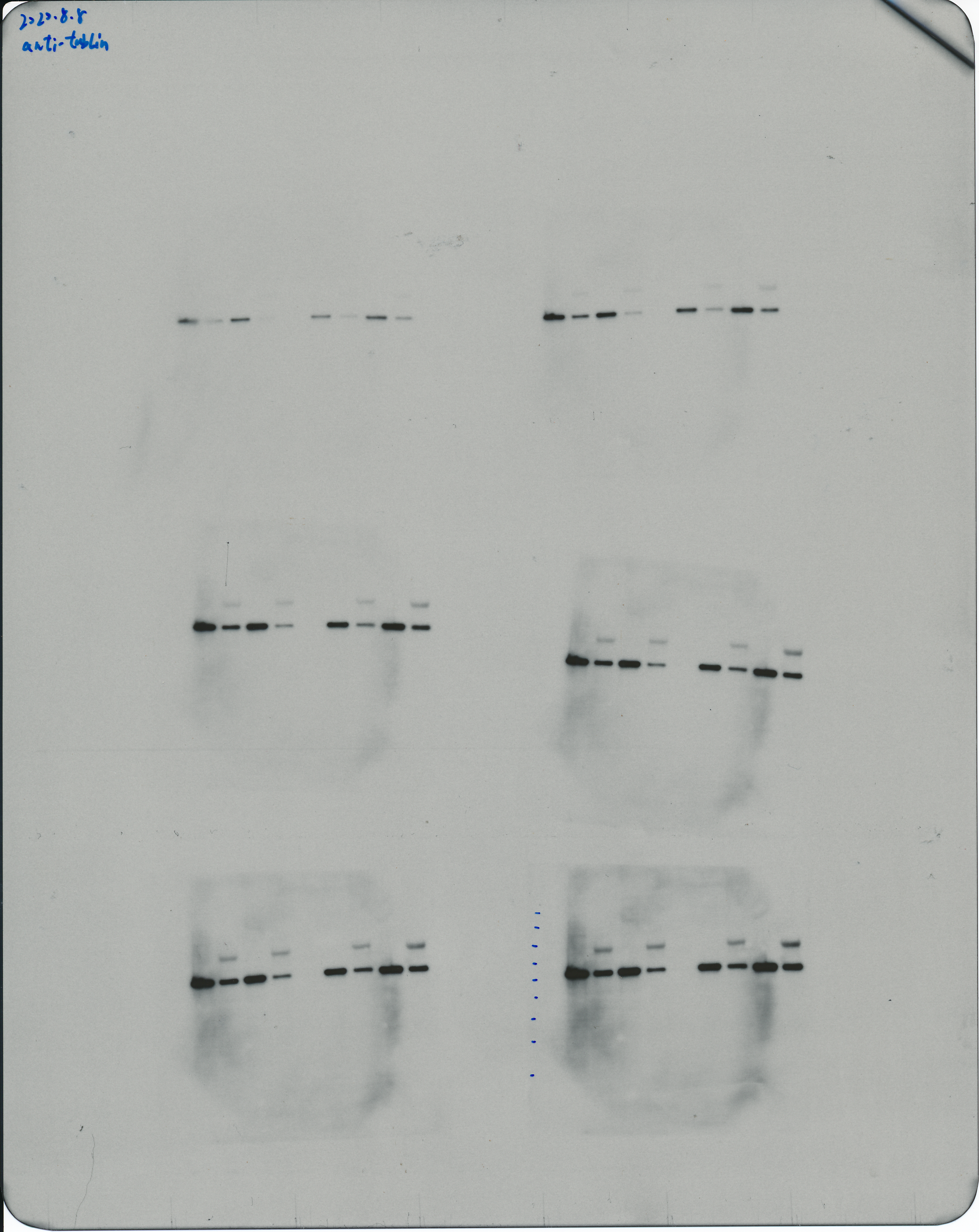

Supplement: Figure 5—source data 1. [file elife-74326-fig5-data1.zip › Figure 5E and 5Fí¬source data/Figure 5E_raw_2.tif]

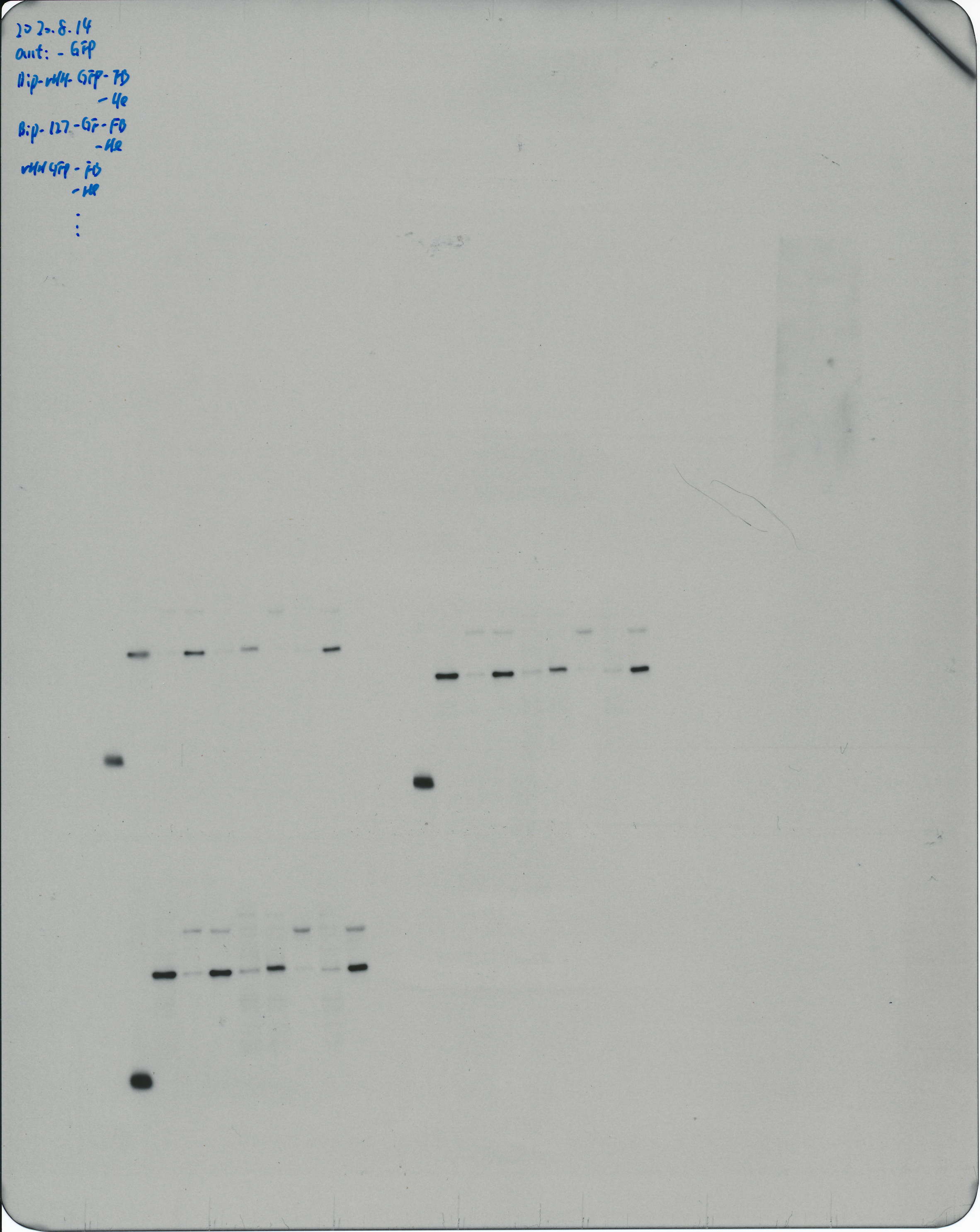

Supplement: Figure 5—source data 1. [file elife-74326-fig5-data1.zip › Figure 5E and 5Fí¬source data/Figure 5E_raw_3.tif]

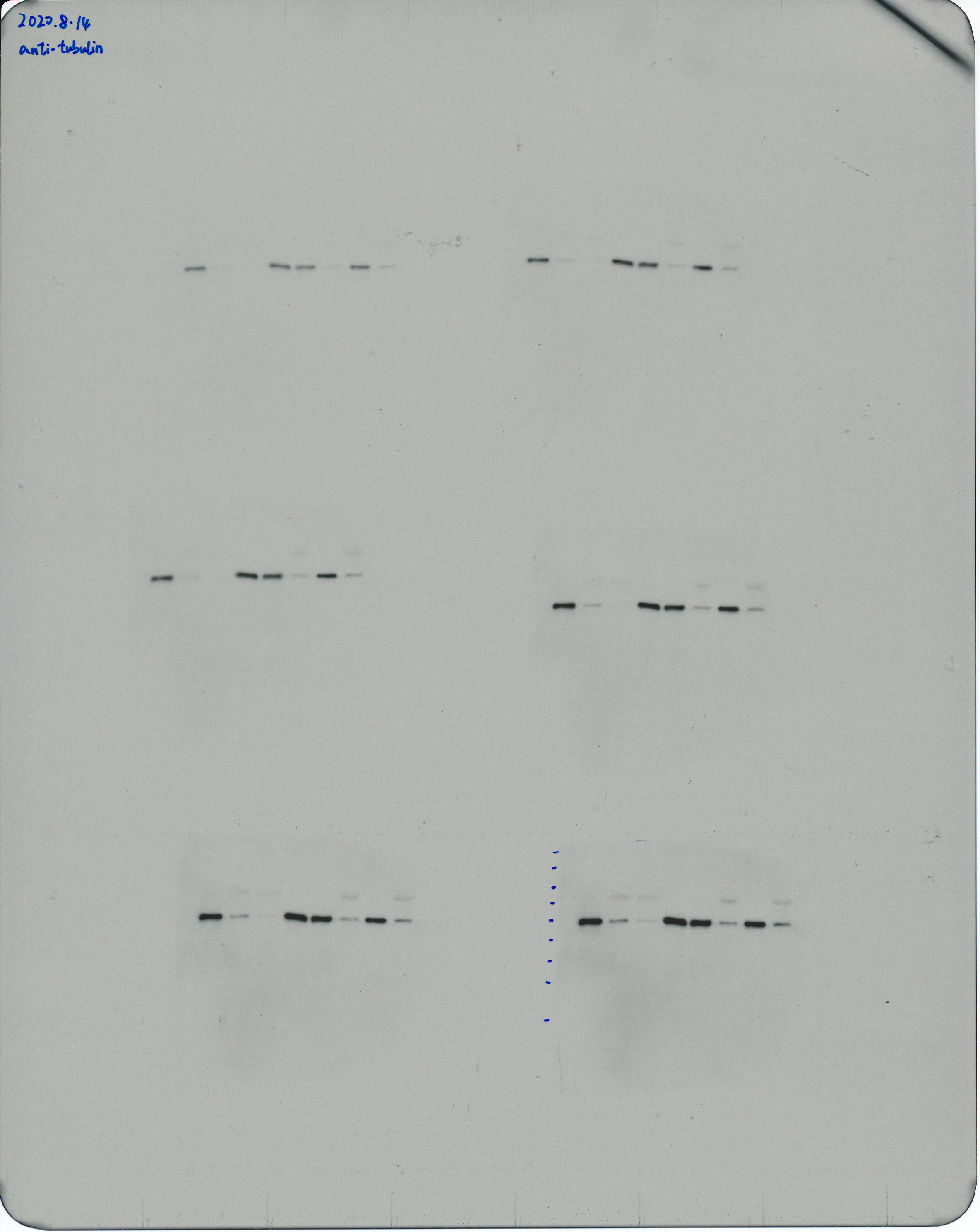

Supplement: Figure 5—source data 1. [file elife-74326-fig5-data1.zip › Figure 5E and 5Fí¬source data/Figure 5E_raw_4.tif]

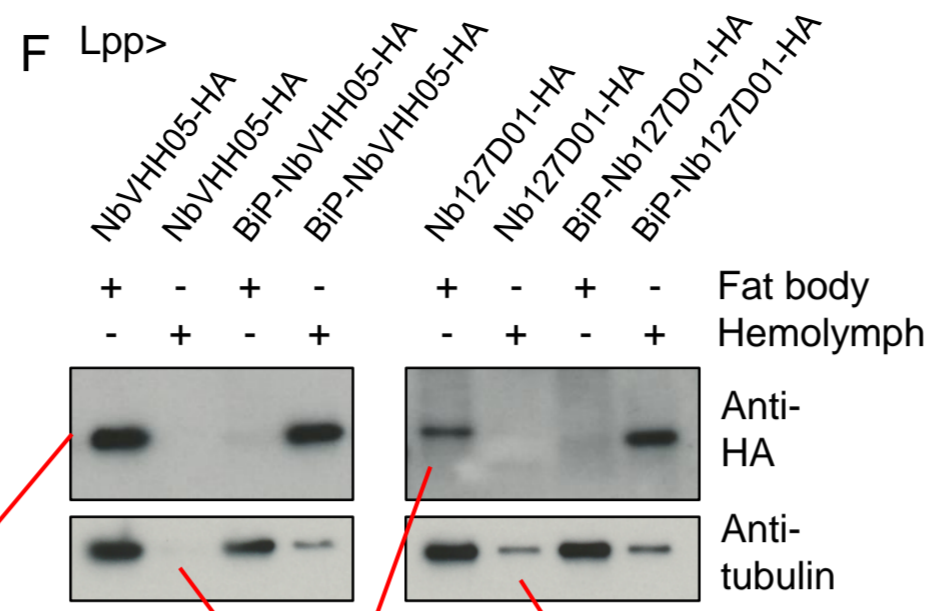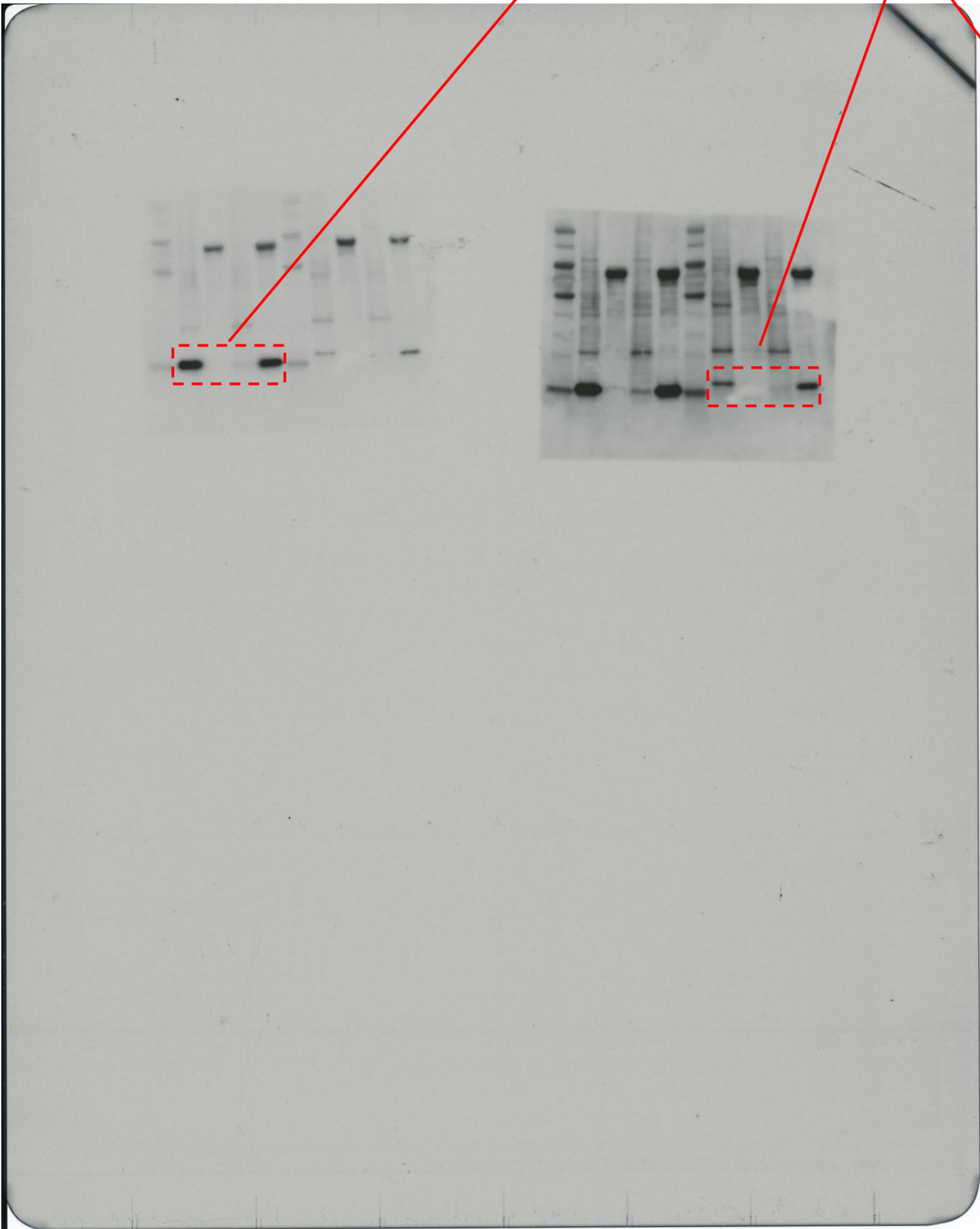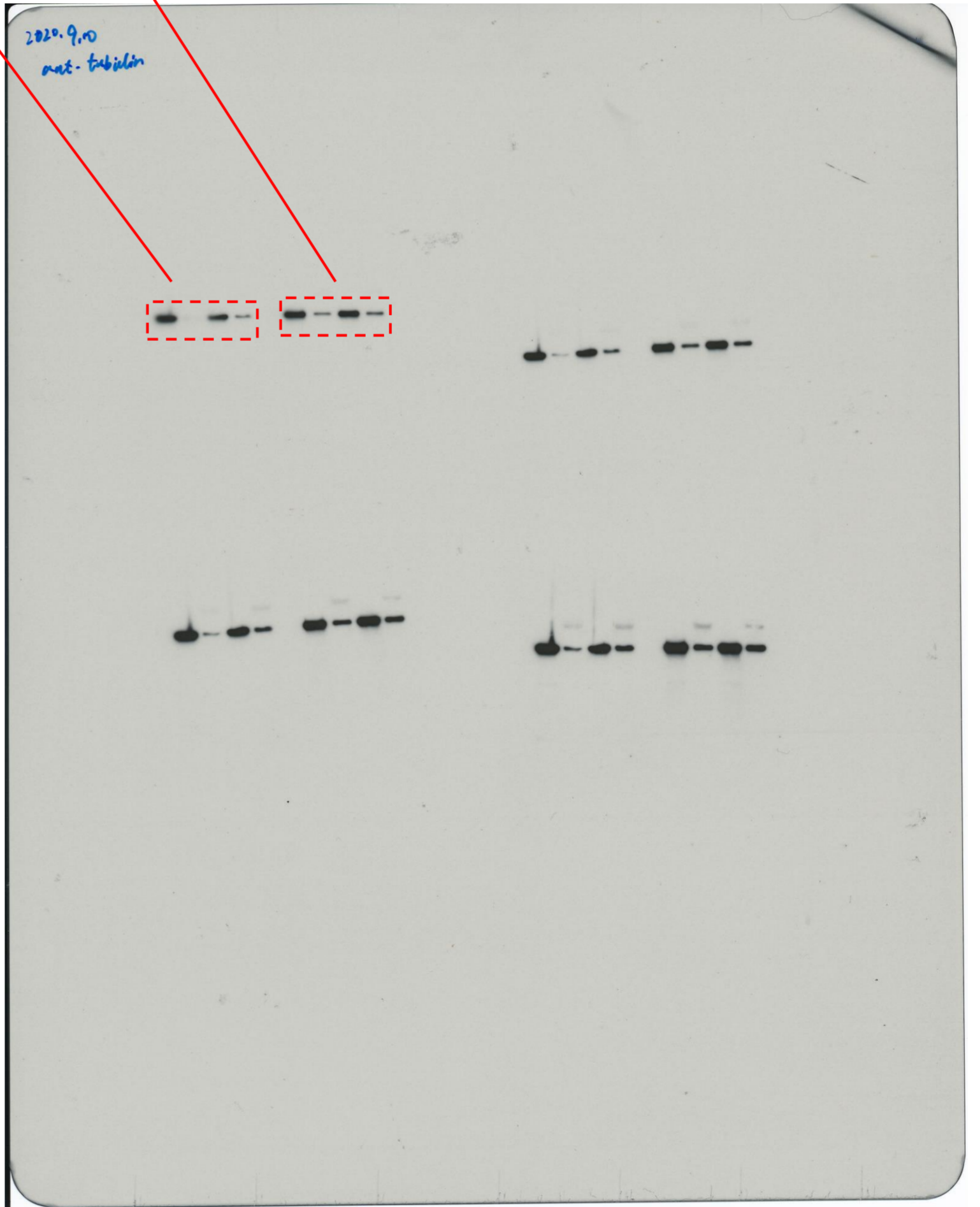

Supplement: Figure 5—source data 1. [file elife-74326-fig5-data1.zip › Figure 5E and 5Fí¬source data/Figure 5F_Crop.pdf]

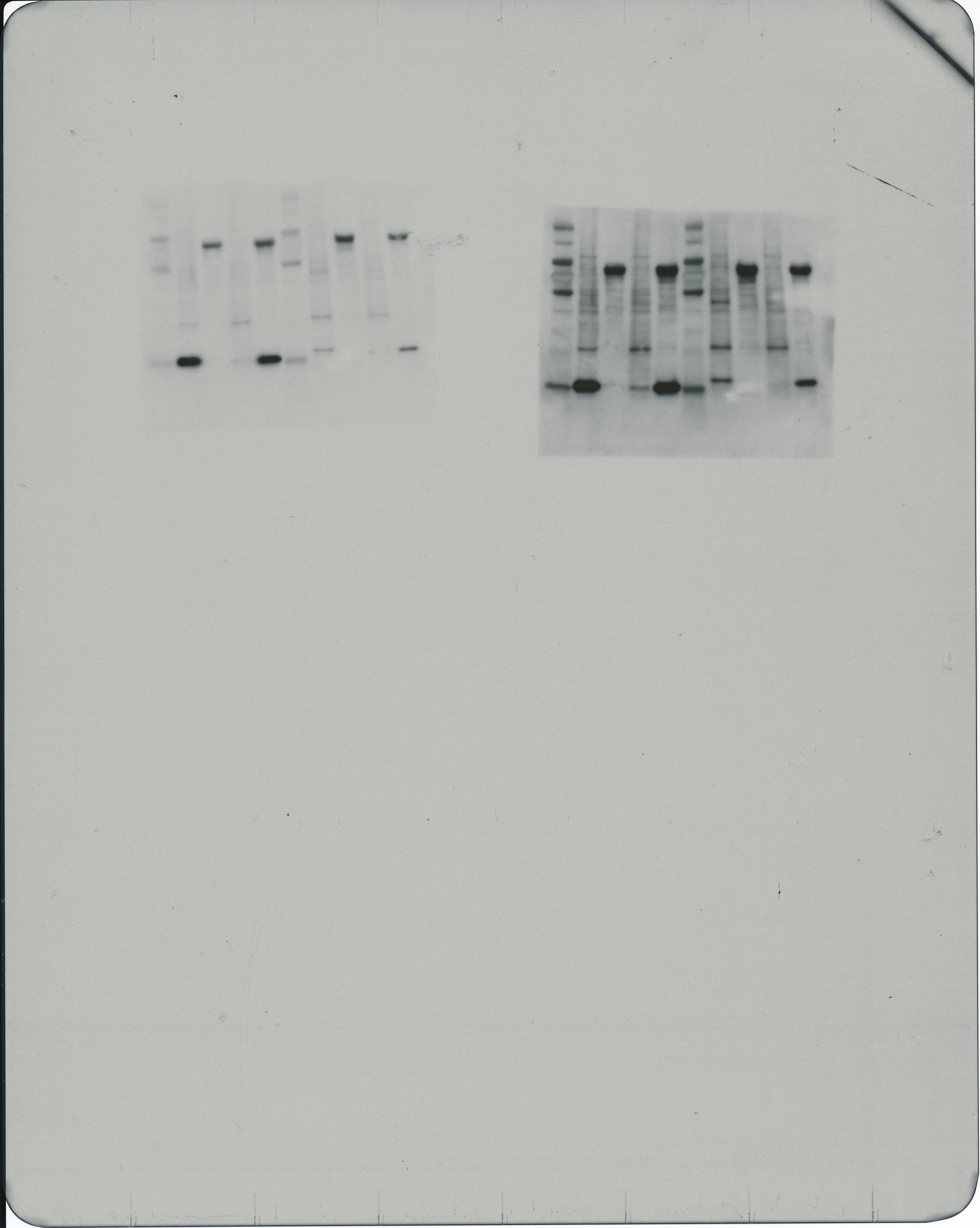

Supplement: Figure 5—source data 1. [file elife-74326-fig5-data1.zip › Figure 5E and 5Fí¬source data/Figure 5F_raw_1.tif]

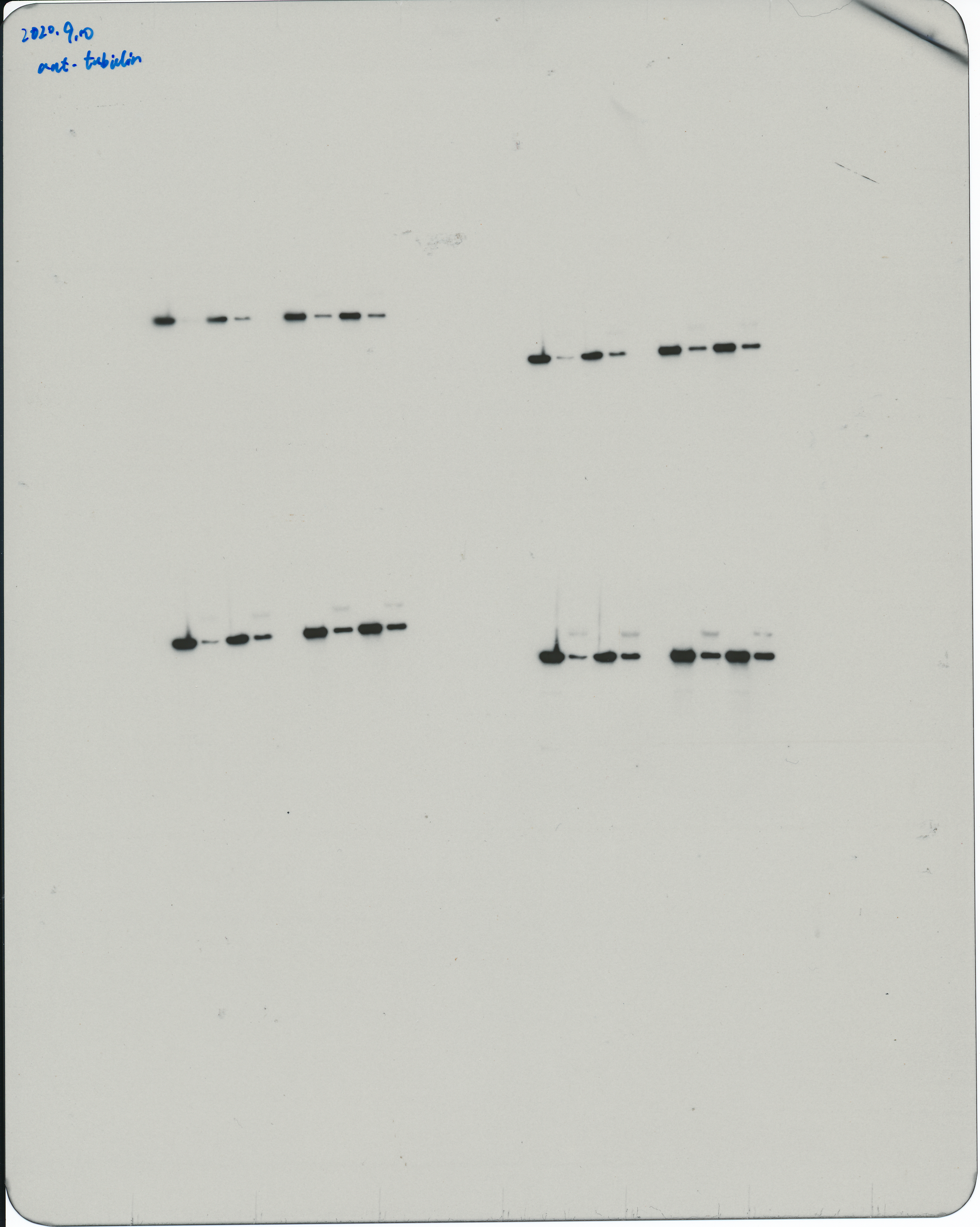

Supplement: Figure 5—source data 1. [file elife-74326-fig5-data1.zip › Figure 5E and 5Fí¬source data/Figure 5F_raw_2.tif]

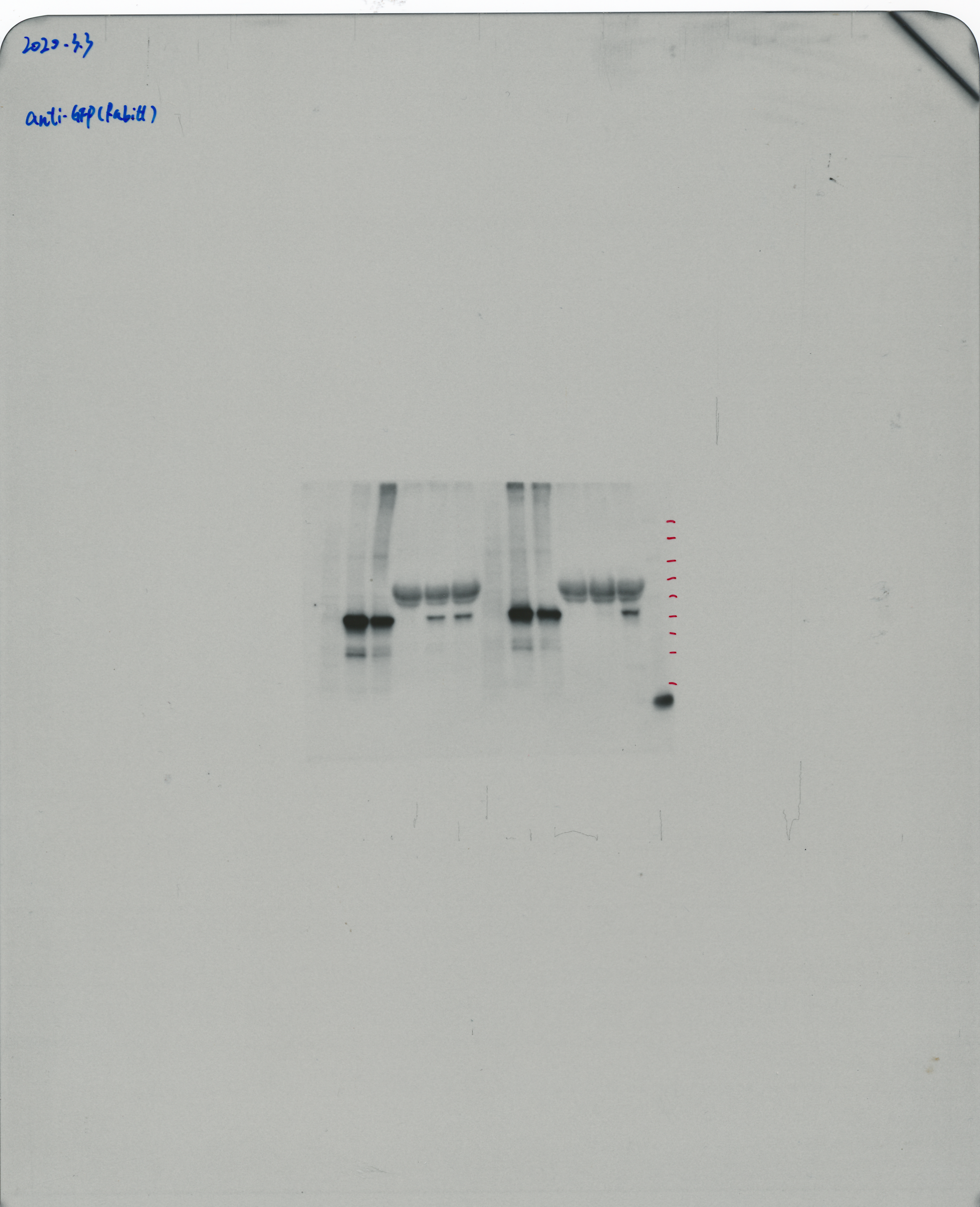

Supplement: Figure 5—figure supplement 1—source data 1. [file elife-74326-fig5-figsupp1-data1.zip › Figure 5í¬figure supplement 1-source data/Figure 5í¬figure supplement 1B_raw1.tif]

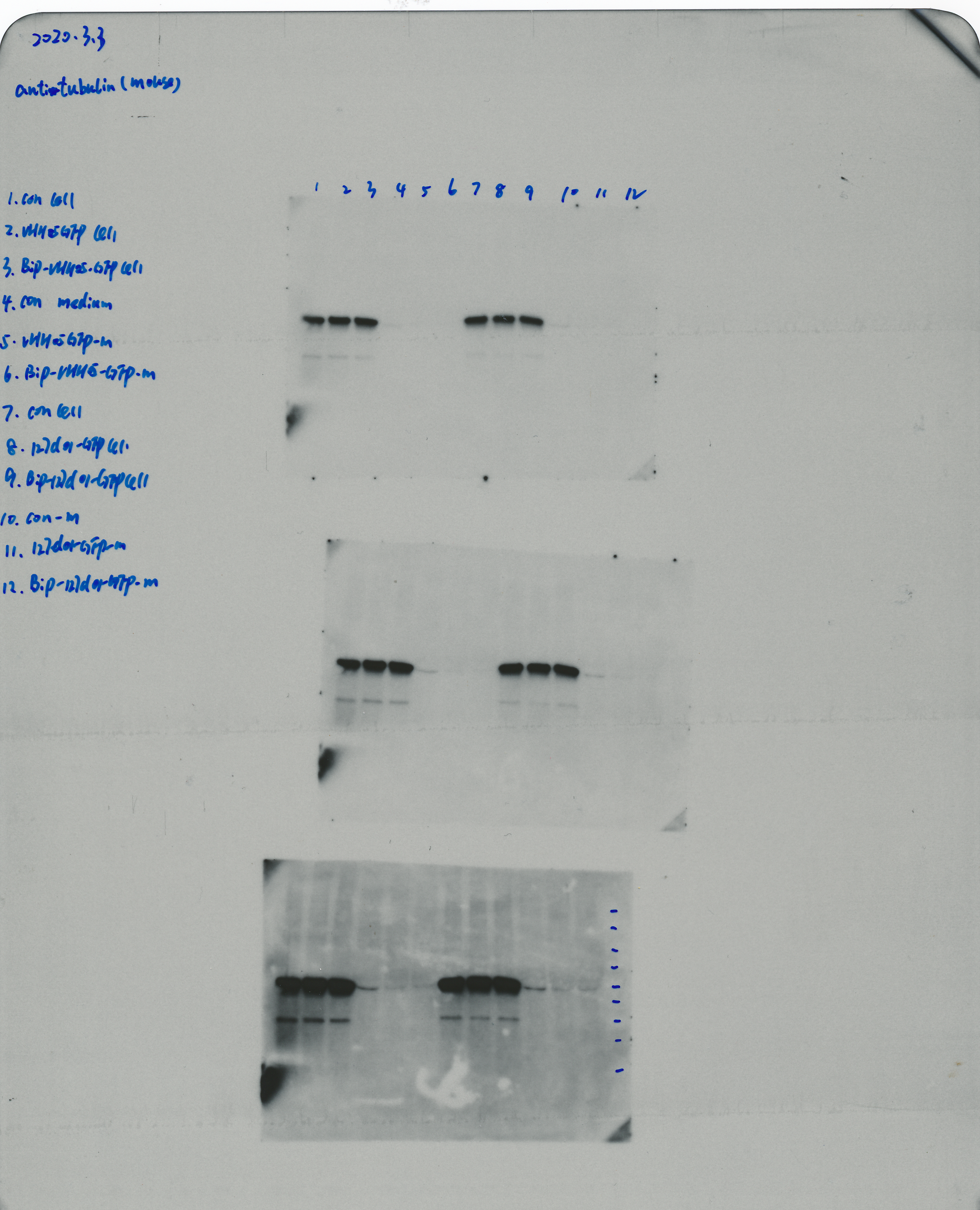

Supplement: Figure 5—figure supplement 1—source data 1. [file elife-74326-fig5-figsupp1-data1.zip › Figure 5í¬figure supplement 1-source data/Figure 5í¬figure supplement 1B_raw2.tif]

2020.3.4

anti-HA (mouse)  
anti-tubulin (mouse)

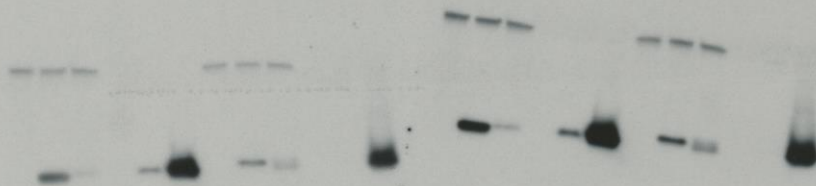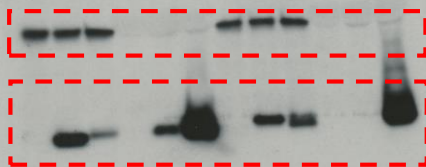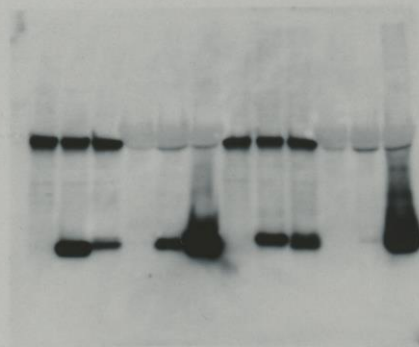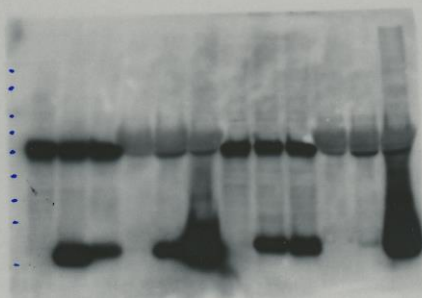

Supplement: Figure 5—figure supplement 1—source data 1. [file elife-74326-fig5-figsupp1-data1.zip › Figure 5í¬figure supplement 1-source data/Figure 5í¬figure supplement 1C_Crop.pdf]

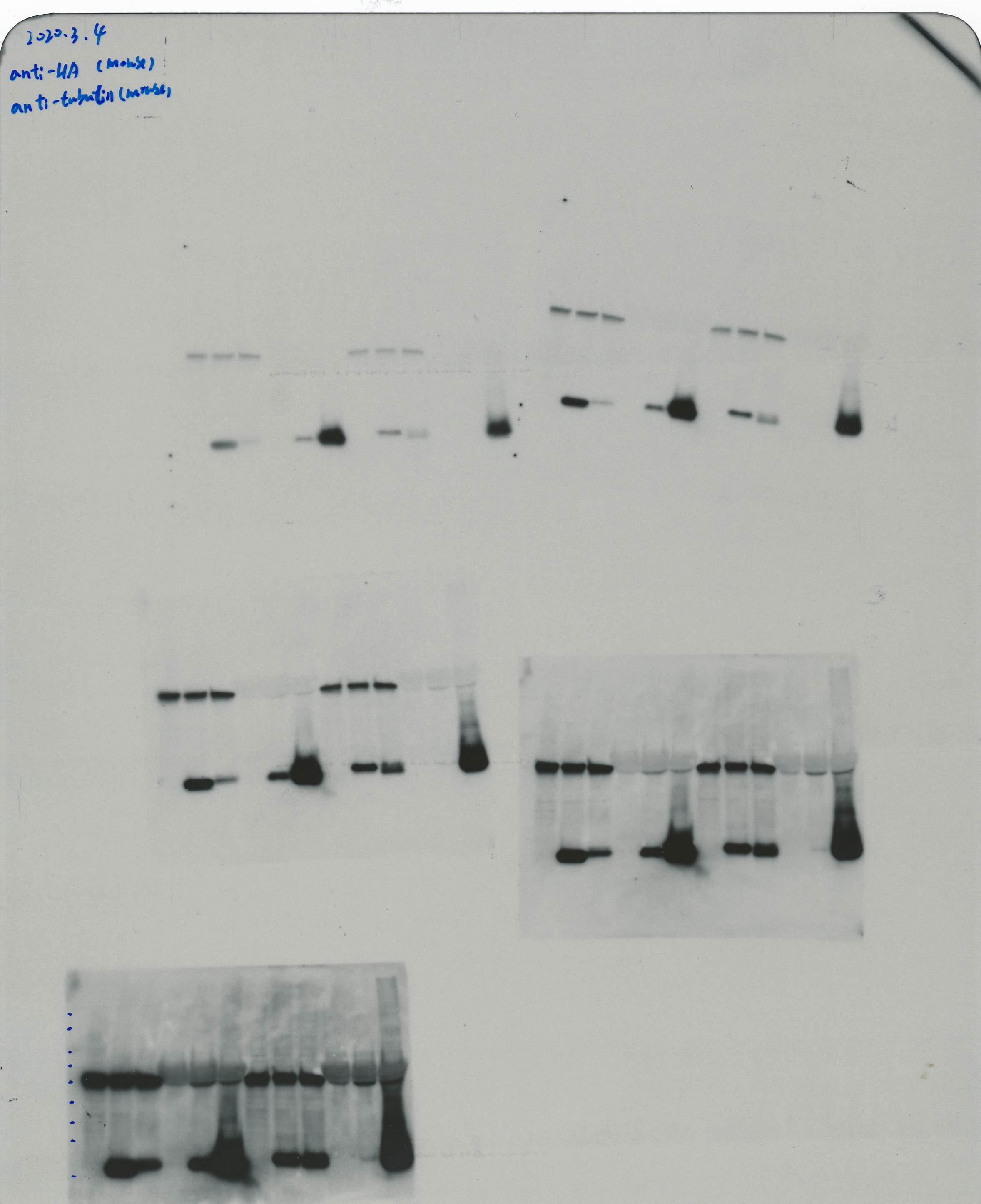

Supplement: Figure 5—figure supplement 1—source data 1. [file elife-74326-fig5-figsupp1-data1.zip › Figure 5í¬figure supplement 1-source data/Figure 5í¬figure supplement 1C_raw3.tif]

2020. 7. 12

1. con - m

2. Aklh

3. D:Gp

4. DLP8

5. pml

6. upd6

7. con - m

8. Rept - m

9. Rept - m ant - m

10. Rept - m

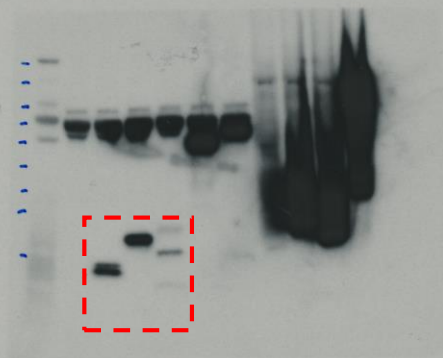

Anti-m

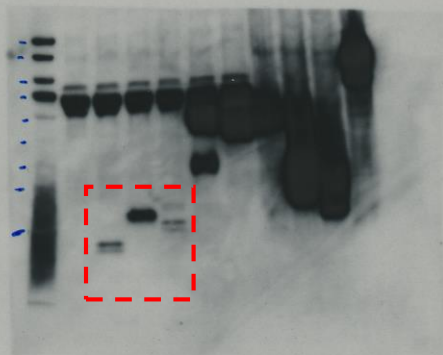

Supplement: Figure 5—figure supplement 1—source data 1. [file elife-74326-fig5-figsupp1-data1.zip › Figure 5í¬figure supplement 1-source data/Figure 5í¬figure supplement 1F_Crop.pdf]

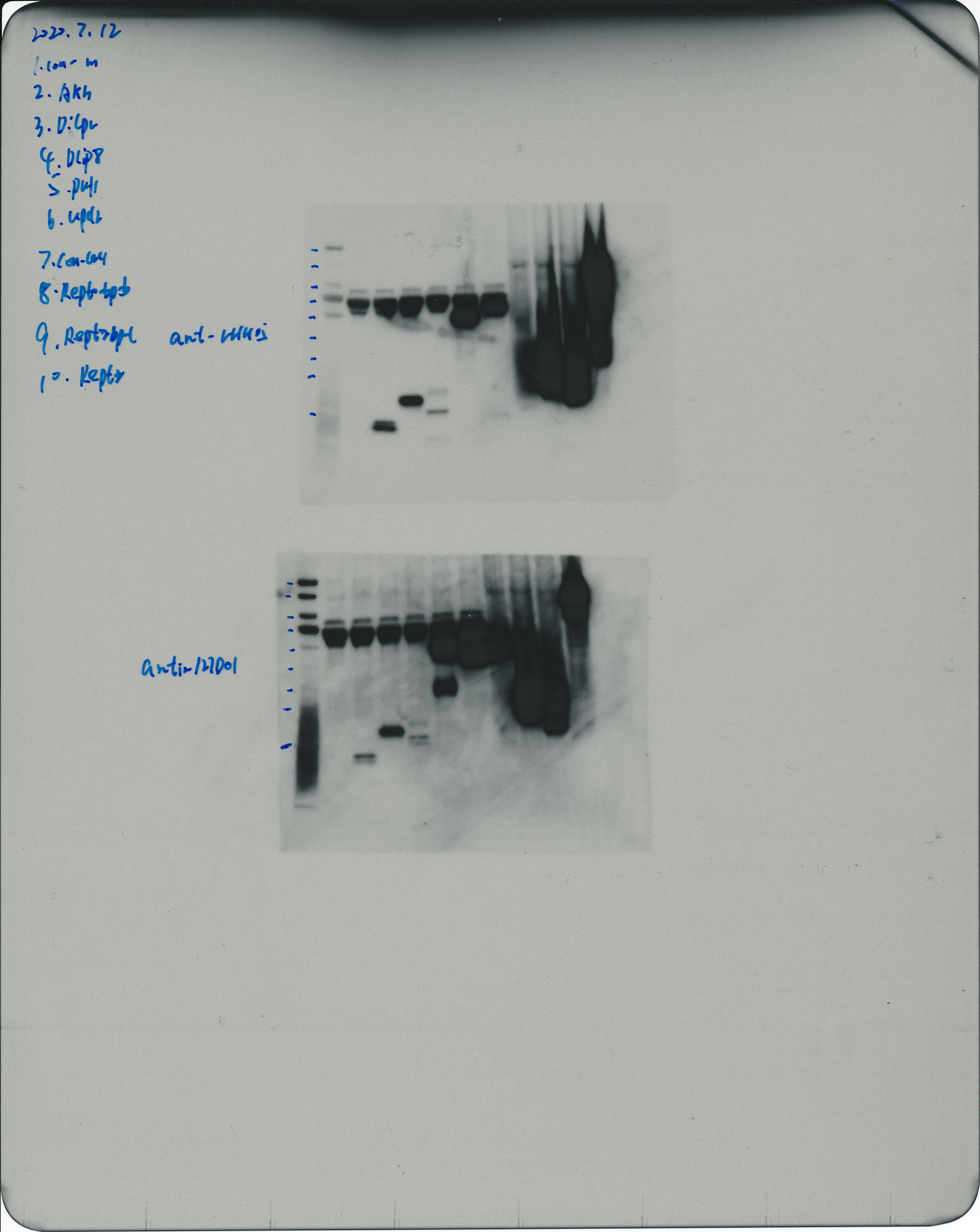

Supplement: Figure 5—figure supplement 1—source data 1. [file elife-74326-fig5-figsupp1-data1.zip › Figure 5í¬figure supplement 1-source data/Figure 5í¬figure supplement 1F_raw.tif]

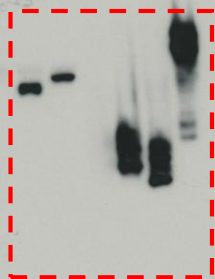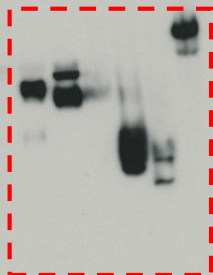

Supplement: Figure 5—figure supplement 1—source data 1. [file elife-74326-fig5-figsupp1-data1.zip › Figure 5í¬figure supplement 1-source data/Figure 5í¬figure supplement 1G_Crop.pdf]

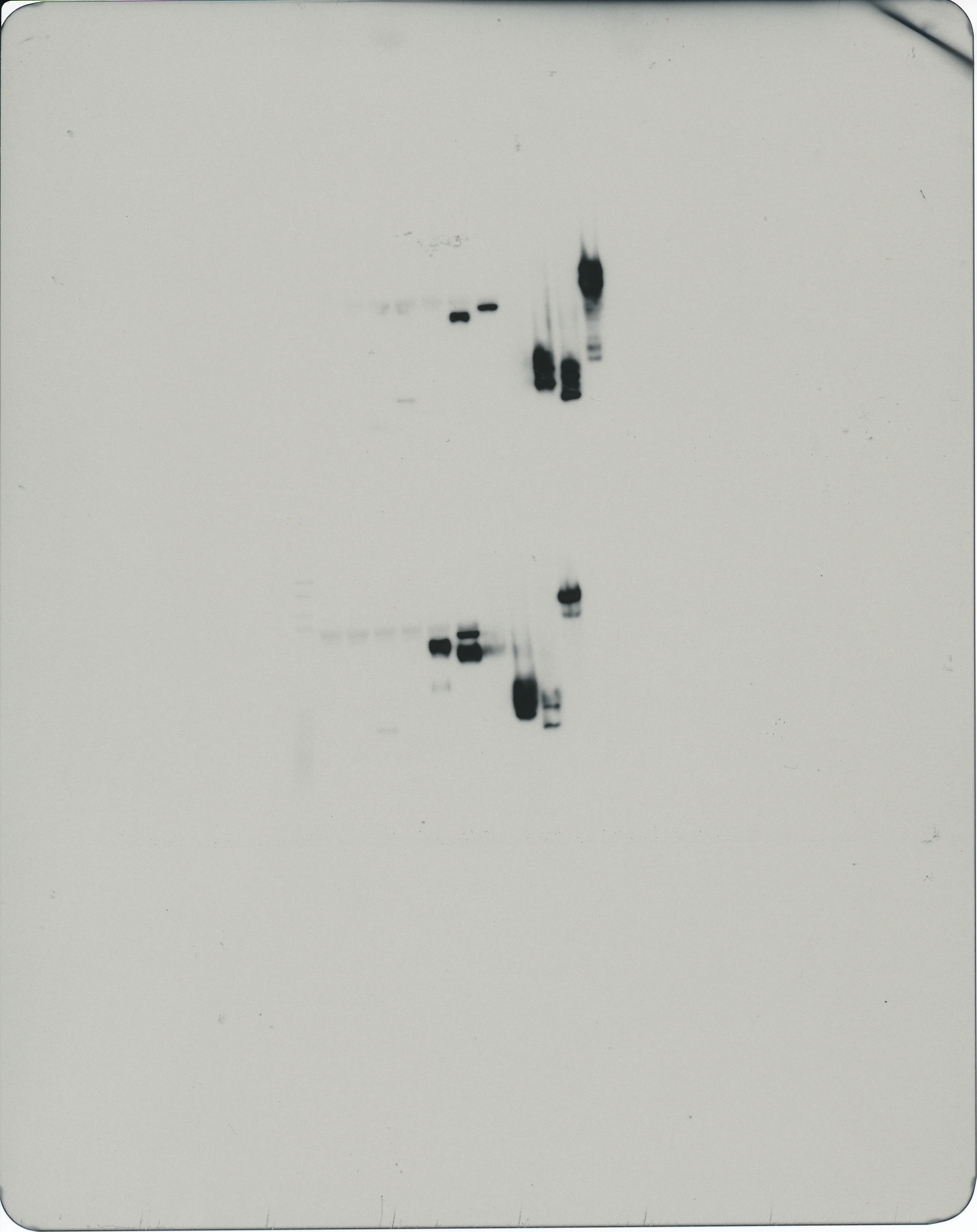

Supplement: Figure 5—figure supplement 1—source data 1. [file elife-74326-fig5-figsupp1-data1.zip › Figure 5í¬figure supplement 1-source data/Figure 5í¬figure supplement 1G_raw.tif]

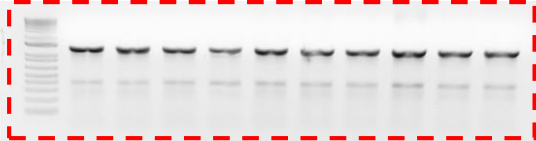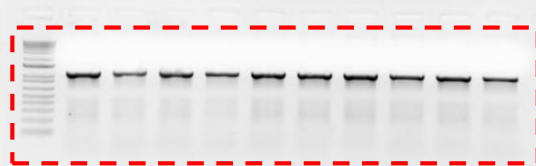

Supplement: Figure 6—source data 1. [file elife-74326-fig6-data1.zip › Figure 6Bí¬source data/Figure 6B_Crop.pdf]

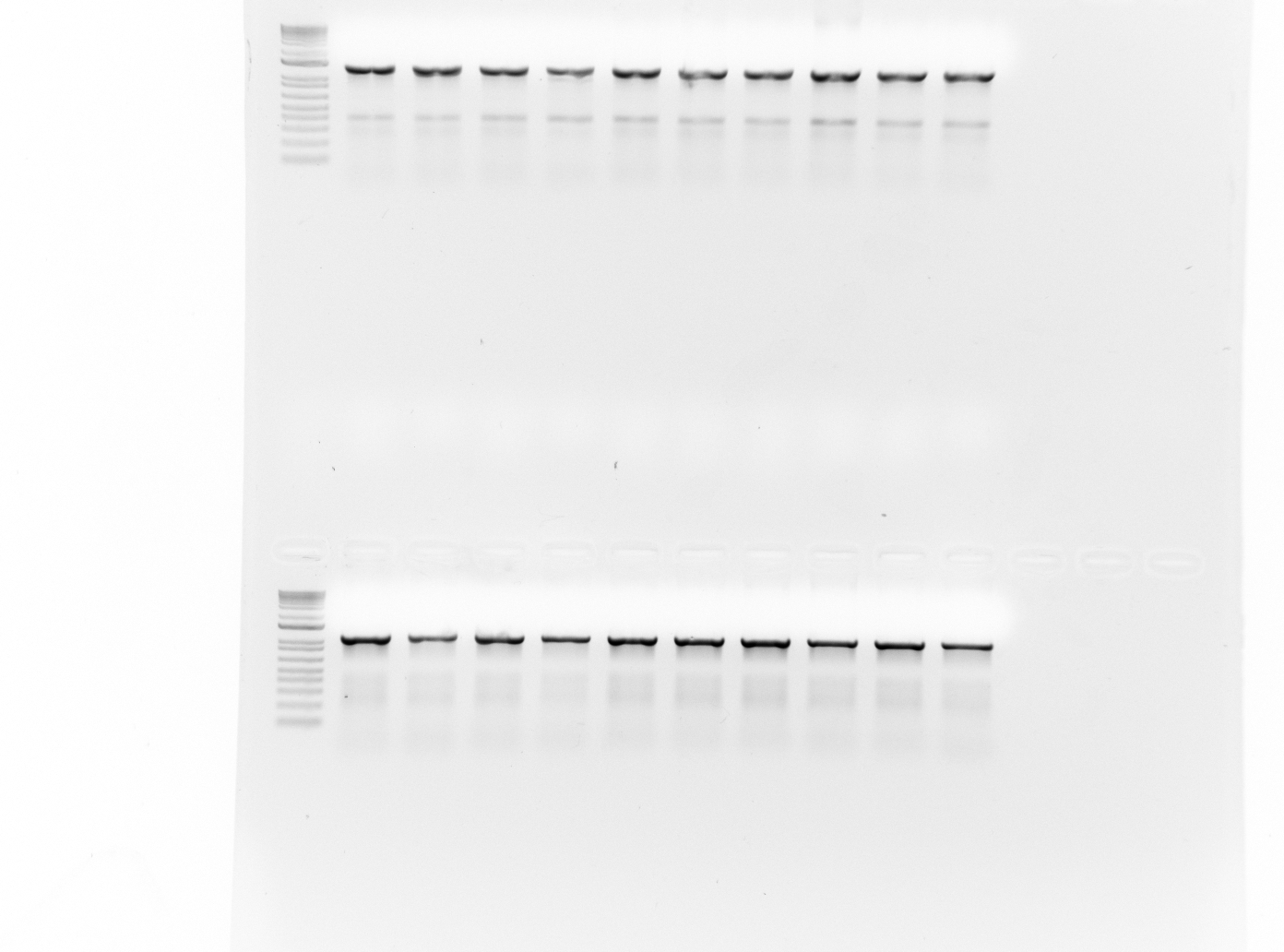

Supplement: Figure 6—source data 1. [file elife-74326-fig6-data1.zip › Figure 6Bí¬source data/Figure 6B_raw.tif]

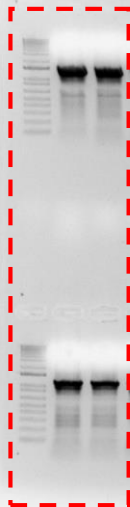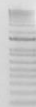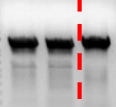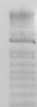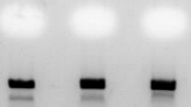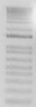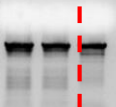

Supplement: Figure 6—figure supplement 1—source data 1. [file elife-74326-fig6-figsupp1-data1.zip › Figure 6í¬figure supplement 1 C'í¬source data/Figure 6í¬figure supplement 1 C'_Crop.pdf]

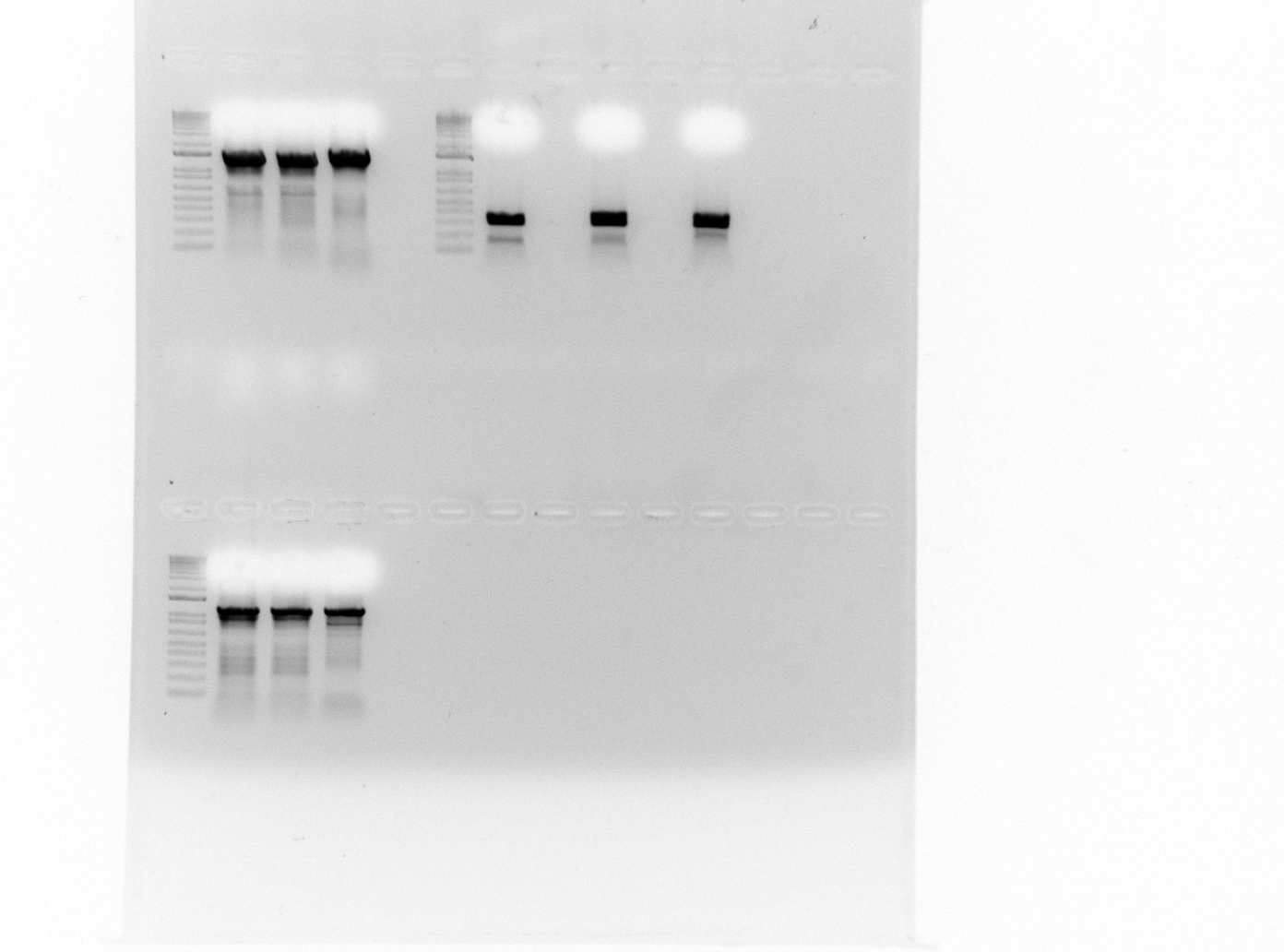

Supplement: Figure 6—figure supplement 1—source data 1. [file elife-74326-fig6-figsupp1-data1.zip › Figure 6í¬figure supplement 1 C'í¬source data/Figure 6í¬figure supplement 1 C'_raw.tif]

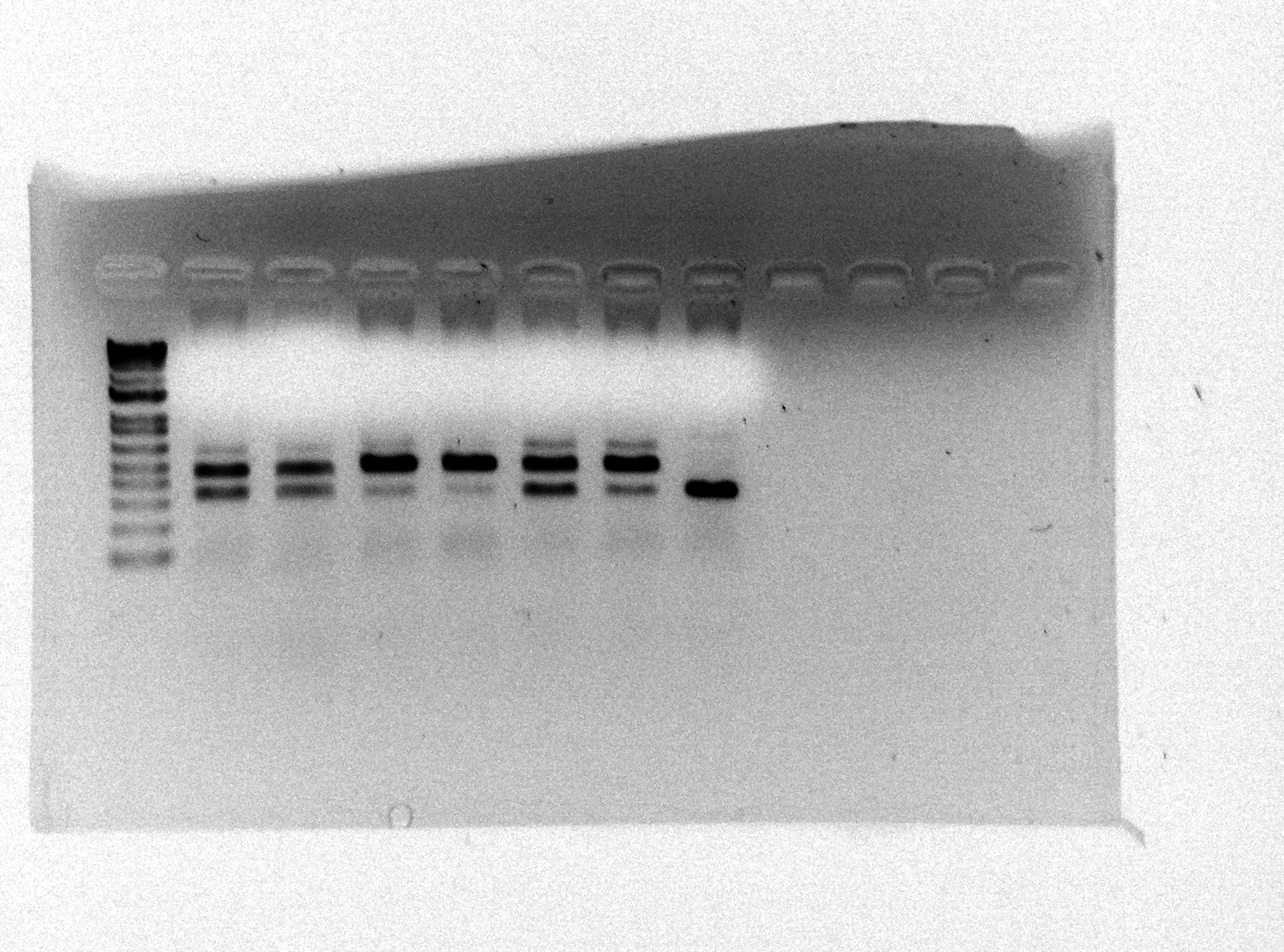

Supplement: Figure 6—figure supplement 2—source data 1. [file elife-74326-fig6-figsupp2-data1.zip › Figure 6í¬figure supplement 2Bí¬source data/Figure 6í¬figure supplement 2B.tif]

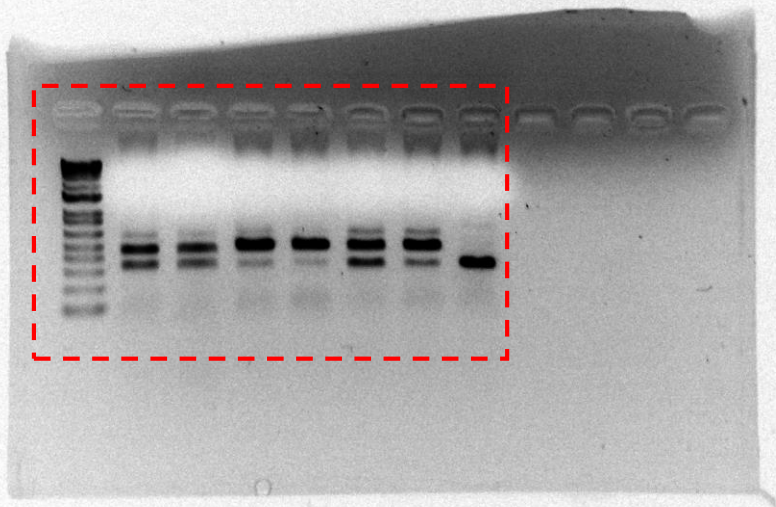

Supplement: Figure 6—figure supplement 2—source data 1. [file elife-74326-fig6-figsupp2-data1.zip › Figure 6í¬figure supplement 2Bí¬source data/Figure 6í¬figure supplement 2B_Crop.pdf]
