# Supplementary material for: Protein visualization and manipulation in Drosophila through the use of epitope tags recognized by nanobodies
Source: eLife. 2022 Jan 25;11:e74326. doi: 10.7554/eLife.74326 (PMC8853664; doi:10.7554/eLife.74326)
Supplement: Figure 3—source data 1. [file elife-74326-fig3-data1.zip › Figure 3í¬source data/Figure 3A_Crop.pdf]

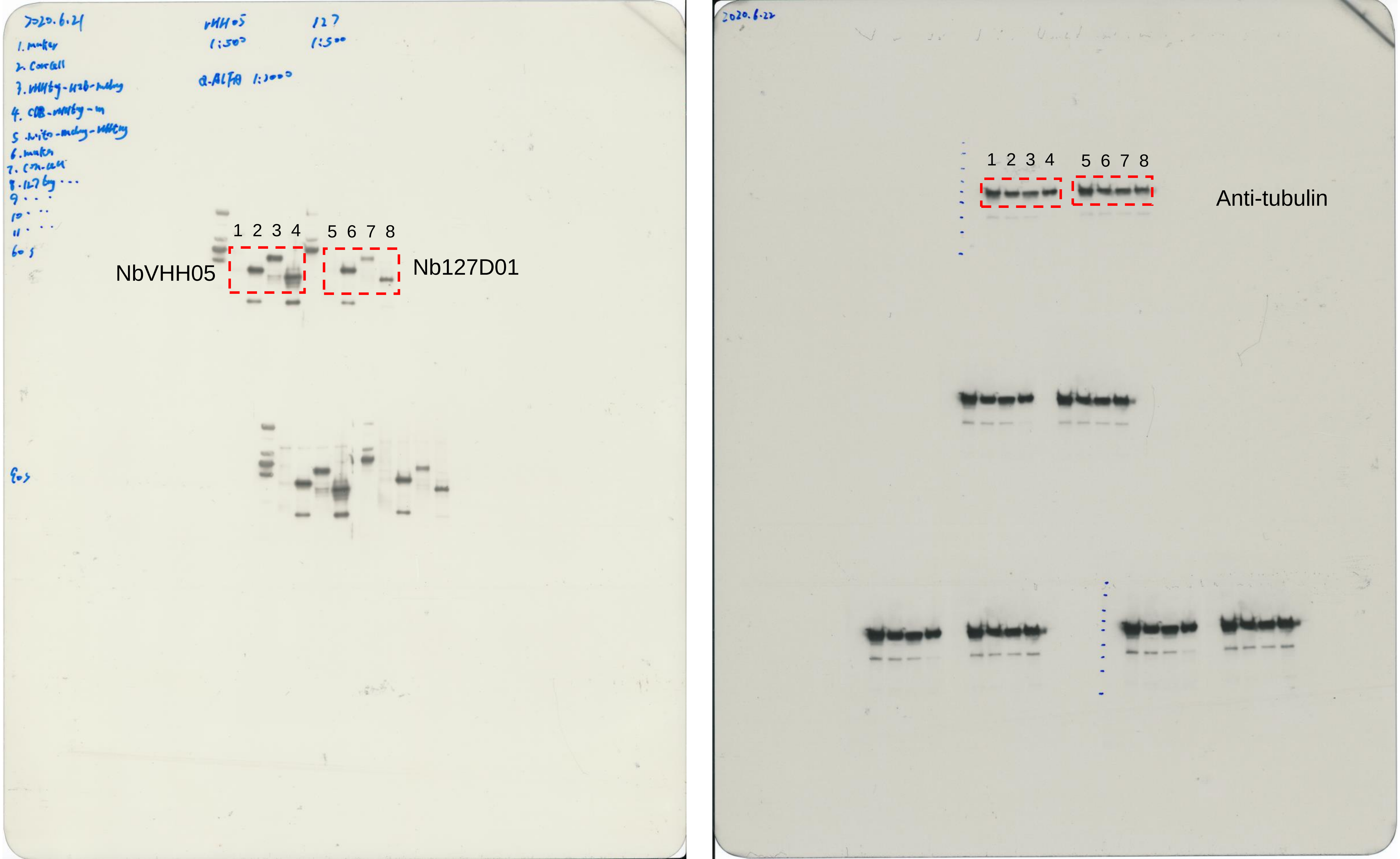
1 2 3 4
5 6 7 8
NbVHH05
Nb127D01
1 2 3 4
5 6 7 8
Anti-tubulin
