# Supplementary material for: Protein visualization and manipulation in Drosophila through the use of epitope tags recognized by nanobodies
Source: eLife. 2022 Jan 25;11:e74326. doi: 10.7554/eLife.74326 (PMC8853664; doi:10.7554/eLife.74326)
Supplement: Figure 5—figure supplement 1—source data 1. [file elife-74326-fig5-figsupp1-data1.zip › Figure 5í¬figure supplement 1-source data/Figure 5í¬figure supplement 1B_Crop.pdf]

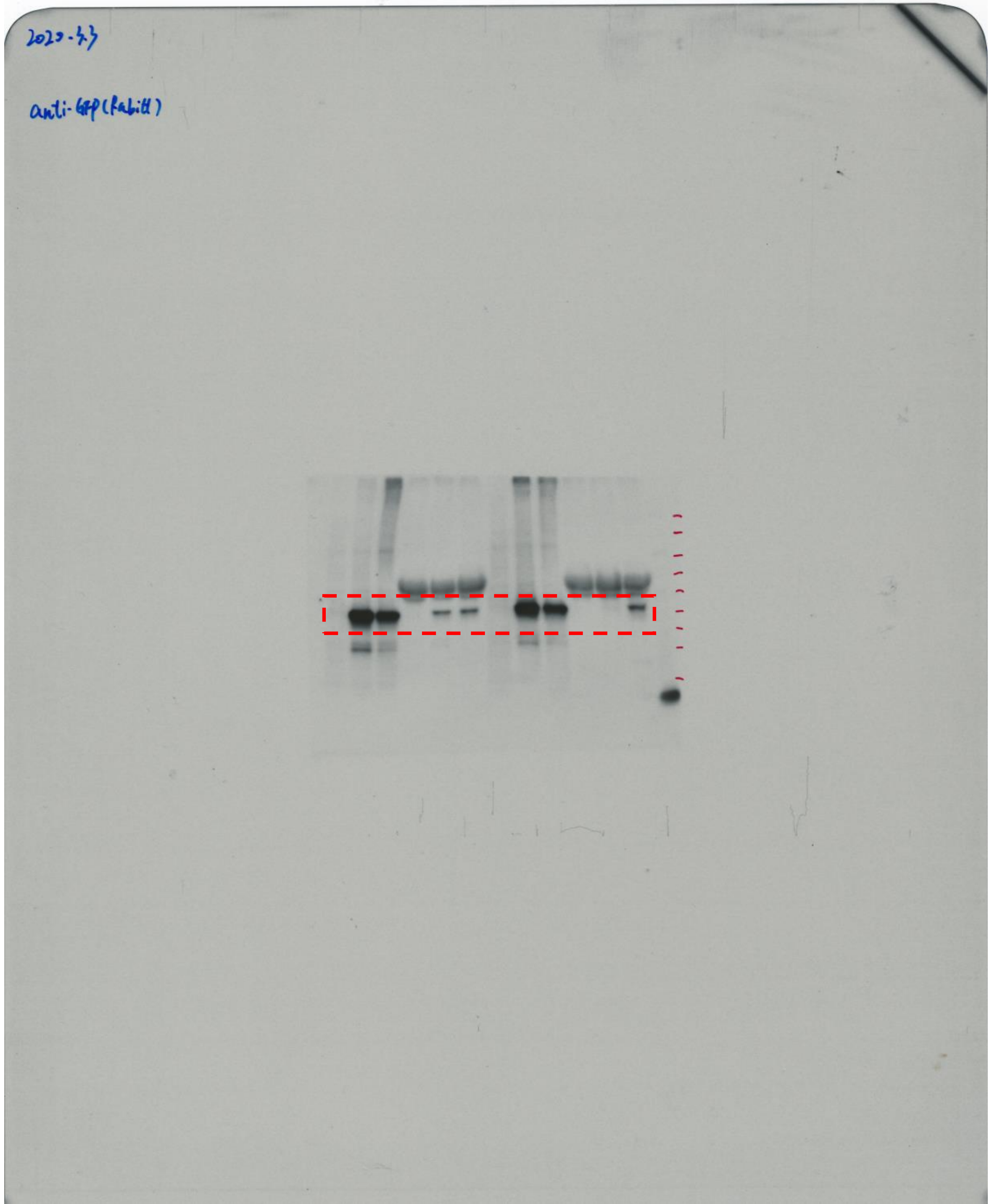

2020.3.3
anti-GFP (Rabbit)

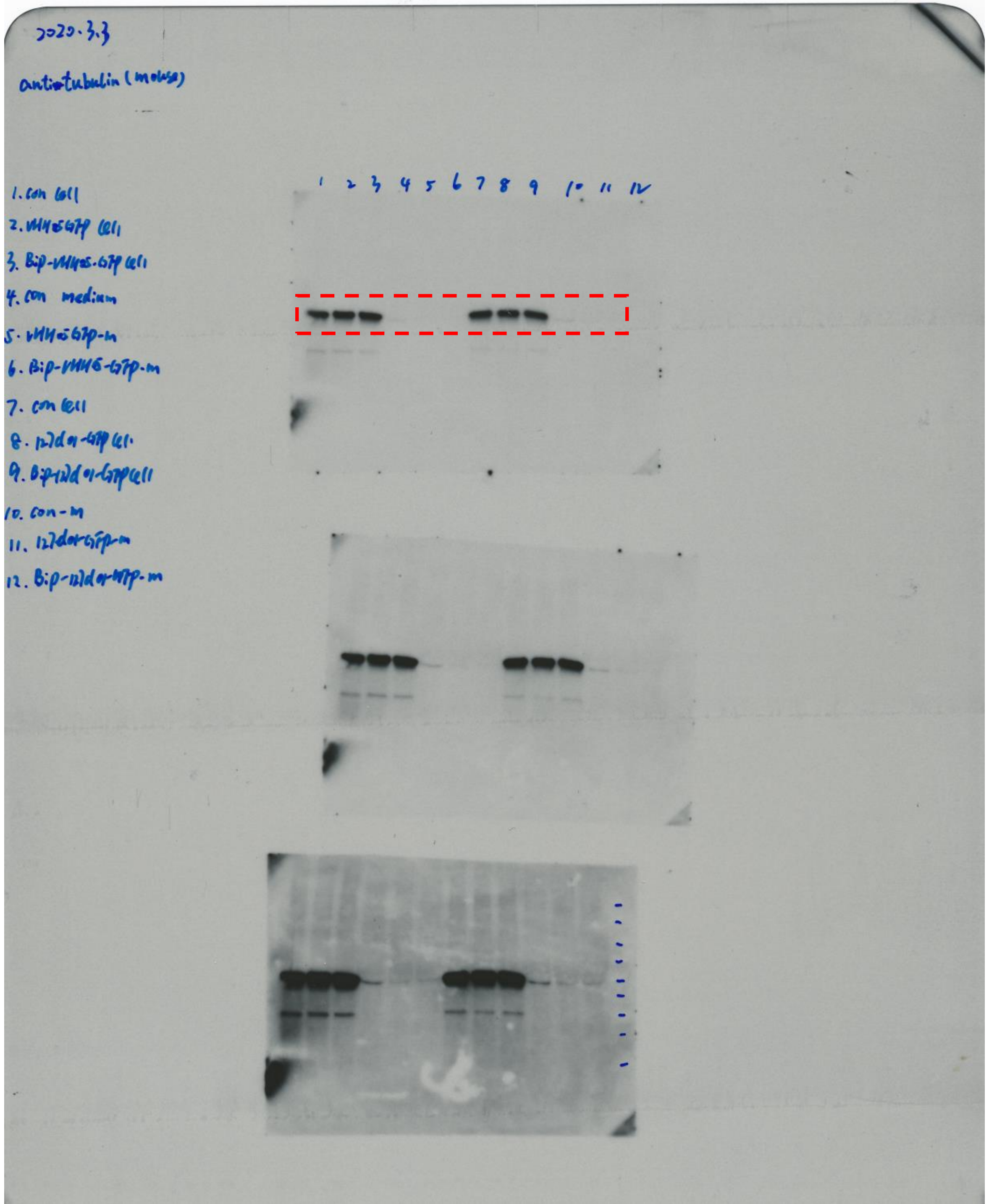

2020.3.3
anti-tubulin (mouse)
1 2 3 4 5 6 7 8 9 10 11 12
1. con cell
4. con medium
7. con cell
10. con-m
