# Supplementary material for: Protein visualization and manipulation in Drosophila through the use of epitope tags recognized by nanobodies
Source: eLife. 2022 Jan 25;11:e74326. doi: 10.7554/eLife.74326 (PMC8853664; doi:10.7554/eLife.74326)
Supplement: Supplementary file 1. [file elife-74326-supp1.docx]

**Table S1. Primers used in this study.**

| Name | Sequence | Purpose |
| --- | --- | --- |
| VHH05-HA-F | TGAATAGGGAATTGGGAATTCCAAAATGCAGGTGCAGCTGCAGGAGT | Cloning VHH05-HA PCR fragment to make pW10-UAS-NbVHH05-HA. |
| VHH05-HA-R | TCAGAACTAGTTTGCTCTAGATTAAGCGTAGTCTGGGACGTCGTATGGGTATGATCCCCCACCGCTGCCACCTCCTGAGGAGACGGTGACCTGG |  |
| 127D01-HA-F | GAATAGGGAATTGGGAATTCCAAAATGGAGGTTCAATTAGTGGAAT | Cloning 127D01-HA PCR fragment to make pW10-UAS-Nb127D01-HA. |
| 127D01-HA-R | TCAGAACTAGTTTGCTCTAGATTAAGCGTAGTCTGGGACGTCGTATGGGTATGATCCCCCACCGCTGCCACCTCCAGAAGAGACTGTGACCTGC |  |
| VHH05-HA-F | TGAATAGGGAATTGGGAATTCCAAAATGCAGGTGCAGCTGCAGGAGT | Cloning VHH05 PCR fragment to make pW10-UAS-NbVHH05-GFP. |
| Linker-R: | TGATCCCCCACCGCTGCCAC |  |
| GFP-Nb-F | GTGGCAGCGGTGGGGGATCAGTGAGCAAGGGCGAGGAGCT | Cloning GFP PCR fragment to make pW10-UAS-NbVHH05-GFP or pW10-UAS-Nb127D01-GFP. |
| GFP-Nb-R | AGATCAGAACTAGTTTGCTCTAGATTACTTGTACAGCTCGTCCA |  |
| 127d01-HA-F | GAATAGGGAATTGGGAATTCCAAAATGGAGGTTCAATTAGTGGAAT | Cloning 127D01 PCR fragment to make pW10-UAS-Nb127D01-GFP. |
| Linker-R | TGATCCCCCACCGCTGCCAC |  |
| BiP-VHH05-HA-F | TGAATAGGGAATTGGGAATTCCAAAATGAAGTTATGCATATTACTGGCCGTCGTGGCCTTTGTTGGCCTCTCGCTCGGGCAGGTGCAGCTGCAGGAGT | Cloning BiP-VHH05-HA PCR fragment to make pW10-BiP-UAS-BiP-NbVHH05-HA. |
| VHH05-HA-R | TCAGAACTAGTTTGCTCTAGATTAAGCGTAGTCTGGGACGTCGTATGGGTATGATCCCCCACCGCTGCCACCTCCTGAGGAGACGGTGACCTGG |  |
| BiP-127D01-HA-F | TGAATAGGGAATTGGGAATTCCAAAATGAAGTTATGCATATTACTGGCCGTCGTGGCCTTTGTTGGCCTCTCGCTCGGGGAGGTTCAATTAGTGGAAT | Cloning BiP-127D01-HA PCR fragment to make pW10-UAS-BiP-Nb127D01-HA. |
| 127D01-HA-R | TCAGAACTAGTTTGCTCTAGATTAAGCGTAGTCTGGGACGTCGTATGGGTATGATCCCCCACCGCTGCCACCTCCAGAAGAGACTGTGACCTGC |  |
| BiP-VHH05-HA-F | TGAATAGGGAATTGGGAATTCCAAAATGAAGTTATGCATATTACTGGCCGTCGTGGCCTTTGTTGGCCTCTCGCTCGGGCAGGTGCAGCTGCAGGAGT | Cloning BiP-VHH05-GFP PCR fragment to make pW10-BiP-UAS-BiP-NbVHH05-GFP. |
| GFP-Nb-R | AGATCAGAACTAGTTTGCTCTAGATTACTTGTACAGCTCGTCCA |  |
| BiP-127D01-HA-F | TGAATAGGGAATTGGGAATTCCAAAATGAAGTTATGCATATTACTGGCCGTCGTGGCCTTTGTTGGCCTCTCGCTCGGGGAGGTTCAATTAGTGGAAT | Cloning BiP-127D01-GFP PCR fragment to make pW10-UAS-BiP-Nb127D01-GFP. |
| GFP-Nb-R | AGATCAGAACTAGTTTGCTCTAGATTACTTGTACAGCTCGTCCA |  |
| pWalium-Colony-F | AGCGCAGCTGAACAAGCTA | Colony PCR for all pWalium 10 insertion. |
| pWalium-Colony-R | TTTGCAATCTGTAAGCATAAGCA |  |
| pAW-nb-F | TACCATCCAGCCTCCGGACTCTAGAGAATTGGGAATTCCAAAATG | Cloning VHH05-GFP or 127D01-GFP PCR fragments to make pAW-NbVHH05-GFP or pAW-Nb127D01-GFP. |
| pAW-nb-R | TCCTTCACAAAGATCCTGCTAGCTTACTTGTACAGCTCGTCCA |  |
| pAW-nb-F | TACCATCCAGCCTCCGGACTCTAGA GAATTGGGAATTCCAAAATG | Cloning VHH05 or 127D01 PCR fragments to make pAW-NbVHH05-mCherry or pAW-Nb127D01-mCherry. |
| linker-R | TGATCCCCCACCGCTGCCAC |  |
| mCherry-F | GTGGCAGCGGTGGGGGATCAatggtgagcaagggcgagga | Cloning mCherry fragment to make pAW-NbVHH05-mCherry or pAW-Nb127D01-mCherry. |
| mCherry-R | GTTCCTTCACAAAGATCCTGCTAGCTCAcagctcgtccttctcgctacccttgtacagctcgtccatgc |  |
| H2B-mCherry-F | TACCATCCAGCCTCCGGACTCTAGAatggctccgaaaactagtgg | Cloning H2B-mCherry-VHH05 fragment to make pAW-H2B-mCherry-VHH05. |
| VHH05-H2B-R | TCCTTCACAAAGATCCTGCTAGCTCAGGAAATCTGCCGTGCCAATTCTTTAGCCTCTTGATCTGCCTGtccggatcccttgtacagctcgtccatgc |  |
| H2B-mCherry-F | TACCATCCAGCCTCCGGACTCTAGAatggctccgaaaactagtgg | Cloning H2B-mCherry-127D01 fragment to make pAW-H2B-mCherry-127D01. |
| 127D01-H2B-R | TCCTTCACAAAGATCCTGCTAGCTCAATCCTCGCCTTTCCAGAAATCTTCAAAACTtccggatcccttgtacagctcgtccatgc |  |
| CD8-mCherry-F | TACCATCCAGCCTCCGGACTCTAGAatggcctcaccgttgacccg | Cloning CD8-mCherry-VHH05 fragment to make pAW-CD8-mCherry-VHH05. |
| VHH05-H2B-R | TCCTTCACAAAGATCCTGCTAGCTCAGGAAATCTGCCGTGCCAATTCTTTAGCCTCTTGATCTGCCTGtccggatcccttgtacagctcgtccatgc |  |
| CD8-mCherry-F | TACCATCCAGCCTCCGGACTCTAGAatggcctcaccgttgacccg | Cloning CD8-mCherry-127D01 fragment to make pAW-CD8-mCherry-127D01. |
| 127D01-H2B-R | TCCTTCACAAAGATCCTGCTAGCTCAATCCTCGCCTTTCCAGAAATCTTCAAAACTtccggatcccttgtacagctcgtccatgc |  |
| mito-mCherry-F | TACCATCCAGCCTCCGGACTCTAGAatgagtctgacttccagttc | Cloning mito-mCherry-VHH05 fragment to make pAW-mito-mCherry-VHH05. |
| VHH05-H2B-R | TCCTTCACAAAGATCCTGCTAGCTCAGGAAATCTGCCGTGCCAATTCTTTAGCCTCTTGATCTGCCTGtccggatcccttgtacagctcgtccatgc |  |
| mito-mCherry-F | TACCATCCAGCCTCCGGACTCTAGAatgagtctgacttccagttc | Cloning mito-mCherry-127D01 fragment to make pAW-mito-mCherry-127D01. |
| 127D01-H2B-R | TCCTTCACAAAGATCCTGCTAGCTCAATCCTCGCCTTTCCAGAAATCTTCAAAACTtccggatcccttgtacagctcgtccatgc |  |
| mCherry-VHH05-F | ctctcatctgctaccacagcggatccggaCAGGCAGATCAAGAGGCTAAAGAATTGGCACGGCAGATTTCCggatccggaatggtgagcaagggcgagga | Cloning VHH05-mCherry fragment to make pAW-CD8-VHH05-mCherry. |
| mCherry-VHH05-R | GTTCCTTCACAAAGATCCTGCTAGCTCActtgtacagctcgtccatgc |  |
| mCherry-127D01-F | ctctcatctgctaccacagcggatccggaAGTTTTGAAGATTTCTGGAAAGGCGAGGATggatccggaatggtgagcaagggcgagga | Cloning 127D01-mCherry fragment to make pAW-CD8-127D01-mCherry. |
| mCherry-VHH05-R | GTTCCTTCACAAAGATCCTGCTAGCTCActtgtacagctcgtccatgc |  |
| AR205-GFP-127D01 1X | AACGGCATCAAGGTGAACTTCAAGATCCGCCACAACATCGAGGACGGCAGCGTGCAGCTCGCCGACCACTACCAGCAGAACACCCCCATCGGCGACGGCCCCGTGCTGCTGCCCGACAACCACTACCTGAGCACCCAGTCCGCCCTGAGCAAAGACCCCAACGAGAAGCGCGATCACATGGTCCTGCTGGAGTTCGTGACCGCCGCCGGGATCACTCTCGGCATGGACGAGCTGTACAAGGGCTCCGGCAGTTTTGAGGACTTTTGGAAAGGCGAGGATTAGGGTTCCGGTGGATCCGGA | Cloning synthesized GFP fragment with 1x127D01 tag to make pAW-HGP-BiP-GFP-1x127D01. |
| AR206-GFP-127D01 2X | AACGGCATCAAGGTGAACTTCAAGATCCGCCACAACATCGAGGACGGCAGCGTGCAGCTCGCCGACCACTACCAGCAGAACACCCCCATCGGCGACGGCCCCGTGCTGCTGCCCGACAACCACTACCTGAGCACCCAGTCCGCCCTGAGCAAAGACCCCAACGAGAAGCGCGATCACATGGTCCTGCTGGAGTTCGTGACCGCCGCCGGGATCACTCTCGGCATGGACGAGCTGTACAAGGGCTCCGGCAGTTTTGAGGACTTTTGGAAAGGCGAGGATGGTAGCGGAAGTTTCGAAGACTTCTGGAAGGGAGAGGATTAGGGTTCCGGTGGATCCGGA | Cloning synthesized GFP fragment with 2x127D01 tag to make pAW-HGP-BiP-GFP-2x127D01. |
| AR207-GFP-127D01 3X | AACGGCATCAAGGTGAACTTCAAGATCCGCCACAACATCGAGGACGGCAGCGTGCAGCTCGCCGACCACTACCAGCAGAACACCCCCATCGGCGACGGCCCCGTGCTGCTGCCCGACAACCACTACCTGAGCACCCAGTCCGCCCTGAGCAAAGACCCCAACGAGAAGCGCGATCACATGGTCCTGCTGGAGTTCGTGACCGCCGCCGGGATCACTCTCGGCATGGACGAGCTGTACAAGGGCTCCGGCAGTTTTGAGGACTTTTGGAAAGGCGAGGATGGTAGCGGAAGTTTCGAAGACTTCTGGAAGGGAGAGGATGGCTCCGGAAGCTTTGAAGACTTTTGGAAAGGAGAAGACTAGGGTTCCGGTGGATCCGGA | Cloning synthesized GFP fragment with 3x127D01 tag to make pAW-HGP-BiP-GFP-3x127D01. |
| AR208-GFP-VHH05 1X | AACGGCATCAAGGTGAACTTCAAGATCCGCCACAACATCGAGGACGGCAGCGTGCAGCTCGCCGACCACTACCAGCAGAACACCCCCATCGGCGACGGCCCCGTGCTGCTGCCCGACAACCACTACCTGAGCACCCAGTCCGCCCTGAGCAAAGACCCCAACGAGAAGCGCGATCACATGGTCCTGCTGGAGTTCGTGACCGCCGCCGGGATCACTCTCGGCATGGACGAGCTGTACAAGGGCAGTGGTCAAGCCGATCAAGAGGCAAAAGAATTGGCTCGTCAAATCAGTTAGGGTTCCGGTGGATCCGGA | Cloning synthesized GFP fragment with 1xVHH05 tag to make pAW-HGP-BiP-GFP-1xVHH05. |
| AR209-GFP-VHH05 2X | AACGGCATCAAGGTGAACTTCAAGATCCGCCACAACATCGAGGACGGCAGCGTGCAGCTCGCCGACCACTACCAGCAGAACACCCCCATCGGCGACGGCCCCGTGCTGCTGCCCGACAACCACTACCTGAGCACCCAGTCCGCCCTGAGCAAAGACCCCAACGAGAAGCGCGATCACATGGTCCTGCTGGAGTTCGTGACCGCCGCCGGGATCACTCTCGGCATGGACGAGCTGTACAAGGGCAGTGGTCAAGCCGATCAAGAGGCAAAAGAATTGGCTCGTCAAATCAGTGGATCGGGCCAAGCGGACCAGGAGGCCAAAGAACTGGCCCGCCAGATCTCGTAGGGTTCCGGTGGATCCGGA | Cloning synthesized GFP fragment with 2xVHH05 tag to make pAW-HGP-BiP-GFP-2xVHH05. |
| AR210-GFP-VHH05 3X | AACGGCATCAAGGTGAACTTCAAGATCCGCCACAACATCGAGGACGGCAGCGTGCAGCTCGCCGACCACTACCAGCAGAACACCCCCATCGGCGACGGCCCCGTGCTGCTGCCCGACAACCACTACCTGAGCACCCAGTCCGCCCTGAGCAAAGACCCCAACGAGAAGCGCGATCACATGGTCCTGCTGGAGTTCGTGACCGCCGCCGGGATCACTCTCGGCATGGACGAGCTGTACAAGGGCAGTGGTCAAGCCGATCAAGAGGCAAAAGAATTGGCTCGTCAAATCAGTGGATCGGGCCAAGCGGACCAGGAGGCCAAAGAACTGGCCCGCCAGATCTCGGGTTCGGGACAGGCCGATCAGGAAGCAAAGGAACTCGCCAGGCAGATCTCCTAGGGTTCCGGTGGATCCGGA | Cloning synthesized GFP fragment with 3xVHH05 tag to make pAW-HGP-BiP-GFP-3xVHH05. |
| AR211-Cloning Twist gBlock R | TCCGGATCCACCGGAACC | Cloning GFP fragment to make pAW-HGP-BiP-GFP-VHH05, pAW-HGP-BiP-GFP-2xVHH05, pAW-HGP-BiP-GFP-3xVHH05, pAW-HGP-BiP-GFP-127D01, pAW-BiP-HGP-GFP-2x127D01, pAW-HGP-BiP-GFP-3x127D01. |
| AR212-Cloning Twist gBlock F | AACGGCATCAAGGTGAACTTCAAG |  |
| AR213-Cloning GFP internal R | CTTGAAGTTCACCTTGATGCCGTTCTTCTGCTTGTCGGCCATGA |  |
| AR345-BiP Fw | AGCCTCCGGACTCTAGAAACATGAAGTTATGCATATTACTGGCCGTCG | Cloning NbVHH05/Nb127D01 and human IgG PCR fragments to make pMT-HGP-v3-Nb127D01-hIgG and pMT-HGP-v3-NbVHH05-hIgG. |
| AR346-hIgG Fw | GGCGGAGGTGGCTCTGGTGG |  |
| AR347-hIgG Rv | TTTCTCCGGATCCACCGGAACCCTTCCCTGGACTTAATGACAATGA |  |
| AR349-127D01 Rv | CCACCAGAGCCACCTCCGCCAGAAGAGACTGTGACCTGCGT |  |
| AR348-VHH05 Rv | CCACCAGAGCCACCTCCGCCTGAGGAGACGGTGACCTGGG |  |
| AR184_pET-26b0-VHH05-Fw | TCTGTGGGTCTCGGATGGCCCAGGTGCAGCTGCAGGAGTC | Cloning NbVHH05 PCR fragment to make pET-26b-Nb127D01-ALFA-His. |
| AR185_pET-26b0-VHH05-Rv | TCTGTGGGTCTCCAGCCTGAGGAGACGGTGACCTGGG |  |
| AR186_pET-26b-127D01-Fw | TCTGTGGGTCTCGGATGGCCGAGGTTCAATTAGTGGAATCTGGCGG | Cloning Nb127D01 PCR fragment to make pET-26b-Nb127D01-ALFA-His. |
| AR187_pET-26b-127D01-Rv | TCTGTGGGTCTCCAGCCAGAAGAGACTGTGACCTGCGTTC |  |
| AR352-PelB | gctgcccagccggcgatggc | Cloning NbVHH05/Nb127D01 to make pET-26b-Nb127D01-HA-His and pET-26b-NbVHH05-HA-His. |
| AR343-127D01 HA Rv | GTGGTGGTGGTGGTGGTGgccagagccggcgtaatcaggcacgtcataggggtagccagagccAGAAGAGACTGTGACCTGCGTTC |  |
| AR344-VHH05-HA Rv | GTGGTGGTGGTGGTGGTGgccagagccggcgtaatcaggcacgtcataggggtagccagagccTGAGGAGACGGTGACCTGGGT |  |
| pAW-nb-F | TACCATCCAGCCTCCGGACTCTAGA GAATTGGGAATTCCAAAATG | Cloning BiP-NbVHH05 or BiP-Nb127D01 fragments to make pAW-BiP-NbVHH05-mCherry-KDEL and pAW-BiP-Nb127D01-mCherry-KDEL. |
| linker-R | TGATCCCCCACCGCTGCCAC |  |
| mCherry-KDEL-F | GTGGCAGCGGTGGGGGATCAatggtgagcaagggcgagga | Cloning mCherry-KDEL fragment to make pAW-BiP-NbVHH05-mCherry-KDEL and pAW-BiP-Nb127D01-mCherry-KDEL. |
| mCherry-KDEL-R | GTTCCTTCACAAAGATCCTGCTAGCTCAcagctcgtccttctcgctacccttgtacagctcgtccatgc |  |
| VHH05-GFP-F | tctgctaccacagcgctagcATGCAGGTGCAGCTGCAGGA | Cloning NbVHH05-GFP fragment to make pAW-CD8-NbVHH05-GFP. |
| pAW-nb-R | TCCTTCACAAAGATCCTGCTAGC TTACTTGTACAGCTCGTCCA |  |
| 127-GFP-F | tctgctaccacagcgctagcATGGAGGTTCAATTAGTGGA | Cloning Nb127D01-GFP fragment to make pAW-CD8-Nb127D01-GFP. |
| pAW-nb-R | TCCTTCACAAAGATCCTGCTAGC TTACTTGTACAGCTCGTCCA |  |
| CD8-mCherry-F | TACCATCCAGCCTCCGGACTCTAGAatggcctcaccgttgacccg | Cloning CD8 fragment to make pAW-CD8-NbVHH05-GFP or pAW-CD8-Nb127D01-GFP. |
| CD8-m-nb-R: | gctagcgctgtggtagcaga |  |
| BiP-VHH05/127tag-F | GAATTCCAAAATGAAGTTATGCATATTACTGGCCGTCGTGGCCTTTGTTGGCCTCTCGCTCGGGTCCCAGGCAGATCAAGAGGCTAAAGAATTGG | Cloning BiP-127D01-VHH05 fragment to make pW10-UAS-BiP-127D01-VHH05. |
| BiP-VHH05/127tag-R | TCAATCCTCGCCTTTCCAGAAATCTTCAAAACTTGATCCCCCACCAGATCTGCTGCCACCTCCGGAAATCTGCCGTGCCAATTCTTTAGCCTCTT |  |
| Akh-V127-F | AGATTTCCGGAGGTGGCAGCCAATTGACCTTCTCGCCGGA | Cloning Akh fragment to make pW10-UAS-BiP-VHH05-Akh-127D01. |
| Akh-V127-R | TCAAAACTTGATCCCCCACCCTCGCGGTGCTTGCAGTCCA |  |
| Dilp2-V127-F | AGATTTCCGGAGGTGGCAGCACGCTCTGCAGTGAAAAGCT | Cloning Dilp2 fragment to make pW10-UAS-BiP-VHH05-Dilp2-127D01. |
| Dilp2-V127-R | TCAAAACTTGATCCCCCACCATTTCTGACCACGGAGCAGT |  |
| Dilp8-V127-F | AGATTTCCGGAGGTGGCAGCAGCTTCTGCTCCCTGGAGCGGA | Cloning Dilp2 fragment to make pW10-UAS-BiP-VHH05-Dilp8-127D01. |
| Dilp8-V127-R | TCAAAACTTGATCCCCCACCGCAGAAGAACTCCTCCTCGCA |  |
| Pvf1-V127-F | AGATTTCCGGAGGTGGCAGCGGCTCCTTGGTGTCCCCGAACAACA | Cloning Pvf1 fragment to make pW10-UAS-BiP-VHH05-Pvf1-127D01. |
| Pvf1-V127-R | TCAAAACTTGATCCCCCACCAACACTGTACAGGGTGGTGTTA |  |
| Upd2-V127-F | AGATTTCCGGAGGTGGCAGCGCGCGACACTTGCGCGACCAGGT | Cloning Upd2 fragment to make pW10-UAS-BiP-VHH05-Upd2-127D01. |
| Upd2-V127-R | TCAAAACTTGATCCCCCACCAGACTCATTGGATCCGCCATCG |  |
| Reptor-bpb-V127-F | ACTCTGAATAGGGAATTGGGAATTCCAAAATGCAGGCAGATCAAGAGGCTAAAGAATTGGCACGGCAGATTTCCggatccggaGCTAGCATGGCTGATATGGAGATACA | Cloning REPTOR-bp-B or REPTOR-bp-C fragments to make pW10-UAS-127D01-REPTOR-bp-B-VHH05, pW10-UAS-127D01-REPTOR-bp-C-VHH05. |
| Reptor-bpb-V127-R | CAAAACTTGATCCCCCACCAGATCTTTCTTGTTTGAGGATTCCGA |  |
| Reptor-V-F | GCAGATTTCCggatccggaGCTAGCATGACAGAGAATCAGCTGTATCCC | Cloning REPTOR fragment to make pW10-UAS-127D01-REPTOR-VHH05. |
| Reptor-V127-R | TCAAAACTTGATCCCCCACCCGACGATTTACGCAGCTCCTCGAG |  |
| pHD-N-3X127-tag-F | AATTGAATTTAGCGGCCGCGAATTCATGAGTTTTGAGGACTTTTGGAA | Cloning 3X127D01 fragment to make pScarlessHD-N-3x127D01-DsRed. |
| pHD-N-3X127-tag-R | ATTATCTTTCTAGGGTTAATGTCTTCTCCTTTCCAAAAGT |  |
| pHD-N-3x127-3xp3-red-F | ACTTTTGGAAAGGAGAAGACATTAACCCTAGAAAGATAAT | Cloning 3XP3DsRed fragment to make pScarlessHD-N-3x127D01-DsRed. |
| pHD-N-3x127-3xp3-red-R: | CTAGTCCTGCAGGTTTAAACGAATTCGGAACCTCCTGAACCACCAG |  |
| pHD-C-3X127-tag-F | AATTGAATTTAGCGGCCGCGAATTCGGAGGTGGCAGCGGCTCCGGC | Cloning 3X127D01 fragment to make pScarlessHD-C-3x127D01-DsRed. |
| pHD-C-3X127-tag-R | TAGTCCTGCAGGTTTAAACGAATTCTTAACCCTAGAAAGATAGTC |  |
| pHD-N-3XVHH-tag-F | AATTGAATTTAGCGGCCGCGAATTCATGCAAGCCGATCAAGAGGCAAA | Cloning 3XVHH05 fragment to make pScarlessHD-N-3xVHH05-DsRed. |
| pHD-N-3XVHH-tag-R | ATTATCTTTCTAGGGTTAATGGAGATCTGCCTGGCGAGTT |  |
| pHD-N-3xVHH-3xp3-red-F | AACTCGCCAGGCAGATCTCCATTAACCCTAGAAAGATAAT | Cloning 3XP3DsRed fragment to make pScarlessHD-N-3xVHH05-DsRed. |
| pHD-N-3x127-3xp3-red-R: | CTAGTCCTGCAGGTTTAAACGAATTCGGAACCTCCTGAACCACCAG |  |
| pHD-C-3XVHH-tag-F | AATTGAATTTAGCGGCCGCGAATTCGGAGGTGGCAGCGGCtGTGGT | Cloning 3XVHH05 fragment to make pScarlessHD-C-3xVHH05-DsRed. |
| pHD-C-3X127-tag-R | TAGTCCTGCAGGTTTAAACGAATTCTTAACCCTAGAAAGATAGTC |  |
| H2av-up-F: | TTGGTGGATCTGGAGGTTCCTGGTTGGTTGGGGTTGACAA | Cloning upstream fragment of H2Av to make pScarlessHD-C-3xVHH05-H2Av-DsRed or pScarlessHD-C-3x127D01-H2Av-DsRed. |
| H2av-up-R: | CCGGAGCCGCTGCCACCTCCGTAGGCCTGCGACAGAATGA |  |
| H2av-down-F: | GACTATCTTTCTAGGGTTAAAGTCGGCAATCGGACGCCTT | Cloning downstream fragment of H2Av to make pScarlessHD-C-3xVHH05-H2Av-DsRed or pScarlessHD-C-3x127D01-H2Av-DsRed. |
| H2av-down-R: | GTTTAAACGAATTCGCCCTTTCGCTCCGTCTCGCGCCACGA |  |
| H2av-sg-F: | GTCGCGATTGCCGACTGGGTTAGT | Sense and anti-sense oligo for sgRNA plasmid. |
| H2av-sg-R: | AAACACTAACCCAGTCGGCAATCG |  |
